# Automated virtual reality therapy to treat needle fears (trypanophobia) in adolescents in England: a proof-of-concept cohort study and a Phase II randomised controlled trial

**DOI:** 10.1016/j.eclinm.2026.104038

**Published:** 2026-07-15

**Authors:** Daniel Freeman, Jason Freeman, Eliot Farmer, Holly Turner, Rupert Ward, Andre Lages Miguel, Matthew Bousfield, Jack Myrick, Aitor Rovira, Helen McShane, Andrew J. Pollard, Felicity Waite, Sinéad Lambe, Amy Povey, Fiona Singleton, Hugo Senra, Ly-Mee Yu, Eve Twivy

**Affiliations:** a Department of Experimental Psychology, University of Oxford, Oxford, UK; b Oxford Health NHS Foundation Trust, Oxford, UK; c The Jenner Institute, Nuffield Department of Medicine, University of Oxford, Oxford, UK; d Oxford Vaccine Group, Department of Paediatrics, University of Oxford, Oxford, UK; e Nuffield Department of Primary Care, University of Oxford, Oxford, UK; f Coimbra Institute for Biomedical Imaging and Translational Research (CIBIT), ICNAS, Faculty of Medicine, University of Coimbra, Coimbra, Portugal

**Keywords:** Adolescents, Needle fears, Blood-injection-injury phobia, Anxiety disorders, Virtual reality (VR)

## Abstract

**Background:**

Fear of needles is distressing in itself but can also lead to avoidance of vaccinations, blood donation, and medical tests and treatments. Working with adolescents with needle fears, we developed an automated virtual reality (VR) therapy. We set out to evaluate its efficacy.

**Methods:**

We conducted an initial proof-of-concept cohort study followed by a Phase II randomised controlled trial. Participants were included if they were 12–15 years old and had significant needle fears that they would like treated. The proof-of-concept testing was a pre- to post-cohort study. To proceed to an RCT an effect size of at least 0.6 on the Injection Phobia Scale – Anxiety (child version) (IPS) was required. The Phase II test was a parallel group, single-blind, randomised controlled trial in one centre in England. Participants were randomly assigned (1:1) to either the VR therapy or no treatment, using a permuted blocks algorithm with randomly varying block size. The VR therapy was provided in approximately three sessions over two to three weeks. The trial assessor was masked to group allocation. Outcomes were assessed at 0, 3 (end of treatment) (primary endpoint), and 6 weeks. The primary outcome was needle fear, assessed by the IPS. Outcome analyses were done in the intention-to-treat population. Moderation and mediation tests were also planned. The RCT was prospectively registered with the ISRCTN registry, ISRCTN74002253.

**Findings:**

From March 26th, 2024, to June 7th, 2024, 12 participants (5 [42%] males; 6 [50%] females, 1 [8%] prefer not to say, mean age 13.4 years [SD = 0.8, range 12–15]; 10 [83%] White, 1 [8%] other, 1 [8%] prefer not to say) were recruited for the cohort study. After the VR therapy, needle fears as assessed by the IPS reduced on average by 15.2 points [95% C.I. 9.2, 21.1, n = 11, Cohen’s d = 1.5]. There were no serious adverse events. The RCT could therefore proceed. From October 28th, 2024, to July 15th, 2025, 60 participants (26 [43%] males; 32 [53%] females, 2 other [3%], mean age 13.4 years [SD = 0.9, range 12–15]; 57 [95%] White, 3 [5%] other) were recruited for the RCT. Compared with no treatment, there was strong evidence that VR therapy led to a reduction in needle fears (adjusted mean difference = −14.07, 95% C.I. −17.40, −10.73, p < 0.001, n = 60, Cohen’s d = 1.34). The benefits were maintained at follow-up (adjusted mean difference = −14.67, 95% C.I. −18.00, −11.33, p < 0.001, n = 60, Cohen’s d = 1.39). There were no serious adverse events.

**Interpretation:**

An automated VR therapy, which is potentially scalable, had efficacy in reducing self-reported needle fears in adolescents. Given that this is an age at which people often have a formative experience of medical procedures, it is an important time to reduce needle fears. The intervention requires testing in a Phase III clinical trial. A key focus would be assessing whether the VR therapy increases vaccine uptake in young people who have initially refused due to needle fears.

**Funding:**

Beryl Alexander Charity and the NIHR Oxford Health Biomedical Research Centre and NIHR Oxford Biomedical Research Centre.

**Research in context**

**Evidence before this study**

Rates of needle fear are greatest in children and young people. A number of vaccinations are given during adolescence, for example to protect against the human papillomavirus (HPV), tetanus, diphtheria, polio, and meningitis. Needle fear can prevent vaccination uptake. We wanted to develop and test an automated virtual reality (VR) exposure therapy to reduce needle fear in young people. The focus was on treating the fear that can lead to distress and avoidance of needle procedures, rather than the use of VR to distract from anxiety or pain during the time of use of needles. We searched PubMed on Nov 30, 2025, with no date or language restrictions, using the terms (“Virtual reality” OR “VR”) AND (“needle fear” OR “fear of needles” OR “trypanophobia” OR “blood-injection-injury”). Six papers were identified. One pilot randomised controlled trial with 43 adults with blood-injection-injury phobia showed that therapist-guided exposure in VR may lead to moderate to large reductions in needle fear. One pilot pre- to post-study with 32 adults reporting needle fear who had a single session of VR exposure indicated potential modest benefits. There were no tests of VR exposure therapy specifically for needle fear in young people. We also searched PubMed on Nov 30, 2025, with no date or language restrictions, using the terms (“needle fear” OR “fear of needles” OR “trypanophobia” OR “blood-injection-injury”) AND (“randomised” OR “randomized”) AND (“children” OR “adolescence” OR “young people”). 16 papers were identified. There were no randomised controlled trials specifically focussed on treating needle fear in young people.

**Added value of this study**

To our knowledge this is the first randomised controlled trial testing the use of an automated virtual reality therapy to treat needle fears in adolescence. To our knowledge it may be the first randomised controlled trial specifically focused on treating needle fear in adolescence. Substantial reductions in needle fear were shown with the VR therapy. Satisfaction rates were high, and side effects were infrequent. The power of the trial to detect mediation and moderation effects was very limited. In mediation analyses the reductions in needle fears with the VR therapy were partly accounted for by reductions in negative cognitions about needles and to a lesser extent by needle-related disgust. There was no evidence, although it should be noted that the trial was small, that the treatment effects were moderated by age, gender, ethnicity, a history of fainting, a phobia diagnosis, or distress from tactile sensations related to needle procedures. Data from a behavioural avoidance task used in the proof-of-concept testing showed a potential large treatment reduction in avoidance of needle stimuli but no other assessments of behavioural change were made.

**Implications of all the available evidence**

An automated VR therapy, delivered using a standalone consumer VR headset, is potentially a scalable and effective treatment for needle fear that could be delivered by vaccination services. Reduction in self-reported needle fear was shown, but behavioural change was not tested in the randomised controlled trial. Evaluation of whether the VR therapy can increase vaccination uptake for those who have initially refused because of needle fear would be valuable.

## Introduction

In 2021 we conducted an epidemiological survey of injection fear in 15,014 UK adults, quota sampled to match the population for age, gender, ethnicity, income, and region.^1^ One in four of the population screened positive for blood-injection-injury phobia. For example, the fear of receiving a hypodermic injection in the arm was rated as intense by 3.6%, considerable by 8.3%, and mild by 31.3%. Fears were significantly higher in younger people. For example, in adults under the age of 30, the fear of receiving a hypodermic injection in the arm was rated as intense by 9.3%, considerable by 19.0%, and mild by 38.4%. This is consistent with previous research in which needle fears have been found to be at their highest in children and adolescents.^2^^,^^3^ Prevalence estimates of needle fear in adolescents range from 20 to 50%.^3^ We conducted our UK survey to test the potential contribution of needle fear to COVID-19 vaccination hesitancy. The population attributable fraction (PAF) indicated that if blood-injection-injury phobia were absent then this may prevent 11.5% of all instances of COVID-19 vaccine hesitancy.^1^ It is an illustration of the impact of needle fear.

For people with needle fears, needles have become associated with threat. People fear, for example, that the procedure will go wrong (e.g., the needle may snap or they may be given the wrong injection), or it will be very painful, or that they will have a panic attack or be unable to cope. Uniquely among anxiety disorders, part of a common physiological response pattern for needle phobia is a sharp drop in heart rate and blood pressure that can lead to fainting.^4^ People therefore also worry about fainting and its consequences. Disgust reactions may be a further contributory factor to needle, blood, and injury fears.^5^^,^^6^ Needle fear is treatable with psychological therapy. The aim is for the person to develop new benign associations with needles and learn that they can cope when around needle-related stimuli. Although the quality of the trial evidence is generally rated as low, exposure therapy (graded presentation of needle-related stimuli) and applied tension (learning to raise blood pressure when early signs of a drop in blood pressure are noticed) likely produce large reductions in blood-injection-injury fears.^7^, ^8^, ^9^ These approaches likely work in young people.^10^^,^^11^ However, availability of such treatment is extremely limited.

Virtual reality (VR) may provide a way to increase the availability of effective treatment for needle fears. There are three key reasons. First, delivery of therapy can be automated within VR. A therapist is not required, thereby removing a potential key limiter in provision of treatment. Second, VR can be a powerful therapeutic medium. People find it easier to approach feared stimuli in VR because they know they are not real, yet the learning made still transfers to the real world. Finally, VR allows us to present stimuli in ways that are therapeutic but impossible in the real world, potentially increasing treatment efficacy. VR has already shown promise for treating needle fear. A pilot randomised controlled trial with 43 adults with blood-injection-injury phobia showed that a 90-min therapist-guided VR intervention may reduce fear of injections with moderate to large effects.^12^ There have been no other RCTs of VR interventions for needle fear. There is a separate literature on the use of VR as a distraction technique during the administration of needle procedures.^13^^,^^14^

We have previously developed several successful automated VR therapies by including a virtual coach within the programme.^15^, ^16^, ^17^ With adolescents with needle fears, we developed an automated VR therapy for needle fears. We wanted to target the anxiety that can lead to distress and avoidance of needle procedures, rather than using VR to distract from anxiety or pain during such procedures.^13^^,^^14^ We focussed on younger ages for three reasons. First, because this is when needle fears are most prevalent. Second, because several vaccinations are given to 12–14 year-old children in the UK (e.g., the human papillomavirus vaccine (HPV) that protects against various cancers, Td/IPV that helps protect against tetanus, diphtheria and polio, and the MenACWY vaccine to protect against meningitis and sepsis). Finally, early experiences of positive healthcare provision are likely to have long-lasting benefits.

Our overall aim was to evaluate the efficacy of the automated VR therapy for needle fears in adolescents. In initial proof-of-concept testing, the objectives were to evaluate whether the VR therapy: is associated with a reduction in needle fear; has high usability ratings, high satisfaction ratings, and few side effects; and is associated with reduction in anxiety and avoidance of real needle-related stimuli (using a behavioural avoidance test). If the proof-of-concept testing was indicative of clinical benefits, using a pre-defined criterion, we would advance to a randomised controlled trial to test efficacy. In the RCT we wanted to evaluate whether the VR therapy: reduces needle fear at end of treatment (primary hypothesis); produces lasting reductions after the end of treatment; and has high satisfaction ratings. In addition, we aimed to conduct an initial examination of how the treatment may work by testing whether the VR therapy is associated with reductions in fearful cognitions and disgust reactions and whether these changes may mediate change in needle fear. We also wanted to conduct an initial examination of whether there are moderators (age, ethnicity, gender, history of fainting, phobia diagnosis, or distress from tactile sensations related to needle procedures) of the VR therapy effects (i.e., test whether some people particularly benefit). Finally, we planned to investigate side effects, level of fear in VR, and occurrence of vasovagal symptoms (potential signs of fainting).

## Methods

### Study design and participants

The protocol is provided in Supplementary materials. We conducted pre- to post-treatment proof-of-concept testing of the VR therapy in an initial cohort study in one centre in England. The RCT was a parallel-group, single-blind trial in one centre in England. Assessments were done at 0, 3 (end of treatment), and 6 weeks post randomisation. Participants in the control arm were able to have the VR therapy after completion of the 6-week follow-up assessment. The RCT was registered prospectively on 6th August, 2024 (ISRCTN74002253).

The trial was conducted in one centre (Oxford) with the University of Oxford and Oxford Health NHS Foundation Trust (OHFT) as the research sites. The principal method of recruitment was via the Oxford Health Oxfordshire School Aged Immunisation Service (SAIS), which sent out a letter describing the study to parents or guardians of pupils who were due to be vaccinated. Referrals were also sought via the Berkshire School Aged Immunisation Team (Berkshire Healthcare NHS Foundation Trust), Buckinghamshire School Aged Immunisation Team (Buckinghamshire Healthcare NHS Trust), and local radio advertisements.

Eligible participants for all testing were aged 12–15 years old (up to 16th birthday); had significant needle fears that they would like treated; were willing and able to give informed assent for participation in the study; and had a parent or guardian willing and able to give informed consent for their child’s participation. Significant needle fears were assessed by four questions: Are you very scared of needles? Would you be very worried about having an injection? Would you be very worried about having a blood test? Would you like to be less scared of needles? Potential participants needed to answer Yes to the first and last questions and at least one of the other two questions. Exclusion criteria were photosensitive epilepsy or significant visual, auditory, or balance impairment that would make use of VR inappropriate; current engagement in any other psychological treatment for needle fear; and command of English inadequate for engaging in the therapy or completing the assessments.

### Randomisation and masking

All participants in the proof-of-concept testing received the intervention and there was no randomisation. These assessments were conducted unblinded. Participants in the randomised controlled trial were randomised after completion of the baseline assessment. Eligibility calls were made by the trial co-ordinator, research assessor, or assistant psychologist. Informed consent was taken by the research assessor or trial co-ordinator and the baseline assessment was conducted by the research assessor or trial co-ordinator. Participants were randomly allocated (1:1) to the VR therapy or no treatment. Randomisation was done by the trial co-ordinator using a validated online system (Sealed Envelope, www.sealedenvelope.com). Participants were randomised individually using the online system after each baseline assessment was completed. Randomisation used a permuted blocks algorithm, with randomly varying block size. The trial assessor in the RCT was masked to group allocation. Participants were asked not to tell the assessor whether they had had the VR therapy and there was careful consideration of room use to prevent blind breaks. There was one break of blind (one person had both follow-ups conducted unblinded).

### Procedures

The evaluation tested VR for Needle Fears, a virtual-reality application recommended for adolescents reporting significant needle fears. It is intended to reduce needle fears. It is a cognitive-behavioural exposure and applied tension intervention. The treatment content was designed by the Oxford Cognitive Approaches to Psychosis (O-CAP) research group at the University of Oxford, with young people with lived experience taking part via an informant design process.^18^ This included Young People’s Advisory Group meetings to contribute to the design of the environments, characters, and activities, in addition to usability testing. Nurses in school immunisation teams were consulted on the medical content. The treatment was programmed by our team at the University of Oxford. The VR therapy is a UKCA marked, Class I medical device (standalone software as a medical device). The application was built using the Unity 3D platform and was run on a Meta Quest 3 VR headset.

The programme takes approximately two to 3 hours to complete within VR. There are breaks between immersions in VR. The programme can be done in a half day or several shorter meetings on different days. The tasks are completed while sitting down (in order to limit any potential adverse effects from fainting). A team member (assistant psychologist or clinical psychologist) is present in the room while the programme is used. A parent may also be present too. The team member sets up and supports the use of the VR, reinforcing the therapy principles, and ensuring participant safety. Key VR treatment components include: psychoeducation; introduction to the applied tension technique to prevent fainting; graded exposure to feared situations; modelling by computer characters; encouragement and positive reinforcement. The VR therapy is set within a school environment. There is a virtual coach (called Farah), who provides information about needle fears and overcoming them, encouragement, and guidance about what to do in each part of the programme. There are five levels of exposure (looking at needles, picking up needles, using needles, observing needle procedures, and receiving needle procedures), which are summarised in Table 1. Several of the levels include exposure to blood. The exposure levels take place in various rooms along the school’s main corridor. The tasks build up from seeing virtual needles in display cases to piercing balloons with needles to injecting a penguin to watching a person being vaccinated to a final gym-set school scene, in which a nurse gives a vaccination into the user’s virtual arm. The tasks were designed to be engaging for users, and several would not be possible in real world treatment provision (e.g., taking blood from a giant). Time in VR was recorded by the therapy software programme.

**Table 1:** The activities within the automated VR therapy.

Level of interaction with VR needle-related stimuli	School setting	Aim	Activities
1. Looking at needles	School museum	Require user to look at needle-related objects. Includes education about different types of needles and their purpose.	‘Treasure hunt’ type activities involving looking for needles and related medical equipment: • Opening cabinet drawers to find needles from different historical periods. • Finding specific needle-related medical objects in a rotating display case.
2. Picking up needles	School museum	Requires user to pick up and hold needles. Also includes education about different types of needles and their purpose.	User is presented with six realistic needles of differing sizes used for different procedures. User has to hold each needle in different flames until the needle glows a specific colour and then sorts them into colour-coded holders.
3. Using needles	School laboratory	Requires the user to utilise the function of needles (i.e., piercing, extracting, injecting). Also includes an explanation of what happens during needle procedures.	There are three areas within this level for completion of three categories of activities. Inanimate objects: • Piercing virus-shaped air balloons with needles. • Extracting liquid from a blood orange. • Injecting jam into doughnuts. Animal: • Giving penguins medicine via an injection. (Virtual) Humans: • Finger prick on self (virtual hand). • Taking blood from a giant’s arm using venepuncture method.
4. Observing a needle procedure	School sports hall	Requires user to observe realistic needle procedures being performed by a virtual human health professional (VR re-creation).	User waits in line with other students to receive a vaccination. User teleports gradually from back of queue to next in line. User watches another (virtual) student receive an injection (including consent, preparation of vaccine, injection, check-in afterwards).
5. Receiving a needle procedure	School sports hall	Requires users to receive realistic needle procedures performed by a (virtual) health professional (on a virtual arm and hand for the user).	User receives an intramuscular injection (vaccination) and a venepuncture blood test from a nurse with other students watching. Each of these procedures include the following steps: • Consent process (i.e., nurse providing information about procedure and asking questions). • If user consents, they place arm into correct position indicated by a hologram. Detection is in place if user moves arm to abort the procedure. • Preparation of the procedure (e.g., safety cap removed and tourniquet applied for blood test). • User can see needle going into virtual body (upper arm for injection and vein in arm for blood test). Sees blood coming out into vials for the blood test. • Cotton wool/tape is applied.

### Outcomes

At baseline, key demographic information was collected (age, gender, ethnicity) and the Specific Phobia subsection of the Anxiety Disorder Interview Schedule (Child Version)^19^ was used to determine whether participants met diagnostic criteria for blood-injection-injury phobia and whether there was a history of fainting.

The primary outcome in both the proof-of-concept testing and the RCT was the child version of the Injection Phobia Scale – Anxiety.^20^ It is an 18-item self-report measure, with each item asking about the extent of worry about specific needle-related situations. Each item is rated on a 5-point scale (0 = not worried to 4 = extremely worried). Higher scores indicate higher levels of needle fear. Items have strong face validity (e.g., How worried would you feel about looking at a picture of a needle/watching another person have a blood test/having a blood test/getting a vaccination). The internal consistency in the current study was high (Cronbach’s alpha = 0.86, n = 72). IPS scores significantly correlated with the behavioural avoidance task scores obtained in the proof-of-concept study (r = −0.71, p = 0.014, n = 11). As a secondary outcome, the proof-of-concept evaluation included a behavioural avoidance task (BAT). This involved developing with each participant a six-step fear hierarchy with real injection-related stimuli, in particular utilising a medical phlebotomy practice kit. A score of one was given for each step that the participant completed, meaning that total scores could vary between 0 and 6, and lower scores indicated greater avoidance. The BAT was not used in the RCT. Secondary outcomes for the RCT were the 16-item Needle Cognitions Questionnaire (e.g., “The needle will be painful” “The needle will snap in my arm” “I will be overwhelmed with fear/anxiety”) (see Supplementary materials) and the Disgust Emotion Scale for Children – Injections and Blood Draws Subscale.^21^ Higher scores on each measure mean more negative cognitions about needles and greater levels of disgust about injections, respectively. We created a five-item Tactile Sensations Questionnaire (see Supplementary materials) that was also used at baseline to assess how upset the person would feel by various parts of an injection procedure, for example, the feeling of a needle in their body, their skin/veins being touched, the tightness of a tourniquet, and the cold/wet feel of an alcohol wipe. Higher scores indicate greater distress. As our VR therapy used no haptics, it would be plausible that the presence of such sensitivities would go unaddressed in treatment and therefore limit improvement. This scale was therefore included as a potential moderator of outcomes, but has not had a psychometric evaluation.

Measures used for end of VR therapy unblinded data collected on satisfaction, potential side effects, level of fear elicited in VR, and occurrence of vasovagal symptoms (e.g., faintness, weakness) were: Child Treatment Satisfaction (Modified wording)^22^; Modified Oxford – VR Side Effects Scale^23^; Visual analogue scale – fear while using VR (0 (not at all scared) to 10 (Extremely scared)); and the four-item Blood Donation Reaction Inventory^24^ with potential scores ranging from 0 (no vasovagal symptoms) to 20 (extremely high levels). The proof-of-concept testing also included administration of an adapted 10-item VR usability scale developed and previously used by our group.^25^^,^^26^

Any reporting by participants or their parent or guardian of serious adverse events (SAE) (see standard regulatory definition in the trial protocol) was recorded. There was an independent clinician to rate whether any SAE was related to the VR therapy or trial procedures.

### Statistical analysis

The target sample size for the proof-of-concept testing was 12 participants. This was because we expected large effects and was a similar size to our previous early-stage testing of interventions.^17^^,^^27^ Paired t-tests were used to estimate mean differences and confidence intervals for the pre-post change in needle fear. p-values would not be reported. Effect sizes (Cohen’s d) were calculated by dividing the change score by the standard deviation of the baseline average score. Phase II (RCT) would not proceed unless there was evidence of at least a moderate effect size (d = 0.6) reduction in needle fears. General guidelines for interpretation of Cohen’s d are: small effect: d = 0.2; medium effect: d = 0.5; large effect: d ≥ 0.8; very large effect: d ≥ 1.2; and huge effect: d ≥ 2.0.^28^

For the RCT, a statistical analysis plan (see Supplementary materials) was agreed before the analysis was conducted. The target sample size for the RCT was 60 individuals, which would enable the trial to detect a large standardised treatment effect (Cohen’s d = 0.87) with 90% power at a 5% level of significance (2-sided). Analyses were done after the last follow-up assessment was completed (with no interim analyses). The primary estimand using the treatment policy strategy was defined as the mean difference in Injection Phobia Scale-Anxiety (IPS) at three weeks from randomisation for all randomised participants. Treatment effects on the primary outcome were estimated using a linear mixed model fitted to the Injection Phobia Scale-Anxiety score at all follow-up points. Fixed effects were the baseline assessment for the outcome, treatment, time, and time × treatment interactions. Participants were included as a random intercept. An unstructured covariance matrix structure for random-effects and a diagonal covariance matrix structure for residuals were adopted. Marginal treatment effects were estimated for the 3-week timepoint (primary outcome) and 6-week timepoint (secondary outcome), and reported separately as adjusted mean differences in scores between the groups with 95% confidence intervals and 2-sided p-values. The same model approach was adopted for secondary outcome analyses. Cohen’s d effect sizes were calculated as the adjusted mean difference of the outcome divided by the sample standard deviation of the outcome at baseline. To test the moderation hypotheses, the analysis model for the primary outcome was extended to include as fixed effects the putative moderator and its interaction with treatment. The coefficient of the interaction tests whether there is a differential treatment effect across levels of the moderator variable.

Mediation analysis was performed using the counterfactual framework of causal inference.^29^, ^30^, ^31^ It was implemented using the mediation algorithms proposed by Imai and colleagues,^29^ through the mediation R package.^32^ Confidence intervals were calculated using 2000 Monte Carlo draws for quasi-Bayesian approximation.

Presentation of SAE, usability, satisfaction, side effects, fear, and vasovagal symptoms was descriptive.

### Ethics

The trials received approval from an NHS Research Ethics Committee (NHS South Central–Oxford B Research Ethics Committee, ref 23/SC/0420). Given that all participants were under 16, written or oral informed consent was obtained from the parent or guardian. Written or oral informed assent from the participants was also obtained. A Young People’s Advisory Group fed back on all participant-facing documentation.

### Changes to the protocol

Before the proof-of-concept testing was conducted, the original target size for the RCT was 100 individuals, which would enable the trial to detect a standardised treatment effect of moderate size (d∼0.6) with 90% power at a 5% level of significance (2-sided). Given the size of effects observed in the proof-of-concept testing, the power calculation was amended on July 11th, 2024, before the start of the RCT.

### Role of the funding source

The funders of the study had no role in study design, data collection, data analysis, data interpretation, or writing of the report. The statisticians (L-MY, HS), trial co-ordinator (ET), and chief investigator (DF) had full access to all the data and the corresponding author (DF) had final responsibility for the decision to submit for publication.

## Results

Recruitment for the proof-of-concept trial took place from March 26th, 2024, to June 7th, 2024. Final follow-up data were collected on June 26th, 2024. 14 young people were assessed for eligibility. One individual was unreachable, one individual did not complete the eligibility assessment, and 12 young people were enrolled and took part. The average age was 13.4 (SD = 0.79) years. 5 (41.7%) were male, 6 were female (50.0%), and 1 (8.3%) preferred not to say. 10 (83.3%) were of White ethnicity, 1 other (8.3%), and 1 (8.3%) preferred not to say. All 12 (100%) met diagnostic criteria for blood-injection-injury phobia. 6 (50.0%) felt as if they were going to faint or had fainted, while 5 (41.7%) did not, and 1 (8.3%) reported other.

The average number of VR therapy sessions attended in the proof-of-concept testing was 2.6 (SD = 0.7) (n = 12). Eight people attended 3 sessions, three people attended two sessions, and one person attended one session. The average time spent in VR was 126.11 (SD = 31.08) minutes (minimum = 46.8, maximum = 165.6). For these patients a clinical psychologist was present in the room. How scared the participants felt in VR in each session (rated on a 0–10 visual analogue scale) were: first session mean score = 3.93 (SD = 1.67) (minimum = 1.2, maximum = 6.3) (n = 11); second session mean score = 5.83 (SD = 2.06) (minimum = 1.5, maximum = 8.0) (n = 11); third session mean score = 4.87 (SD = 2.21) (minimum = 2.0, maximum = 8.5) (n = 8). Levels of vasovagal symptoms assessed by the Blood Donation Reaction Inventory (potential score range 0–20) were low: first session mean score = 1.55 (SD = 2.70) (minimum = 0, maximum = 9) (n = 11); second session mean score = 1.55 (SD = 2.30) (minimum = 0, maximum = 6) (n = 11); third session mean score = 0.50 (SD = 1.07) (minimum = 0, maximum = 3) (n = 8).

One young person dropped out of the study after one session of the VR therapy. Eleven young people provided before and after data. Injection Phobia Scale scores significantly reduced across the group from 40.36 (SD = 10.15) (n = 11) to 25.18 (SD = 9.94) (n = 11). The mean IPS reduction was 15.18 (95% C.I. 9.24, 21.13), Cohen’s d = 1.50. This improvement meant that the RCT could be conducted. Behavioural avoidance task (BAT) scores improved from 3.73 (SD = 2.05) (n = 11) to 5.36 (SD = 1.12) (n = 11). The mean improvement in avoidance was 1.64 (95% C.I. 0.58, 2.69), Cohen’s d = 0.8.

**Fig. 1:**
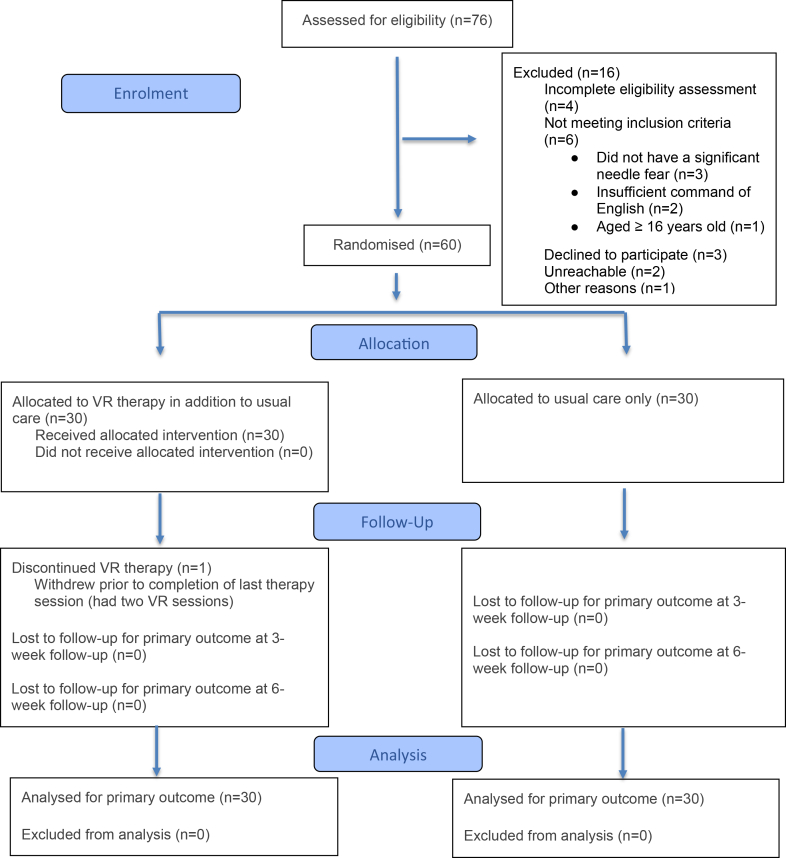
CONSORT 2025 flow diagram.

Recruitment for the RCT took place from October 28th, 2024, to July 15th, 2025. Final follow-up data were collected on August 26th, 2025. 76 people were assessed for eligibility and 60 were enrolled and randomly assigned to the VR therapy (n = 30) or no treatment (n = 30) (see Fig. 1). Characteristics of the participants by group are summarised in Table 2.

**Table 2:** Basic demographic and clinical data.

	VR therapy group (n = 30)	Control group (n = 30)
Mean age in years (SD)	13.23 (0.90)	13.50 (0.86)
Gender (n)		
Male	14	12
Female	15	17
Other	1	1
Ethnicity (n)		
White	29	28
Other	1	2
Blood-injection-injury phobia from Anxiety Disorder Interview Schedule (n)		
Yes	28	28
No	2	1
Missing	0	1
History of feeling faint or fainting (n)		
Yes	18	12
No	10	16
Other	2	1
Missing	0	1

The average number of VR therapy sessions attended in the RCT was 3.0 (SD = 0.6) (n = 30). Four patients had two sessions of VR therapy, 21 had three sessions, and 5 had four sessions. Total time for the meetings, including time in VR, was an average of 262.2 (SD = 65.4) minutes. The average time spent in VR was 152.8 (SD = 35.5) minutes (minimum = 71, maximum = 227). For the treatment group, a psychology assistant (n = 22), clinical psychologist (n = 3), or both (n = 5) were in the room. How scared the participants felt in VR in each session (rated on a 0–10 visual analogue scale) were: first session mean score = 3.27 (SD = 2.11) (minimum = 0, maximum = 7.2) (n = 29); second session mean score = 5.01 (SD = 2.43) (minimum = 0, maximum = 9.9) (n = 30); third session mean score = 4.18 (SD = 2.95) (minimum = 0, maximum = 10) (n = 26); fourth session mean score = 3.86 (SD = 2.04) (minimum = 1.5, maximum = 6.7) (n = 5). Levels of vasovagal symptoms assessed by the Blood Donation Reaction Inventory (potential score range 0–20) were low: first session mean score = 2.00 (SD = 1.89) (minimum = 0, maximum = 7) (n = 29); second session mean score = 2.00 (SD = 1.97) (minimum = 0, maximum = 7) (n = 30); third session mean score = 1.31 (SD = 1.67) (minimum = 0, maximum = 5) (n = 26); fourth session mean score = 1.20 (SD = 0.84) (minimum = 0, maximum = 2) (n = 5).

There were no missing outcome data. Scores were stable in the control group but there were clear reductions observed for the VR treatment group (see Table 3). Compared with the control group, the VR therapy group had a significant reduction in needle fears at 3 weeks (IPS adjusted mean difference = −14.07, 95% CI –17.40 to −10.73; Cohen’s d = 1.34; p < 0.001) that was maintained at the 6-week follow-up (IPS adjusted mean difference = −14.67, −18.00 to −11.33; Cohen’s d = 1.39; p < 0.001). The number of people in the VR therapy group who had at least a reduction by one third in their injection fear score at 3 weeks was 19 (63.3%) compared to 0 (0%) in the control group. Compared to the control group, the VR therapy group also had significant reductions in needle cognitions at both time-points and moderate reductions in needle-related disgust at both time-points.

**Table 3:** Summary statistics for the primary and secondary outcomes.

	Control group		VR therapy group		Cohen’s d [95% C.I.]	Estimated Treatment Effect^a^ [95% C.I.]	p-value^a^
	n	Mean (SD)	n	Mean (SD)			
Injection phobia scale (primary outcome)							
Baseline	30	40.06 (10.15)	30	40.03 (9.59)			
3-weeks (end-of-treatment)	30	39.63 (11.77)	30	25.53 (9.08)	1.34 [0.77, 1.90]	−14.07 [−17.40, −10.73]	<0.001
6-weeks (follow-up)	30	39.40 (11.91)	30	24.70 (9.11)	1.39 [0.82, 1.95]	−14.67 [−18.00, −11.33]	<0.001
Needle cognitions questionnaire							
Baseline	30	31.23 (12.22)	30	33.17 (12.48)			
3-weeks (end-of-treatment)	30	31.37 (13.31)	30	21.40 (10.92)	0.82 [0.29,1.34]	−11.90 [−16.07, −7.73]	<0.001
6-weeks (follow-up)	30	32.30 (14.66)	30	19.27 (10.55)	1.02 [0.48, 1.56]	−14.97 [−19.14, −10.79]	<0.001
Disgust emotion scale							
Baseline	30	12.17 (6.11)	30	13.67 (4.81)			
3-weeks (end-of-treatment)	30	12.77 (7.56)	30	9.03 (4.95)	0.58 [0.06, 1.10]	−5.23 [−7.14, −3.32]	<0.001
6-weeks (follow-up)	30	12.53 (7.80)	30	8.13 (4.83)	0.68 [0.15, 1.20]	−5.90 [−7.81, −3.99]	<0.001

a Results from random-intercept mixed-models, for the interaction Randomised group x Assessment.

The mediation models at 3 weeks and 6 weeks are presented in Table 4. At 3 weeks, reductions in needle cognitions non-significantly mediated 36% of the treatment effect (p = 0.12) and reductions in needle-related disgust non-significantly mediated 23% of the treatment effect (p = 0.25). At 6 weeks, reductions in needle cognitions significantly mediated 42% of the treatment effect (p = 0.03) and reductions in needle-related disgust non-significantly mediated 30% of the treatment effect (p = 0.12). There was no evidence that the treatment effects were moderated by age (p = 0.988), gender (p = 0.138), ethnicity (p = 0.128), a history of fainting (p = 0.520), a phobia diagnosis (p = 0.509), or distress from tactile sensations related to needle procedures (p = 0.278) (see Supplementary materials).

**Table 4:** Mediation models with the primary outcome (needle fear) at 3 weeks and 6 weeks.

	Mediator 1: needle cognitions	Mediator 2: disgust	Mediator 1: needle cognitions	Mediator 2: disgust
	Estimate [95% CI]	Estimate [95% CI]	p-value	p-value
3-weeks timepoint				
Average causal mediation effects (control)	−2.77 [−6.97, 1.19]	−1.23 [−4.63, 2.08]	0.18	0.45
Average causal mediation effects (treated)	−2.51 [−5.95, 0.57]	−1.72 [−5.02, 1.19]	0.12	0.24
Average direct effects (control)	−4.51 [−8.17, −1.10]	−5.40 [−8.95, −1.94]	0.005	0.002
Average direct effects (treated)	−4.25 [−7.80, −0.80]	−5.88 [−9.28, −2.42]	0.02	<0.001
Total effect	−7.02 [−11.95, −2.24]	−7.11 [−11.55, −2.61]	0.003	<0.001
Proportion mediated (control)	0.40 [−0.35, 0.84]	0.17 [−0.58, 0.53]	0.17	0.45
Proportion mediated (treated)	0.36 [−0.18, 0.73]	0.23 [−0.31, 0.59]	0.12	0.25
Average causal mediation effects (average)	−2.64 [−6.22, 0.78]	−1.48 [−4.70, 1.59]	0.15	0.35
Average direct effects (average)	−4.38 [−7.91, −1.13]	−5.64 [−9.00, −2.29]	0.009	<0.001
Proportion mediated (average)	0.38 [−0.24, 0.76]	0.20 [−0.43, 0.53]	0.14	0.35
6-weeks timepoint				
Average causal mediation effects (control)	−4.17 [−8.05, −0.65]	−2.15 [−5.25, 0.70]	0.02	0.14
Average causal mediation effects (treated)	−3.99 [−7.73, −0.65]	−2.94 [−6.97, 0.71]	0.03	0.12
Average direct effects (control)	−5.58 [−9.15, −2.18]	−6.62 [−10.21, −3.18]	0.001	<0.001
Average direct effects (treated)	−5.39 [−8.60, −2.02]	−7.40 [−10.85, −4.00]	0.001	<0.001
Total effect	−9.57 [−14.34, −4.90]	−9.56 [−14.17, −4.77]	<0.001	<0.001
Proportion mediated (control)	0.44 [0.10, 0.74]	0.22 [−0.11, 0.47]	0.02	0.14
Proportion mediated (treated)	0.42 [ 0.09, 0.69]	0.30 [−0.11, 0.47]	0.03	0.12
Average causal mediation effects (average)	−4.08 [−7.59, −0.70]	−2.55 [−5.85, 0.66]	0.02	0.13
Average direct effects (average)	−5.48 [−8.92, −2.22]	−7.01 [−10.47, −3.71]	<0.001	<0.001
Proportion mediated (average)	0.43 [0.11, 0.69]	0.26 [−0.11, 0.52]	0.02	0.13

There were no SAEs reported in the proof-of-concept testing or the RCT. No person fainted during any VR session. Usability ratings, collected in the proof-of-concept testing, were high (see Supplementary materials). For example, participants rated that it was easy to know what to do in any given VR situation and to understand the coach’s instructions. Satisfaction with the VR therapy is summarised in Table 5. All but two adolescents across the evaluations thought that the VR therapy had been helpful. One out of 40 participants disagreed that the virtual reality therapy helped them to cope better with the fear. Occurrence of side effects in VR are summarised in Table 6. Data were obtained from 11 out of 12 people in the proof-of-concept testing and 29 out of 30 people in the RCT. In the proof-of-concept testing, eight people reported no side effects and three people reported one side effect. In the RCT, 25 participants reported no side effects, two people reported one side effect, and two people reported two side effects. The most commonly reported side effect was a headache.

**Table 5:** VR therapy satisfaction ratings.

	Strongly disagree n	Disagree n	Neutral n	Agree n	Strongly agree n
Proof-of-concept testing (n = 11)					
Overall, my virtual reality therapy was helpful	0	0	0	7	4
My virtual reality therapy helped me to cope better with my fear	0	0	0	7	4
I would recommend this virtual reality therapy to a friend who had similar fears	0	0	2	4	5
Randomised controlled trial (n = 29)					
Overall, my virtual reality therapy was helpful	0	0	2	15	12
My virtual reality therapy helped me to cope better with my fear	0	1	4	11	13
I would recommend this virtual reality therapy to a friend who had similar fears	0	0	3	10	16

**Table 6:** Side effects from use of VR.

Modified Oxford – VR Side Effects Scale items	Proof of concept (n = 11), n		RCT (n = 29), n	
	Yes	No	Yes	No
The headset made me feel trapped and I had a panic attack (sudden and intense fear with physical symptoms).	0	11	0	29
While I was wearing the headset, I walked into something and hurt myself.	0	11	0	29
While I was wearing the headset, I fainted and hurt myself.	0	11	0	29
While I was wearing the headset, I fell and hurt myself.	0	11	0	29
I couldn’t fully concentrate on the session because I was constantly thinking about crashing into something.	0	11	0	29
Wearing the headset caused me pain for quite some time after the session had finished.	0	11	1	28
Wearing the headset left me with worrying/upsetting marks on my face for quite some time.	0	11	0	29
After wearing the headset, I felt so unsteady that I had difficulties walking.	0	11	0	29
Using the headset made my eyes so tired that I couldn’t see properly.	0	11	1	28
Using the headset gave me a lasting headache.	1	10	2	27
While using VR, I felt so sick that I had to stop.	1	10	1	28
While using VR, I felt so faint that I had to stop.	1	10	0	29
For hours after using VR, I felt sick/unwell.	0	11	1	28
VR made me throw up.	0	11	0	29
Going into the VR environments made me have panic attacks.	0	11	0	29
The virtual coach was very unhelpful and put me off the therapy.	0	11	0	29
The therapy got too hard, too quickly, and I felt like I had failed.	0	11	0	29

## Discussion

We sought an initial indication that an automated VR therapy for needle fears may have efficacy for adolescents. If it did, we would conduct an early Phase II randomised controlled trial. There can be a tendency to rush to RCT evaluations. We wanted to be confident that the VR therapy was likely to have efficacy before proceeding to a full trial. The proof-of-concept testing indicated a potentially substantial effect size benefit in reducing needle fears. This effect size was confirmed in the RCT involving sixty young people with significant needle fears. The benefit of the two and a half hours in VR (over three meetings typically) persisted at the six-week follow-up. Just over three out of every five adolescents showed very good treatment responses to the intervention. Usability of the programme was high and it was observed that the adolescents very quickly understood what to do. It was also found that the VR needle-related stimuli, as intended, elicited anxious responses that were not overwhelming but that the adolescents were then able to overcome. Satisfaction rates were very good and the occurrence of side effects relatively rare. The automated VR therapy is easy to use, brief, popular, and efficacious. Potentially, it is highly implementable.

The mediation and moderation tests in the randomised controlled trial can be considered initial examinations due to the small participant group size. Nevertheless, there were indications that the treatment partially worked via reducing negative cognitions about needles (fear of pain, harm, and distress) and disgust about needles and blood. Approximately 40 percent of the treatment effect may be accounted for by reductions in negative cognitions and approximately 25 percent by reductions in disgust. Despite the small sample size, the mediation effects were significant for needle cognitions at the 6-week follow-up. Encouragingly there were no indications of moderators of the treatment effects. For example, effects did not differ by age or gender. It also did not matter for this VR therapy if the individual’s concerns were rooted in physical sensations, despite the absence of haptics. These findings indicate that the VR therapy may well have general applicability for young people with needle fears. However a much larger trial size, with greater diversity, will be needed to have confidence that there are no moderation effects by any socio-demographic factor.

There are a number of important study limitations. The size of the trial was small. Recruitment of participants was from three counties in England and would not have been representative of the wider population. There was no placebo control group to determine that the exposure to needle-related stimuli in VR caused the fear reduction. Part of the treatment effect shown in this trial will have resulted from expectations of an improvement with a credible intervention. Inclusion of such a control group could have potentially enabled participants to be blinded to condition too. The participant group size would also have limited the opportunity to observe significant side effects or vasovagal reactions. It is plausible that a small minority of people will experience significant simulator sickness from use of VR and that side effects such as headaches could limit uptake to a degree. Similarly it is likely that a small minority of people could get very strong vasovagal responses and actually faint. The length of the follow-up was small so it is unknown how long the treatment effects may last. However, the short-term beneficial effects of exposure therapies for anxiety disorders have been found to extend considerably.^33^, ^34^, ^35^ Although the proof-of-concept testing of the VR treatment indicated a large reduction in avoidance of needle stimuli, a behavioural assessment task was not included in the randomised controlled trial due to practical reasons. Needle fear is strongly associated with behavioural avoidance, and such a large reduction in needle fear should bring change in behaviour, but we cannot be sure that the treatment did change real-world behaviours. Anecdotally a number of parents contacted us afterwards to say that their child had successfully had a vaccination or blood test that they had previously been unable to have. They attributed this to the VR intervention. However, we did not select for vaccination refusal in this study or assess whether uptake was affected. A future trial could recruit adolescents who have avoided vaccination due to needle fear and test whether the VR therapy increases uptake and thus potentially prevents future illness. The VR programme could also be adapted for adults and the efficacy evaluated both for reduction in needle fear and greater uptake of medical tests and procedures.

## Contributors

DF was the chief investigator, conceived and designed the study, obtained the funding, led the VR therapy design, and wrote the paper. ET contributed to the design of the study and was the trial co-ordinator and day-to-day clinical lead. DF, ET, JF, RW, MB, ALM, AR, FW, and SL contributed to the therapy design. HM, AJP, AP, and FS advised on the project. AP and FS helped facilitate the conduct of the trial. RW, MB, ALM, and AR developed the software. ET and EF supported the treatment delivery. HT was the research assessor. L-MY and HS were the trial statisticians. DF, ET, HS, and L-MY had full access to all the data in the study and take responsibility for the integrity of the data and the accuracy of the data analysis. ET, EF, and HT verified the data. All authors commented on the paper.

## Data sharing statement

De-identified participant data will be available in anonymised form from the corresponding author (DF) on reasonable request (including a study outline), subject to review and contract with the University of Oxford, following the publication of results. The trial protocol, data analytic plan, and the full statistical report are available in the Appendix.

## Declaration of interests

No commercial company was involved in this project. DF has options in XR Health, a VR for mental health company, which has no involvement in this project. AJP reports: grants from the Wellcome Trust, Innovate UK, MRC, NIHR, European Commission, Bill and Melinda Gates Foundation, Ellison Institute of Technology, Serum Institute of India, AstraZeneca; being a contributor to intellectual property licensed by Oxford University Innovation to AstraZeneca; consulting fees from the Ellison Institute of Technology; chairing the UK Department of Health and Social Care’s Joint Committee on Vaccination and Immunisation, membership of WHO’s Product Development Advisory Committee, and membership of WHO’s Salmonella Technology Advisory Group; and receipt from Moderna of mRNA for research. All other authors have no competing interests to declare.

## Appendix A Supplementary data

Needle Cognitions Questionnaire

Tactile Sensations Questionnaire

Usability ratings in the proof

Needle Fear Protocol V1.4 17.02.25

Needles SAP_v1.0_28Nov2025

ST104-A_NEEDLES SAR_v0.4_clean
