# Supplementary material for: Automated virtual reality therapy to treat needle fears (trypanophobia) in adolescents in England: a proof-of-concept cohort study and a Phase II randomised controlled trial
Source: eClinicalMedicine. 2026 Jul 15;97:104038. doi: 10.1016/j.eclinm.2026.104038 (PMC13420612; doi:10.1016/j.eclinm.2026.104038)
Supplement: Needle Cognitions Questionnaire [file mmc1.pdf]

Date:	D D / M M / Y Y Y Y
Researcher:	
Participant ID:	

Beliefs about Needles

This questionnaire lists fears that people can have about needle procedures (such as injections and blood tests). Please indicate how strongly you hold each of the following beliefs in relation to needle procedures by **circling a number from 0 (Do not believe it) – 4 (Believe it totally).**

	Do not believe it	Believe it slightly	Believe it moderately	Believe it very much	Believe it totally
1. The needle will be painful.	0	1	2	3	4
2. I will be in pain from multiple attempts to insert the needle.	0	1	2	3	4
3. It will be painful because they won't be able to find my vein.	0	1	2	3	4
4. The needle procedure will go wrong.	0	1	2	3	4
5. I will become ill.	0	1	2	3	4
6. The needle will snap in my arm.	0	1	2	3	4
7. I will bleed out.	0	1	2	3	4
8. I will come into contact with someone else's blood.	0	1	2	3	4
9. I will hurt myself with the needle.	0	1	2	3	4
10. I will be overwhelmed with fear/anxiety.	0	1	2	3	4
11. I will cry.	0	1	2	3	4
12. It will be embarrassing.	0	1	2	3	4
13. The physical symptoms of anxiety will be unbearable.	0	1	2	3	4
14. I will feel sick/ill.	0	1	2	3	4

Date:	D	D	/	M	M	/	Y Y Y Y		
Researcher:									
Participant ID:									

	Do not believe it	Believe it slightly	Believe it moderately	Believe it very much	Believe it totally
15. Seeing blood taken out of my body will be disgusting.	0	1	2	3	4
16. I won't be able to cope with the needle procedure.	0	1	2	3	4
