# Supplementary material for: Automated virtual reality therapy to treat needle fears (trypanophobia) in adolescents in England: a proof-of-concept cohort study and a Phase II randomised controlled trial
Source: eClinicalMedicine. 2026 Jul 15;97:104038. doi: 10.1016/j.eclinm.2026.104038 (PMC13420612; doi:10.1016/j.eclinm.2026.104038)
Supplement: Tactile Sensations Questionnaire [file mmc2.pdf]

Date:	DD / MM / YYYY
Researcher:	
Participant ID:	

Tactile Sensations

Some people don't like the feeling of things touching their body during needle procedures. Please indicate how bothered you would be about each situation listed.

If you needed a needle procedure, how bothered would you be about:	Not at all	A little bit	Somewhat	Very	Extremely
1. The feeling of a needle in your body.	0	1	2	3	4
2. Your skin/veins being touched.	0	1	2	3	4
3. The tightness of a tourniquet (a band that goes around your arm).	0	1	2	3	4
4. The cold/wet feeling of an alcohol wipe.	0	1	2	3	4
5. The cold/wet feeling of numbing spray/cream.	0	1	2	3	4
