# Supplementary material for: Automated virtual reality therapy to treat needle fears (trypanophobia) in adolescents in England: a proof-of-concept cohort study and a Phase II randomised controlled trial
Source: eClinicalMedicine. 2026 Jul 15;97:104038. doi: 10.1016/j.eclinm.2026.104038 (PMC13420612; doi:10.1016/j.eclinm.2026.104038)
Supplement: Usability ratings in the proof [file mmc3.pdf]

Additional data to the SAR

Usability ratings in the proof-of-concept testing

	Very easy<br>n	Easy<br>n	Difficult<br>n	Very difficult<br>n
Knowing what to do in any given VR situation was…	5	6	0	0
Understanding the coach's instructions was…	6	5	0	0
Carrying out an action was…	4	5	2	0
Moving through the program was…	4	6	0	1
Learning what to do and how to do it was…	6	5	0	0
Remember how to do things a second time was…	7	4	0	0
	Agree<br>n	Agree a little<br>n	Disagree a little<br>n	Disagree<br>n
When using the VR, I felt like I was in the situation.	3	8	0	0
I was satisfied with the overall VR experience.	8	3	0	0
I enjoyed using the treatment.	8	3	0	0
The VR made me feel sick.	0	1	0	10
