# Supplementary material for: Automated virtual reality therapy to treat needle fears (trypanophobia) in adolescents in England: a proof-of-concept cohort study and a Phase II randomised controlled trial
Source: eClinicalMedicine. 2026 Jul 15;97:104038. doi: 10.1016/j.eclinm.2026.104038 (PMC13420612; doi:10.1016/j.eclinm.2026.104038)
Supplement: Needle Fear Protocol V1.4 17.02.25 [file mmc4.pdf]

# Trial Title: Virtual Reality (VR) for Needle Fears: A Cohort Study and a Randomised Controlled Trial of an Automated VR Therapy for the Treatment of Needle Fears (trypanophobia)

**Internal Reference Number / Short title:** VR for Needle Fears

**Ethics Ref:** 23/SC/0420

**IRAS Project ID: 334022**

**Date and Version No**: 17.02.2025, Version 1.4


| | |
|---|---|
| **Chief Investigator:** | Professor Daniel Freeman, University of Oxford and Oxford Health NHS Foundation Trust |
| **Investigators:** | Dr Eve Twivy, University of Oxford, Oxford Health NHS Foundation Trust, and Oxford University Hospitals NHS Foundation Trust<br>Jason Freeman, University of Oxford<br>Prof Ly-Mee Yu, University of Oxford<br>Dr Aitor Rovira, University of Oxford and Oxford Health NHS Foundation Trust<br>Andre Lages Miguel, University of Oxford<br>Rupert Ward, University of Oxford and Oxford Health NHS Foundation Trust<br>Matthew Bousfield, University of Oxford<br>Dr Felicity Waite, University of Oxford and Oxford Health NHS Foundation Trust<br>Prof Helen McShane, University of Oxford and Oxford University Hospitals NHS Foundation Trust<br>Prof Andrew J. Pollard, University of Oxford and Oxford University Hospitals NHS Foundation Trust |



| | |
|---|---|
| **Sponsor:** | University of Oxford |
| **Funder:** | Beryl Alexander Charity and NIHR Oxford Health Biomedical Research Centre |


**Chief Investigator Signature:**


© Copyright: The University of Oxford and Oxford University Hospitals NHS Foundation Trust 2019

**Statistician Signature:**

**Potential conflicts of interest:** None.

**Confidentiality Statement**

This document contains confidential information that must not be disclosed to anyone other than the Sponsor, the Investigator Team, HRA, host organisation, and members of the Research Ethics Committee and Regulatory Authorities unless authorised to do so.


© Copyright: The University of Oxford and Oxford University Hospitals NHS Foundation Trust 2019

## TABLE OF CONTENTS

1. KEY TRIAL CONTACTS .... 6
2. LAY SUMMARY .... 7
3. SYNOPSIS .... 8
4. ABBREVIATIONS .... 11
5. BACKGROUND AND RATIONALE .... 12
6. AIM AND OBJECTIVES .... 13
7. TRIAL DESIGN .... 13
8. PARTICIPANT IDENTIFICATION .... 16
8.1. Trial Participants .... 16
8.2. Inclusion Criteria .... 16
8.3. Exclusion Criteria .... 16
9. TRIAL PROCEDURES .... 16
9.1. Recruitment .... 16
9.2. Screening and Eligibility Assessment .... 17
9.3. Informed Consent .... 17
9.4. Randomisation .... 18
9.5. Blinding and code-breaking .... 18
9.6. Baseline Assessments .... 18
9.7. Subsequent Visits .... 19
9.8. Sample Handling .... 20
9.9. Early Discontinuation/Withdrawal of Participants .... 20
9.10. Definition of End of Trial .... 20
10. TRIAL INTERVENTIONS .... 20
10.1. Investigational Medicinal Product(s) (IMP) Description .... 21
10.2. Other Treatments (non-IMPS) .... 21
10.3. Other Interventions .... 23
11. SAFETY REPORTING .... 23
11.1. Adverse Event Definitions .... 23
11.2. Assessment results outside of normal parameters as AEs and SAEs .... 24
11.3. Assessment of Causality .... 24
11.4. Expectedness .... 25
11.5. Procedures for Reporting Adverse Events .... 25
11.6. Reporting Procedures for Serious Adverse Events .... 25
11.6.1. Events exempt from immediate reporting as SAEs .... 25


© Copyright: The University of Oxford and Oxford University Hospitals NHS Foundation Trust 2019

11.7. Medicines and Healthcare Products Regulatory Agency (MHRA) Reporting .......................... 25
12. STATISTICS .......................................................................................................................... 26
12.1. Statistical Analysis Plan (SAP) ......................................................................................... 26
12.2. Description of Statistical Methods ................................................................................... 26
12.3. Sample Size Determination ............................................................................................. 27
12.4. Analysis Populations ........................................................................................................ 27
12.5. Decision Points ................................................................................................................ 28
12.6. Stopping Rules ................................................................................................................. 28
13. DATA MANAGEMENT ........................................................................................................ 28
13.1. Source Data ..................................................................................................................... 28
13.2. Access to Data .................................................................................................................. 28
13.3. Data Recording and Record Keeping ................................................................................ 28
14. QUALITY ASSURANCE PROCEDURES ................................................................................. 29
14.1. Risk assessment ............................................................................................................... 29
14.2. Monitoring ....................................................................................................................... 29
14.3. Trial committees .............................................................................................................. 29
15. PROTOCOL DEVIATIONS .................................................................................................... 29
16. SERIOUS BREACHES ........................................................................................................... 30
17. ETHICAL AND REGULATORY CONSIDERATIONS ................................................................. 30
17.1. Declaration of Helsinki ..................................................................................................... 30
17.2. Guidelines for Good Clinical Practice ............................................................................... 30
17.3. Approvals ......................................................................................................................... 30
17.4. Other Ethical Considerations ............................................................................................ 30
17.5. Reporting ......................................................................................................................... 31
17.6. Transparency in Research ................................................................................................ 31
17.7. Participant Confidentiality ................................................................................................ 31
17.8. Expenses and Benefits ...................................................................................................... 31
18. FINANCE AND INSURANCE ................................................................................................. 31
18.1. Funding ............................................................................................................................ 31
18.2. Insurance ......................................................................................................................... 32
18.3. Contractual arrangements ................................................................................................ 32
19. PUBLICATION POLICY ......................................................................................................... 32
20. DEVELOPMENT OF A NEW PRODUCT/ PROCESS OR THE GENERATION OF INTELLECTUAL PROPERTY (IP) 32
21. ARCHIVING .......................................................................................................................... 32


© Copyright: The University of Oxford and Oxford University Hospitals NHS Foundation Trust 2019

22. REFERENCES .......... 32
23. APPENDIX A: SCHEDULE OF PROCEDURES .......... 35
24. APPENDIX B: AMENDMENT HISTORY .......... 38


© Copyright: The University of Oxford and Oxford University Hospitals NHS Foundation Trust 2019

# 1. KEY TRIAL CONTACTS

| | |
|---|---|
| **Chief Investigator** | Professor Daniel Freeman, Department of Experimental Psychology, New Radcliffe House, Radcliffe Observatory Quarter, Woodstock Road, Oxford OX2 6GG. Tel. No. 01865 613109. Email: Daniel.Freeman@psy.ox.ac.uk |
| **Trial co-ordinator** | Dr Eve Twivy, Department of Experimental Psychology, New Radcliffe House, Radcliffe Observatory Quarter, Woodstock Road, Oxford OX2 6GG. Tel. No. 01865 618261. Email: Eve.Twivy@psy.ox.ac.uk |
| **Sponsor** | University of Oxford Research Governance, Ethics & Assurance, Joint Research Office, Boundary Brook House, Churchill Drive, Headington, Oxford, OX3 7GB Email: RGEA.Sponsor@admin.ox.ac.uk. Tel 01865 616480. |
| **Funder(s)** | Beryl Alexander Charity<br><br>NIHR Oxford Health Biomedical Research Centre |
| **Clinical Trials Unit** | Primary Care Clinical Trials Unit, Nuffield Department of Primary Care, University of Oxford, Radcliffe Observatory Quarter, Woodstock Road, Oxford. OX2 6GG. Tel. No. 0207 7848 0532 Email: ctu@kcl.ac.uk Website: https://ctu.co.uk/ |
| **Statisticians** | Professor Ly-Mee Yu and Dr Victoria Harris, Nuffield Department of Primary Care, University of Oxford, Radcliffe Observatory Quarter, Woodstock Road, Oxford. OX2 6GG Email: primarycarectu@phc.ox.ac.uk. |
| **Trial Management Committee** | This will be setup prior to commencement of the Randomised Controlled Trial. |


© Copyright: The University of Oxford and Oxford University Hospitals NHS Foundation Trust 2019

## 2. LAY SUMMARY

**Background:**

The hypodermic needle - used to inject substances (e.g., saline, medications, vaccinations) or extract fluids (e.g. blood) in clinical procedures – may be the most important medical device invented. Billions of needles are used worldwide each year. However, a significant minority of the population are very fearful of needles. This can make medical procedures unpleasant. It can also lead to avoidance of vaccination, blood donation and tests, and uptake of treatments. Fear of needles is especially high in children and adolescents. Needle fear can be successfully treated using psychological therapy (graded exposure and applied tension) but because of a shortage of therapists very few people are able to access such help.

**Aim:**

Working with adolescents with needle fears, we have automated the delivery of evidence-based psychological therapy for needle fear within virtual reality (VR). A virtual coach guides users through the therapy. As such, the therapy can be supported by a range of professionals, for example, nurses or school pastoral staff, which can dramatically increase the potential for scalability. We now wish to assess the effectiveness for young people (ages 12-16 years) of this automated VR therapy for needle fear.

**Methods:**

The project has two elements:

**1. Initial clinical testing.** 12 young people (ages 12-16 years) with needle fear will all receive the VR therapy. They will be assessed before and after completing the intervention. If there is evidence of improvement in the needle fear then we will proceed to the full clinical trial.

**2. Full clinical trial.** The VR therapy for needle fear will be tested in a randomised controlled trial. 60 young people reporting significant needle fears will be randomised to receive the VR therapy or the control group (no treatment). Assessments will be conducted at baseline, end of therapy (three weeks), and at follow-up (six weeks). We will offer the VR therapy to young people in the control group after their participation.

**Patient involvement:**

A group of young people with lived experience of needle fears helped advise on the development of the VR therapy.

**Funding:**

The research is funded by the Beryl Alexander charity and the NIHR Oxford Health Biomedical Research Centre.


© Copyright: The University of Oxford and Oxford University Hospitals NHS Foundation Trust 2019

## 3. SYNOPSIS

| | | | |
|---|---|---|---|
| Trial Title | VR for Needle Fears | | |
| Trial registration | Following ethical approval, and before the trial starts, an ISRCTN will be obtained. | | |
| Sponsor | University of Oxford | | |
| Funder | Beryl Alexander charity and NIHR Oxford Health Biomedical Research Centre | | |
| Clinical Phase | I and II | | |
| Trial Design | Phase I (proof of concept): Cohort test<br>Phase II: Randomised controlled | | |
| Trial Participants | Adolescents (aged 12-16 years old) with needle fears | | |
| Sample Size | Phase I: 12<br>Phase II: 60 | | |
| Planned Trial Period | Phase I: Individual participant involvement over approximately two to three weeks. 1st February 2024-1st April 2024.<br>Phase II: Individual participant involvement over approximately six to eight weeks. 28th October 2024-31st December 2025. | | |
| Planned Recruitment period | Phase I: 1st February 2024-15th March 2024<br>Phase II: 4th October 2024- 15th July 2025. | | |
| | Objectives | Outcome Measures | Timepoint(s) |
| Primary | Phase I: To test whether the VR therapy is associated with a reduction in needle fear.<br><br>Phase II: To test whether the VR therapy reduces needle fear. | Phase I: The Injection Phobia Scale- Anxiety (Child Version: Oar et al., 2017; Ost et al., 1992)<br><br>Phase II: The Injection Phobia Scale- Anxiety (Child Version: Oar et al., 2017; Ost et al., 1992) | Before (0 weeks) and after treatment (2 weeks).<br><br>0 and 3 weeks (end of treatment, primary outcome point) |
| Secondary | Phase 1:<br>1. To test whether the VR therapy has high usability ratings, high satisfaction ratings, and few side effects. | Phase I:<br>1. Usability Questionnaire (Modified wording: Lambe et al., 2020), Child Treatment Satisfaction (Modified wording: Ollendick et al., 2015), and modified Oxford – VR Side | Phase I:<br>1. After treatment (2 weeks). |


© Copyright: The University of Oxford and Oxford University Hospitals NHS Foundation Trust 2019

| | | | |
|---|---|---|---|
| | | Effects Scale (Freeman et al., 2023). | |
| | 2. To test whether the VR therapy is associated with reduction in anxiety and avoidance of needle-related stimuli. | 2. Behavioural Avoidance Task (BAT) | 2. Before (0 weeks) and after treatment (2 weeks). |
| | Phase II: | Phase II: | Phase II: |
| | 1. To test whether the reduction in needle fear persists. | 1. The Injection Phobia Scale- Anxiety (Child Version: Oar et al., 2017; Ost et al., 1992) | 1. 6 weeks |
| | 2. To test whether the VR therapy has high satisfaction ratings. | 2. Child Treatment Satisfaction (Modified wording: Ollendick et al., 2015) | 2. After treatment session (i.e., directly at the end of the treatment session) |
| | 3. To test whether the VR therapy is associated with reduction in fearful cognitions and disgust reactions. | 3. Needle Cognitions Questionnaire, Disgust Emotion Scale for Children – Injections and Blood Draws Subscale (Muris et al., 2012) | 3. 0,3,6 weeks |
| | 4. To test whether changes in needle-related fearful cognitions and disgust reactions mediate change in needle fear. | 4. Needle Cognitions Questionnaire, Disgust Emotion Scale for Children – Injections and Blood Draws Subscale (Muris et al., 2012) | 4. 0,3,6 weeks |
| | 5. To test whether there are moderators of the VR therapy. | 5. The moderators tested will be age, gender, ethnicity, a history of fainting, and a modified specific phobia subsection of Anxiety Disorder Interview Schedule-Child Version (Silverman & | 5. 0 weeks |


© Copyright: The University of Oxford and Oxford University Hospitals NHS Foundation Trust 2019

| | | | |
|---|---|---|---|
| | 6. To explore side effects, level of fear and vasovagal symptoms after using the VR therapy. | Albano, 1996). Aversion to tactile sensations will also be measured using the Needle Procedure Tactile Sensations Questionnaire as an exploratory moderator.<br><br>6. Modified Oxford – VR Side Effects Scale (Freeman et al., 2023), VAS scale – Fear, 4-item Blood Donation Reaction Inventory (France et al., 2008). | 6. After treatment session (i.e., directly at the end of the treatment session) |
| Intervention<br>• Other intervention(s) | UKCA marked VR therapy<br>Delivery supported by a research team member.<br>Hardware: Meta Quest headset. | | |
| Comparator | Treatment as usual, which is typically no treatment. | | |


© Copyright: The University of Oxford and Oxford University Hospitals NHS Foundation Trust 2019

## 4. ABBREVIATIONS

| | |
|---|---|
| AE | Adverse event |
| AR | Adverse reaction |
| CI | Chief Investigator |
| CRF | Case Report Form |
| CT | Clinical Trials |
| DMC/DMSC | Data Monitoring Committee / Data Monitoring and Safety Committee |
| GCP | Good Clinical Practice |
| GP | General Practitioner |
| HEAP | Health Economic Analysis Plan |
| HRA | Health Research Authority |
| IB | Investigators Brochure |
| ICF | Informed Consent Form |
| ICH | International Conference on Harmonisation |
| LEAP | Lived Experience Advisory Group |
| MHRA | Medicines and Healthcare products Regulatory Agency |
| NHS | National Health Service |
| OHFT | Oxford Health Foundation Trust |
| PI | Principal Investigator |
| PIL | Participant/ Patient Information Leaflet |
| PIS | Patient Information Sheet |
| PPI | Patient and Public Involvement |
| R&D | NHS Trust R&D Department |
| RCT | Randomised controlled trial |
| REC | Research Ethics Committee |
| RES | Research Ethics Service |
| RGEA | Research Governance Ethics & Assurance |
| RSI | Reference Safety Information |
| SAE | Serious Adverse Event |
| SAP | Statistical Analysis Plan |
| SAR | Serious Adverse Reaction |
| SDV | Source Data Verification |
| SOP | Standard Operating Procedure |


© Copyright: The University of Oxford and Oxford University Hospitals NHS Foundation Trust 2019

| | |
|---|---|
| SUSAR | Suspected Unexpected Serious Adverse Reactions |
| TAU | Treatment as Usual |
| TMF | Trial Master File |
| Virtual reality | VR |

## 5. BACKGROUND AND RATIONALE

In 2021 we conducted an epidemiological survey of injection fear in 15,014 UK adults, quota sampled to match the population for age, gender, ethnicity, income, and region (Freeman et al, 2023). One in four of the population screened positive for blood-injection-injury phobia. For example, the fear of receiving a hypodermic injection in the arm was rated as intense by 3.6%, considerable by 8.3%, and mild by 31.3% of the population. Fears were significantly higher in younger people. For example, in adults under the age of 30, the fear of receiving a hypodermic injection in the arm was rated as intense by 9.3%, considerable by 19.0%, and mild by 38.4%. This is consistent with previous research in which needle fears have been found to be at their highest in children and adolescents (Taddio et al, 2012; McLenon, & Rogers, 2019). We conducted our UK survey to test the potential contribution of needle fear to COVID-19 vaccination hesitancy. The population attributable fraction (PAF) indicated that if blood-injection-injury phobia were absent then this may prevent 11.5% of all instances of COVID-19 vaccine hesitancy. It is an illustration of the impact of needle fear.

For people with needle fears, needles have become associated with threat. People fear, for example, that the procedure will go wrong (e.g. the needle may snap, given wrong injection), or it will be very painful, or that they will have a panic attack or be unable to cope. Uniquely among anxiety disorders, part of a common response pattern for needle phobia is a drop in heart rate and blood pressure leading to fainting. People therefore also worry about fainting and its consequences. There is also evidence that disgust reactions may be a further contributory factor to needle, blood, and injury fears (Lumley & Melamed, 1992; Olatunji et al, 2006). Needle fear is treatable with psychological therapy. Exposure therapy (graded presentation of needle-related stimuli) and applied tension (learning to raise blood pressure when early signs of a drop in blood pressure are noticed) produce large reductions in blood-injection-injury fears (Ayala et al, 2009; McMurty et al, 2016; Hiermeier & Mofrad, 2020). However there is highly limited availability of such treatment.

Virtual reality (VR) may provide an approach to increasing the availability of effective treatment for needle fears. There are three key reasons. First, delivery of therapy can be automated within VR meaning that a therapist is not required. Second, VR can be a therapeutic medium since people find it easier to approach feared stimuli in VR since they know that it is not real but the learning made still transfers to the real-world. Finally, there is a potential in VR to present stimuli in ways that are therapeutic but impossible in the real world meaning that it could increase treatment efficacy. VR has already shown promise for treating needle fear. A randomised controlled trial with 43 adults with blood-injection-injury phobia


© Copyright: The University of Oxford and Oxford University Hospitals NHS Foundation Trust 2019

showed that a 90-minute therapist-guided VR intervention reduced fear of injections (Jiang et al, 2020). We have previously developed several successful automated VR therapies (e.g. Freeman et al, 2018; Freeman et al, 2022). With young people with needle fears, we have now developed an automated VR therapy for needle fears. We have focussed on younger ages since this is when needle fears are most prevalent, there are several vaccinations to be given (e.g., HPV, Td/IPV, and MenACWY are typically given in year 8 and year 9), and there is likely value in earlier experiences of positive healthcare provision. The VR therapy if successful can also be easily adapted for older age groups.

## 6. AIM AND OBJECTIVES

**Aim**

The planned research will evaluate the effectiveness of the automated VR therapy for needle fears in adolescents (aged 12-16).

**Objectives**

Phase I:

1. To test whether the VR therapy is associated with a reduction in needle fear.
2. To test whether the VR therapy has high usability ratings, high satisfaction ratings, and few side effects.
3. To test whether the VR therapy is associated with reduction in anxiety and avoidance of needle-related stimuli.

Phase 2:

1. To test whether the VR therapy reduces needle fear.
2. To test whether the reduction in needle fear persists.
3. To test whether the VR therapy has high satisfaction ratings.
4. To test whether the VR therapy is associated with reduction in fearful cognitions and disgust reactions.
5. To test whether changes in needle-related fearful cognitions and disgust reactions mediate change in needle fear (i.e., testing how the VR therapy may work).
6. To test whether there are moderators (age, ethnicity, gender, history of fainting, or phobia diagnosis) of the VR therapy (i.e., testing whether some people particularly benefit). Aversion to tactile sensations will also be tested as an exploratory moderator.
7. To explore side effects, level of fear and vasovagal symptoms after using the VR therapy.

## 7. TRIAL DESIGN


© Copyright: The University of Oxford and Oxford University Hospitals NHS Foundation Trust 2019

The University of Oxford and Oxford Health NHS Foundation Trust (OHFT) are the research sites. Recruitment will be via the Oxfordshire School Aged Immunisation Service (SAIS), Berkshire School Aged Immunisation Team, Buckinghamshire School Aged Immunisation Team, School Health Nurses and general advertisements (**e.g.,** via radio, social media and posters in schools/community venues).

Phase I (proof of concept): A pre-post cohort design. Participants will be assessed before and after receiving the VR therapy. Level of reduction in needle fear, therapy satisfaction, usability, and side effects will be assessed. The assessments involve the participant completing questionnaires relevant to assessing their needle fear and experience of the VR therapy. Individual participant involvement will be over approximately two to three weeks.

Phase II: A randomised controlled trial testing the VR therapy against no intervention. Moderation and mediation tests are built into the trial design. Assessments will be conducted at 0, 3, and 6 weeks by a research assistant blind to group allocation. The assessments involve the participant completing questionnaires relevant to assessing their needle fear.

*Primary RCT question (PICO question)*: For young people with needle fear, can an automated VR therapy, compared to no treatment, reduce needle fear? The primary time-point is 3 weeks (end of treatment).

*Secondary RCT questions*:

*Outcomes*

1. Compared to no treatment, does the VR therapy lead to reduction in anxiety and avoidance of needle-related stimuli and fearful cognitions and disgust reactions?

2. Are VR therapy benefits maintained at later follow-up?

3. How satisfied are people treated with the VR therapy?

4. Does the VR therapy lead to side effects, fear response and vasovagal symptoms?

*Moderation*

1. Is the primary VR therapy outcomes moderated by age, ethnicity, gender, history of fainting, or phobia diagnosis?

*Mediation*

1. Do changes in threat cognitions about needles and disgust mediate change in needle fear with VR therapy?


© Copyright: The University of Oxford and Oxford University Hospitals NHS Foundation Trust 2019

Figure 1. Phase II: Trial flow diagram

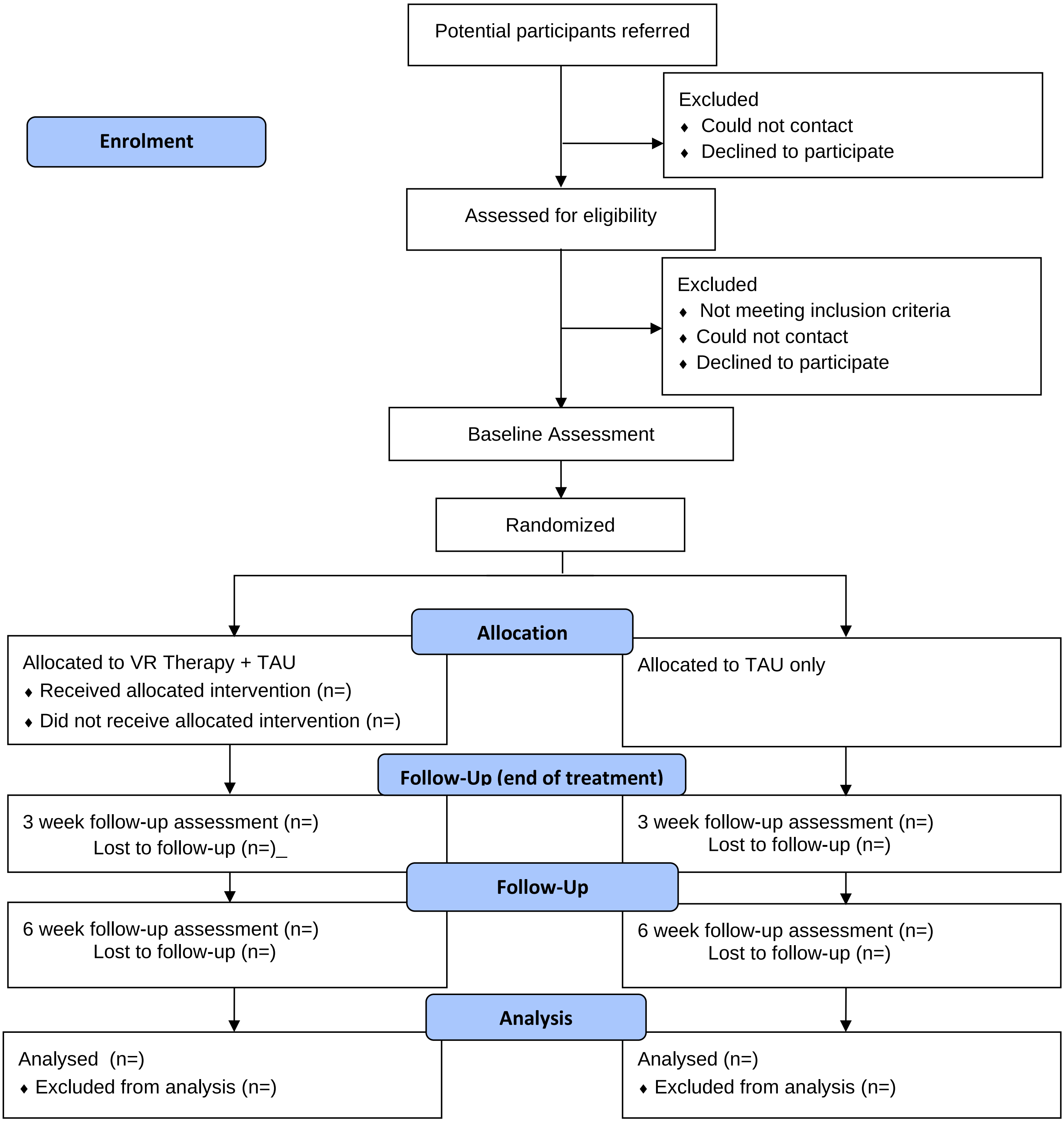



© Copyright: The University of Oxford and Oxford University Hospitals NHS Foundation Trust 2019

# 8. PARTICIPANT IDENTIFICATION

## 8.1. Trial Participants

Participants are adolescents (aged 12-16 years old) with a needle fear. This is identical for both phases of the research.

The principal route of recruitment will be via the Oxford Health NHS Foundation Trust Oxfordshire School Aged Immunisation Service (SAIS). We will also recruit via schools and advertisement (e.g., radio, social media adverts and posters).

## 8.2. Inclusion Criteria

All participants:

- Aged 12-16 years old (up to $16^{th}$ birthday).
- Have significant needle fears that they would like treated (as determined by a screening tool).
- Willing and able to give assent for participation in the study.
- A parent/guardian is willing and able to give informed consent for their child’s participation in the study.

## 8.3. Exclusion Criteria

The participant may not enter the trial if ANY of the following apply:

- Photosensitive epilepsy or significant visual, auditory, or balance impairment that would make use of VR inappropriate.
- Current engagement in any other psychological treatment for needle fear.
- Command of English inadequate for engaging in the therapy or completing the assessments.
- A participant may also not enter the trial if there is another factor, which, in the judgement of the investigator, would preclude the provision of informed consent/assent or from safely engaging with the trial procedures. Reason for exclusion will be recorded.

# 9. TRIAL PROCEDURES

The schedule of procedures is summarised in Appendix A.

## 9.1. Recruitment


© Copyright: The University of Oxford and Oxford University Hospitals NHS Foundation Trust 2019

The principal method of recruitment will be via the Oxford Health Oxfordshire School Aged Immunisation Service (SAIS) sending out a letter describing the study to parents/guardians of pupils who are due to be vaccinated. A description of the study may also be included in other communication (e.g., letters/emails/leaflets/website) from the SAIS to parents/guardians and pupils. Pupils and parents/guardians will need to then contact the study team directly if they are interested in the research. Nurses/clinicians working in the SAIS and School Health Nurses can also directly refer pupils (that they may have identified via screening their caseload) to the study team after obtaining verbal or written consent from parents/guardians to share their and their child's contact details with the research team (i.e., the nurse/clinician would ask the parent/guardian as part of a verbal or written communication if they would be happy for their contact details to be shared with a research team at the University of Oxford in order to find out more about a research study which is exploring whether a new virtual reality therapy can help young people overcome a fear of needles). If the parent/guardian indicates they are happy for their contact details to be shared with the research team (responding affirmatively to this as part of a verbal conversation or via text/email), their contact details would be shared by the nurse/clinician with the research team via a phone call or sent securely from an OHFT email address to an OHFT email address. We will also advertise the research (e.g., local radio/social media/posters in schools/community venues). All suitable people will be given at least 24 hours to consider taking part in the trial.

Berkshire Healthcare NHS Foundation trust and Buckinghamshire Healthcare NHS Trust will be included as Participant Identification Centres when recruiting for phase II (RCT). The Berkshire School Aged Immunisation Team (part of Berkshire Healthcare NHS Foundation Trust) and Buckinghamshire School Aged Immunisation Team (part of Buckinghamshire Healthcare NHS Trust) would be involved in identifying potential research participants who may meet the trial criteria and directing them/their parent/guardian to our research team, as well as more broadly advertising the trial to pupils and parents/guardians within their service (e.g., through letters/emails/leaflets/website/social media).

### 9.2. Screening and Eligibility Assessment

The eligibility assessment will be conducted by a research team member, most commonly a clinical psychologist or research assistant. This will typically be done remotely (i.e., online/telephone/video call). Participants must satisfy all the inclusion and exclusion criteria listed in section 8. A brief screening tool developed by the research team will be used to determine if a potential participant has a significant needle fear that they would like treated. The baseline assessment must commence within 4 weeks of the eligibility assessment. If it is after this period, a brief re-screening will be conducted to confirm eligibility.

### 9.3. Informed Consent

Written informed consent will be obtained by the research team before any study procedures are performed. The research team member obtaining consent will be suitably qualified and experienced, and have been authorised to do so by the Chief Investigator. Given that all participants are under 16, consent will be obtained from the parent/guardian. This will be after provision of a Participant Information Sheet (PIS) to the parent/guardian, opportunities to ask questions, and at least 24 hours to decide.

Written – including electronic and, if needed, verbal - versions of the PIS and Informed Consent Form (ICF) will be presented to the parents/guardians of participants by the research team (or OHFT staff in the case of giving the PIS only) detailing no less than: the exact nature of the study; what it will involve for the participant; the implications and constraints of the protocol; the known side effects and any risks involved


© Copyright: The University of Oxford and Oxford University Hospitals NHS Foundation Trust 2019

in taking part. It will be clearly stated that the participant is free to withdraw from the study at any time for any reason without prejudice to future care, and with no obligation to give the reason for withdrawal.

The parent/guardian of the participant will be allowed as much time as wished to consider the information, and the opportunity to question the Investigator, or other independent parties to decide whether they will participate in the study. Written informed consent will then be obtained by means of a participant's parent/guardian dated signature and dated signature of the person who presented and obtained the ICF. A copy of the signed ICF will be given to the participant's parent/guardian. The original signed ICF will be retained at the study site. We will also accept written informed consent electronically. Oral consent can be taken over the phone using the remote consent form, which will be signed and dated by the person who obtained the informed consent verbally. If collecting consent orally or electronically, the Consent Form will be sent to the participant's parent/guardian via secure post or password-protected email.

Assent from young people will also be sought using the same process as described above, but utilising an adolescent version of the PIS and Assent Form.

Informed consent/assent will typically be done in person at the University of Oxford (or at the participant's home/school/community venue where appropriate, according to the University of Oxford's safety policy), however this can be done remotely as described above.

### 9.4. Randomisation

All participants in the cohort study will receive the intervention and there is no randomisation.

Participants in the randomised controlled trial will be randomised once they have completed the baseline assessment. Participants will be allocated to one of the trial arms using a 1:1 allocation ratio. Randomisation will be carried out by a validated online system provided by Sealed Envelope (www.sealedenvelope.com). Randomisation will use a permuted blocks algorithm, with randomly varying block size.

We will offer the VR therapy to participants in the control arm after completion of the 6-week follow-up assessment.

### 9.5. Blinding and code-breaking

There is no blinding in the cohort study.

In the RCT, the research assessors will be blind to group allocation, but the participants and staff member present will not be (they cannot be blinded to whether a psychological intervention is delivered or not).

### 9.6. Baseline Assessments

Assessments are conducted by a research team member (typically a Clinical Psychologist or Research Assistant). They are typically in person at the University of Oxford (or at the participant's home/school/community venue where appropriate), but can be done remotely (e.g., via video call/phone call/email/post). Basic demographic information will be collected for all participants (age, gender, ethnicity). The Specific Phobia subsection of the Anxiety Disorder Interview Schedule (Child Version: Silverman & Albano, 1996) will be used to determine whether participants meet diagnostic criteria for


© Copyright: The University of Oxford and Oxford University Hospitals NHS Foundation Trust 2019

blood-injection-injury phobia and have a history of fainting. The Needle Procedure Tactile Sensations Questionnaire will be used to assess aversion to tactile sensations.

*Phase I Cohort study:*

Primary measure: The Injection Phobia Scale- Anxiety (Child Version: Oar et al., 2017; Ost et al., 1992)

Secondary measure: Behavioural Avoidance Task (BAT). Participants would be asked if they can try a task involving real needles. If they agree to this, it might involve them looking at needles, holding needles, and using needles on objects. However, the participants will not have to do anything that they don't want to do, and participants would not undergo any real-life medical procedures.

*Phase II Randomised controlled trial:*

Primary: The Injection Phobia Scale- Anxiety (Child Version)

Secondary outcomes:

- Disgust Emotion Scale for Children – Injections and Blood Draws Subscale (Muris et al., 2012)
- Needle Cognitions Questionnaire

Moderators:

- Age, gender, ethnicity, history of fainting, and diagnosis of blood-injection-injury phobia.
- Needle Procedure Tactile Sensations Questionnaire

Mediators:

- Disgust Emotion Scale for Children – Injections and Blood Draws Subscale
- Needle Cognitions Questionnaire

### 9.7. Subsequent Visits

Subsequent assessments are typically in person at the University of Oxford (or at the participant's home/school/community venue where appropriate, according to the University of Oxford's safety policy). The assessment at 3 weeks and 6 weeks can be done remotely (e.g., via video call/phone call/email/post).

In the cohort study, participants will repeat after treatment (at 2 weeks) the baseline assessment measures and also complete the modified Child Treatment Satisfaction (Ollendick et al., 2015), modified Usability Questionnaire (Lambe et al., 2020), and modified Oxford-VR Side Effects Scale (Freeman et al., 2023). The treatment deliverer will also administer the Fear Visual Analogue Scale and the 4-item Blood Donations Reaction Inventory (France et al., 2008) at the end of the VR therapy session.

In the RCT, participants will repeat the baseline outcome measures at 3 and 6 weeks. Mediators will be repeated at 3 and 6 weeks. Moderators will not be repeated. Satisfaction with the VR therapy and side effects will be assessed using the Child Treatment Satisfaction and Oxford-VR Side Effects Scale, administered via a link (or paper copy) provided by the treatment deliverer. Fear experienced during the VR therapy and vasovagal symptoms will also be assessed by the treatment deliverer using the Fear Visual Analogue Scale and the Blood Donations Reaction Inventory.


© Copyright: The University of Oxford and Oxford University Hospitals NHS Foundation Trust 2019

### 9.8. Sample Handling

No samples will be taken.

### 9.9. Early Discontinuation/Withdrawal of Participants

During the course of each study a participant or their parent/guardian may choose to withdraw early from the study treatment at any time. This may happen for several reasons, including but not limited to:

- The occurrence of what the participant or their parent/guardian perceives as an intolerable AE.
- Inability to comply with study procedures
- Participant or their parent/guardian’s decision

Participants or their parent/guardian may choose to stop treatment and/or study assessments. Participants or their parent/guardian may also withdraw their assent/consent, meaning that they wish to withdraw from the study completely. Withdrawal from the study or study intervention will not affect participants’ usual care.

According to the design of the study, participants may have the following options for withdrawal:

1) Participants and their parent/guardian can withdraw from the study but permit data obtained up until the point of withdrawal to be retained for use in the study analysis. No further data would be collected after withdrawal.
2) Participants or their parent/guardian can withdraw completely from the study and withdraw the data collected up until the point of withdrawal up to two weeks after completing participation.

In addition, the Investigator may discontinue a participant from the intervention at any time if the Investigator considers it necessary for any reason including:

- Ineligibility (either arising during the study or retrospectively having been overlooked at screening);
- An adverse event which requires discontinuation of the intervention;
- Clinical decision.

If the participant is withdrawn due to an adverse event, the Investigator will arrange for follow-up visits or telephone calls until the adverse event has resolved or stabilised.

The reason for withdrawal will be recorded in the Case Report Form (CRF).

### 9.10. Definition of End of Trial

The end of trial is the point at which all the data has been entered and queries resolved. The anticipated end date is 31st December 2025.

## 10. TRIAL INTERVENTIONS


© Copyright: The University of Oxford and Oxford University Hospitals NHS Foundation Trust 2019

### 10.1. Investigational Medicinal Product(s) (IMP) Description

An IMP is not being tested.

### 10.2. Other Treatments (non-IMPS)

The treatment being tested is automated VR therapy for needle fears. This software is intended to reduce needle fears. It is a cognitive-behavioural exposure and applied tension intervention. The treatment content was designed by the Oxford Cognitive Approaches to Psychosis (O-CAP) research group at the University of Oxford, with young people with lived experience taking part in the design process. The treatment was programmed by the University of Oxford. The treatment will achieve UKCA marking and registration as an individual Class I medical device (standalone software as a medical device) before the start of the testing.

The VR for needle fears software application is composed of a set of virtual environments, including different scenes created using 3D models, ambient audio, and 3D computer characters, with animations and speech. The environments are driven by source code which handles the logic of the program, the behaviour of the computer characters, as well as the user interaction and data storage. The software is built using Unity (Unity Technologies©). Unity acts as a render engine, displaying the virtual environments to the user through the headset.

The application will run through the Unity software application on a Meta Quest VR Headset. All technical requirements will be as per headset requirements. Accessory hardware and software are already commercially available and have not been modified for the VR therapy.

The programme takes approximately three hours to complete. It can be done in a half day or several shorter meetings on different days. The tasks are completed while sitting down. A research staff member will be present while the programme is used. The VR treatment sessions will typically take place at the University of Oxford (or a participant's home/school/community venue where appropriate).

Key treatment components include: psychoeducation; applied tension technique to prevent fainting; graded exposure to feared situations; modelling by computer characters; encouragement and positive reinforcement.

The VR therapy is set within a school/college. There is a virtual coach (called Farah). There are five levels of exposure (looking at needles, picking up needles, using needles, observing needle procedures, and receiving needle procedures), which are summarised in the table below.

| Level | Aim | Activities |
|---|---|---|
| **1. Looking** | Require users to look at needle-related objects. Includes education about different types of needles and their purpose. | 'Treasure hunt' type activities involving looking for needles and related medical equipment:<br>• Unlocking cabinet to find (needle-related) objects.<br>• Finding specific (needle-related) objects in display case. |


© Copyright: The University of Oxford and Oxford University Hospitals NHS Foundation Trust 2019

| | | |
|---|---|---|
| **2. Picking up** | Require users to pick up and hold needles. Also includes education about different types of needles and their purpose. | Sorting colour-coded needles – user is presented with 6 realistic needles of differing sizes used for different procedures. User has to place the needles into a colour-coded holder. |
| **3. Using** | Requires the user to utilise the function of needles (i.e., piercing, extracting, injecting). Also includes an explanation of what happens during needle procedures. | There are three areas within this level for completion of three categories of activities.<br><br>Inanimate objects:<br>• Piercing air balloons with needles.<br>• Extracting liquid from a blood orange.<br>• Injecting jam into doughnuts.<br><br>Animal:<br>• Injecting a penguin<br><br>(Virtual) Humans:<br>• Finger prick on self (virtual hand).<br>• Taking blood from a giant arm. |
| **4. Observing** | Requires users to observe realistic needle procedures being performed by health professional. | • Waiting in line with other students to receive an injection. Teleporting room where you start from observing further away (e.g., in doorway) and then get closer (e.g., next in line). Background chatter to recreate school vaccination session.<br>• One main station where can visibly see nurse administering injection. Tables in background with less detail.<br>• Watching another student receive an injection close-up (whole process e.g., consent, preparation vaccine, injection, check-in afterwards). |
| **5. Receiving** | Require users to receive realistic needle procedures performed by a (virtual) health professional (on a virtual arm and hand for the user). | Will receive an intramuscular injection (vaccination), and blood test from a nurse with other students watching. Each of these procedures include the following steps:<br>• Consent process (e.g., nurse asking questions) and preparation (e.g., sleeve rolled up for injection and tourniquet applied for blood test).<br>• Seeing needle going into body (upper arm for injection and vein in arm for blood test). See blood coming out into vials for blood test. |


© Copyright: The University of Oxford and Oxford University Hospitals NHS Foundation Trust 2019

| | | |
|---|---|---|
| | | • Seeing wound left by needle before cotton wool/tape is applied.<br>• Choice of left/right arm for receiving procedures according to handedness.<br>• Stay in same location to receive different procedures but pause between each procedure.<br><br>*These are all simulations and not real procedures. |

### 10.3. Other Interventions

All participants will continue with their usual care, which is typically no provision of any help for needle fear.

## 11. SAFETY REPORTING

In our previous studies, we have not found adverse events linked to use of our automated VR therapies. VR therapy for needle fear should be very low risk. We will only be aware of adverse events if we are told by participants. The closest of our previous studies to the current trial is a test of an automated VR therapy for fear of heights (Freeman et al, 2018) – no serious adverse event occurred for any participant.

Adverse events (AEs) are reportable from the time of study enrolment. The time of enrolment is defined as the time at which, following recruitment, a participant and their parent/guardian signs and dates the assent and consent form.

### 11.1. Adverse Event Definitions

| | |
|---|---|
| Adverse Event (AE) | Any untoward medical occurrence in a participant, including occurrences which are not necessarily caused by or related to the medical device/research procedures. |
| Serious Adverse Event (SAE) | A serious adverse event is any untoward medical occurrence that:<br>• results in death<br>• is life-threatening<br>• requires inpatient hospitalisation or prolongation of existing hospitalisation<br>• results in persistent or significant disability/incapacity<br>• consists of a congenital anomaly or birth defect*.<br><br>Other 'important medical events' may also be considered a serious adverse event when, based upon appropriate medical judgement, the |


© Copyright: The University of Oxford and Oxford University Hospitals NHS Foundation Trust 2019

| | |
|---|---|
| | event may jeopardise the participant and may require medical or surgical intervention to prevent one of the outcomes listed above.<br><br>NOTE: The term "life-threatening" in the definition of "serious" refers to an event in which the participant was at risk of death at the time of the event; it does not refer to an event which hypothetically might have caused death if it were more severe. |

NB: to avoid confusion or misunderstanding of the difference between the terms “serious” and “severe”, the following note of clarification is provided: “Severe” is often used to describe intensity of a specific event, which may be of relatively minor medical significance. “Seriousness” is the regulatory definition supplied above.

### 11.2. Assessment results outside of normal parameters as AEs and SAEs

There are no assessments for which scores are considered as AEs or SAEs.

### 11.3. Assessment of Causality

The relationship between the VR therapy or other research procedure and the occurrence of an adverse event will be assessed and categorised. The team will use clinical judgement to determine the relationship. Alternative causes, such as natural history of the participant’s underlying condition, concomitant therapy, other risk factors etc. will be considered. The Investigator will also consult the current version of the risk analysis report.

| Classification | Relationship | Definition |
|---|---|---|
| Related | Causal relationship | • Starts within a time related to the study device/procedure *and*<br>• No obvious alternative medical explanation. |
| | Probable | • Starts within a time related to the study device/procedure *and*<br>• Cannot be reasonably explained by known characteristics of the participant’s clinical state. |
| | Possible | • Starts within a time related to the study device/procedure *and*<br>• A causal relationship between the intervention and the adverse event is at least a reasonable possibility. |
| Not related | Unlikely | • The time association or the participant’s clinical state is such that the study device/procedure is not likely to have had an association with the observed effect. |
| | Not related | • The AE is definitely not associated with the study device/procedure. |


© Copyright: The University of Oxford and Oxford University Hospitals NHS Foundation Trust 2019

### 11.4. Expectedness

The expectedness (i.e., anticipated or unanticipated) of adverse events will be determined by the research team. It is relatively common for this participant group to refuse needle procedures. We also note that a temporary increase in anxiety symptoms is expected in any psychological treatment involving confronting a feared situation.

### 11.5. Procedures for Reporting Adverse Events

All adverse events will be recorded on the Case Report Form (CRF) and reviewed by the study team for whether they are related to research procedures or treatment. The following information will be reported on the CRF: description, date of onset and end date, severity, assessment of relatedness to research treatment or procedures, and action taken. Follow-up information will be provided as necessary.

### 11.6. Reporting Procedures for Serious Adverse Events

All serious adverse events that come to our attention are reviewed by the study team. These include serious events which are:

- Related to the intervention and those which are non-intervention related;
- Anticipated and unanticipated serious events;
- Device Deficiencies that might have led to a serious adverse event if:

o Suitable action had not been taken or, intervention had not been made or,

o If circumstances had been less fortunate.

SAEs are recorded using the Serious Adverse Event Report Form. A serious adverse event (SAE) occurring to a participant should be reported to the REC that gave a favourable opinion of the study where in the opinion of the Chief Investigator the event was 'related' (resulted from administration of any of the research procedures) and 'unexpected' in relation to those procedures. Reports of related and unexpected SAEs should be submitted within 15 working days of the Chief Investigator becoming aware of the event, using the HRA report of serious adverse event form (see HRA website).

#### 11.6.1. Events exempt from immediate reporting as SAEs

Hospitalisation for a pre-existing condition, including elective procedures planned prior to study entry, which has not worsened, does not constitute a serious adverse event.

### 11.7. Medicines and Healthcare Products Regulatory Agency (MHRA) Reporting

The research team will follow their standard operating procedures for Incident Management (UO-SOP-E011) and Device Recalls and Advisory Notices (UO-SOP-E008). Any event which meets all three reporting


© Copyright: The University of Oxford and Oxford University Hospitals NHS Foundation Trust 2019

criteria below is considered an adverse incident and will be reported to the MHRA by the manufacturer of VR for Needle Fears (i.e., the research team) in line with Medical Device Guidelines (MEDDEV 2.12/1 rev 8):

- an event has occurred. This includes situations where testing performed on the VR for Needle Fears programme, examination of the information supplied with the device, or any scientific information indicates some factor that could lead, or has led, to an event;
- the VR for Needle Fears programme is suspected to be a contributory cause of the incident as determined by the research team;
- the event resulted in, or might have resulted, in death or a serious deterioration in state of health of a patient, user, participant or other person.

The timescales for reporting to the MHRA are set out in the table below.

| Incident Type | Reporting Timescale to national Competent Authority (e.g., MHRA in the UK) |
|---|---|
| Serious Public Health Threat | Immediately, but not later than 2 calendar days after University of Oxford is aware of the threat. |
| Death or unanticipated serious deterioration in state of health | Immediately after University of Oxford has established a link between the device and the event, but not later than 10 calendar days following the date of University of Oxford awareness of the event |
| Other | Immediately (without any delay that could not be justified) after University of Oxford has established a link between the device and the event but not later than 30 elapsed calendar days following the date of awareness of the event. If after becoming aware of a potentially reportable Incident there is still uncertainty about whether the event is reportable, University of Oxford will submit a report within the timeframe required for that type of Incident. |

## 12. STATISTICS

### 12.1. Statistical Analysis Plan (SAP)

For the cohort study the analysis will follow the description below, and a SAP will not be written. For the RCT a statistical analysis plan will be finalised before any analyses.

### 12.2. Description of Statistical Methods

For the cohort study, paired t-tests will be used to estimate mean differences and confidence intervals for the pre-post change in needle fear. P-values will not be reported. Effect sizes (Cohen's d) will be calculated by dividing the change score by the standard deviation of the baseline average score. A similar strategy


© Copyright: The University of Oxford and Oxford University Hospitals NHS Foundation Trust 2019

will be applied for the secondary outcome measures. Presentation of satisfaction, usability and side effects data will be descriptive.

For the RCT we will report data in line with the CONSORT 2018 SPI statement showing attrition rates and loss to follow-up. The target estimand is the treatment policy estimand and all primary and secondary analyses will be carried out following the intention to treat principle, incorporating data from all participants including those who do not complete treatment. Every effort will be made to follow up all participants in both arms for research assessments.

Analyses will be conducted in Stata version 17 or later. Descriptive statistics within each randomised group will be presented for baseline values. These will include counts and percentages for binary and categorical variables, and means and standard deviations, or medians with lower and upper quartiles, for continuous variables, along with minimum and maximum values and counts of missing values. There will be no tests of statistical significance or confidence intervals for differences between randomised groups on any baseline variable.

Treatment effects on primary and secondary outcomes will be estimated using linear mixed models fitted to outcome variables at all follow-up points. Fixed effects will be baseline assessment for the outcome under investigation, treatment, time and time*treatment interactions. Participant will be included as a random intercept to account for repeated measures. Marginal treatment effects will be estimated for the primary outcome at each time point, and reported separately as adjusted mean differences in scores between the groups with confidence intervals and 2-sided p-values. For secondary outcomes the same approach will be followed using linear mixed models to estimate and report the treatment effect at each time point. Cohen’s d effect sizes will be calculated as the adjusted mean difference of the outcome divided by the sample standard deviation of the outcome at baseline.

To test the moderation hypotheses, we will extend the analysis model for the primary outcome to include as fixed effects the putative moderator and its interaction with treatment; the coefficient of the interaction tests whether there is a differential treatment effect across levels of the moderator variable.

To test the mediation hypothesis, we will estimate causal mediation estimands using parametric regression models/structural equation models. All mediation analyses will be adjusted for baseline measures of the mediator, outcomes, and possible measured confounders.

### 12.3. Sample Size Determination

The cohort study is provisional proof of concept testing. A total of 12 participants is a similar size to our previous early stage testing of interventions (e.g. Freeman et al, 2016; Forkert et al, 2022; Freeman et al, submitted). For this first RCT test, our target sample size is 60 individuals, which would enable the trial to detect a standardised treatment effect of large size (d~0.87) with 90% power at a 5% level of significance (2-sided). This is based on a mean score for a blood-injection-injury phobia university student group on the outcome scale of 45 (SD=8.9) (Olatunji et al, 2010).

### 12.4. Analysis Populations


© Copyright: The University of Oxford and Oxford University Hospitals NHS Foundation Trust 2019

The RCT primary analyses will be carried out using the intention-to-treat principle. That is, after randomisation, participants will be analysed according to their allocated intervention arm irrespective of what intervention they actually receive, and with data available from all participants included in the analysis including those who do not complete therapy. Every effort will be made to follow up all participants in both arms for research assessments. If there is substantial missing data sensitivity analysis will be considered to assess the robustness of the result to different missing data assumptions.

### 12.5. Decision Points

Phase I data (proof of concept) will be inspected continuously. Phase II (RCT) will not proceed unless there is evidence of a moderate effect size (d=0.6) reduction in needle fears from Phase I. There are no interim analyses for Phase II.

### 12.6. Stopping Rules

We would stop if there was no evidence of efficacy in Phase I. The final decision would be made by the Chief Investigator. There will be no formal stopping rules for Phase II once it proceeds.

## 13. DATA MANAGEMENT

The data management aspects of the study are summarised here with details fully described in the Data Management Plan.

### 13.1. Source Data

Source documents are where data are first recorded, and from which participants' CRF data are obtained. These include the trial assessment measures.

We keep data from the assessments, collected on paper from the participant assessments. All documents, including electronic files, will be stored safely in confidential conditions. On all trial-specific documents, other than the signed consent, the participant will be referred to by the trial participant number, not by name. CRF entries will be considered source data if the CRF is the site of the original recording (i.e. there is no other written or electronic record of data) (e.g. self-report questionnaires).

### 13.2. Access to Data

Direct access will be granted to authorised representatives from the Sponsor, host institution and the regulatory authorities to permit trial-related monitoring, audits and inspections.

### 13.3. Data Recording and Record Keeping

All trial data will be entered on paper or electronic CRFs and transcribed or entered directly to the clinical data management system. Data will be pseudonymised using a unique study ID. This includes the data stored whilst participants are using the VR software (i.e., time spent using VR and activities completed).


© Copyright: The University of Oxford and Oxford University Hospitals NHS Foundation Trust 2019

Personal data and participant identification codes will be kept separately from the research data. Access to these data will be strictly on a need to know basis by members of the research team.

Data will be transferred from paper CRFs to the clinical database, or recorded directly on eCRFs as soon as possible after the study visit. Validation of all data entered into the clinical database is achieved through manual review. All critical data items are 100% checked against original source documents, where applicable, to ensure accuracy and an error rate is established across all fields to ensure a consistently accurate dataset.

Electronic data will be kept on a secure University of Oxford server. Hard copy of data will be kept in a locked filing cabinet in a locked room at University of Oxford facilities.

The report of the study will not identify any individuals. Research data, including consent forms, will be kept for 10 years following publication of the results. Any personal data which is not research data (e.g., contact details form) will be kept securely for 3-6 months after the study has finished in order for the research team to keep in contact with participants about the research study and share the findings.

## 14. QUALITY ASSURANCE PROCEDURES

### 14.1. Risk assessment

The trial will be conducted in accordance with the current approved protocol, GCP, relevant regulations and standard operating procedures. Data will be evaluated for compliance with the protocol, standard operating procedures, GCP, and accuracy in relation to source documents. A risk assessment and monitoring plan will be prepared before the study opens and will be reviewed as necessary over the course of the trial to reflect significant changes to the protocol or outcomes of monitoring activities.

### 14.2. Monitoring

Regular monitoring will be performed by the research team according to the trial specific Monitoring Plan. Data will be evaluated for compliance with the protocol and accuracy in relation to source documents as these are defined in the trial specific Monitoring Plan. Following written standard operating procedures, the monitors will verify that the clinical trial is conducted and data are generated, documented and reported in compliance with the protocol, GCP and the applicable regulatory requirements.

### 14.3. Trial committees

A Trial Management Group will meet monthly during the trial. This will be setup prior to commencement of the RCT.

## 15. PROTOCOL DEVIATIONS

A trial related deviation is a departure from the ethically approved trial protocol or other trial document or process (e.g. consent process or IMP administration) or from Good Clinical Practice (GCP) or any applicable regulatory requirements. Any deviations from the protocol will be documented in a protocol deviation form and filed in the trial master file.


© Copyright: The University of Oxford and Oxford University Hospitals NHS Foundation Trust 2019

## 16. SERIOUS BREACHES

The Medicines for Human Use (Clinical Trials) Regulations contain a requirement for the notification of "serious breaches" to the MHRA within 7 days of the Sponsor becoming aware of the breach.

A serious breach is defined as “A breach of GCP or the trial protocol which is likely to affect to a significant degree:

(a) the safety or physical or mental integrity of the subjects of the trial; or

(b) the scientific value of the trial”.

In the event that a serious breach is suspected the Sponsor must be contacted within 1 working day. In collaboration with the CI the serious breach will be reviewed by the Sponsor and, if appropriate, the Sponsor will report it to the REC committee, Regulatory authority and the relevant NHS host organisation within seven calendar days.

## 17. ETHICAL AND REGULATORY CONSIDERATIONS

### 17.1. Declaration of Helsinki

The Investigator will ensure that this trial is conducted in accordance with the principles of the Declaration of Helsinki.

### 17.2. Guidelines for Good Clinical Practice

The Investigator will ensure that this trial is conducted in accordance with relevant regulations and with Good Clinical Practice.

### 17.3. Approvals

The protocol, informed consent form, participant information sheet and any proposed advertising material will be submitted to an appropriate Research Ethics Committee (REC), HRA and host institution(s) for written approval.

The Investigator will submit and, where necessary, obtain approval from the above parties for all substantial amendments to the original approved documents.

The VR treatment (VR for Needle Fears) will be UKCA marked before use within the trial.

### 17.4. Other Ethical Considerations

We anticipate few ethical concerns for participants entering these trials. Participation in the trial does not change existing treatment receipt, so there is no disadvantage in taking part. Participants will be free to discontinue the study at any point, without needing to give an explanation. Participants are likely to feel anxious during the treatment, as they are being exposed to feared stimuli. However participants are informed of this and stimuli are presented in a graded order and participants can skip particular tasks if they wish. It is also the case that some participants may feel faint or even faint. This is why we have developed the treatment to be completed while sitting down. There will also be a person in the room


© Copyright: The University of Oxford and Oxford University Hospitals NHS Foundation Trust 2019

during the young person completing the VR therapy. Finally, the use of VR can lead to brief side effects (e.g. feelings of sickness) for a small proportion of participants. We inform participants of these potential difficulties and plan a response individually with each person.

### 17.5. Reporting

The CI shall submit once a year throughout the clinical trial, or on request, an Annual Progress Report to the REC, HRA (where required), host organisation, funder (where required) and Sponsor. In addition, an End of Trial notification and final report will be submitted to the REC, host organisation, and Sponsor.

### 17.6. Transparency in Research

Prior to the recruitment of the first RCT participant, the trial (Phase II) will have been registered on a publicly accessible database (on www.isrctn.com). The record in the ISRCTN registry will be kept up to date.

### 17.7. Participant Confidentiality

The study will comply with UK General Data Protection Regulation (UK GDPR) and Data Protection Act 2018, which require data to be de-identified as soon as it is practical to do so. The processing of the personal data of participants will be minimised by making use of a unique participant study number only on all study documents and any electronic databases. All documents will be stored securely and only accessible by study staff and authorised personnel. The study staff will safeguard the privacy of participants' personal data.

If the research team is concerned about the safety of a participant or others, risk and safeguarding procedures will be followed in accordance with relevant national policies and statutory guidance, and the University of Oxford Safeguarding Code of Practice: https://hr.admin.ox.ac.uk/safeguarding-at-risk-adults-and-children. Dr Eve Twivy is the safeguarding lead for this study and will therefore typically be the first point of contact for any questions or concerns related to the safeguarding of children in this study. If there are any concerns raised about a member of the research team, the University of Oxford Safeguarding Officer will be contacted (email: director.hr@admin.ox.ac.uk, phone: 01865 270152).

### 17.8. Expenses and Benefits

Participants will be compensated with a £10 voucher for their time for each research assessment outside of therapy sessions. Reasonable travel expenses (for the participant and their parent/guardian where applicable) for any visits additional to normal care will be reimbursed on production of receipts, or a mileage allowance provided as appropriate.

## 18. FINANCE AND INSURANCE

### 18.1. Funding


© Copyright: The University of Oxford and Oxford University Hospitals NHS Foundation Trust 2019

The principal funding is from the Beryl Alexander charity. Support is also received from the NIHR Oxford Health Biomedical Research Centre (https://oxfordhealthbrc.nihr.ac.uk/).

### 18.2. Insurance

The University has a specialist insurance policy in place which would operate in the event of any participant suffering harm as a result of their involvement in the research (Newline Underwriting Management Ltd, at Lloyd's of London). NHS indemnity operates in respect of the clinical treatment that is provided.

### 18.3. Contractual arrangements

Appropriate contractual arrangements will be put in place with all third parties.

## 19. PUBLICATION POLICY

The Chief Investigator will be involved in writing and reviewing drafts of the manuscripts, abstracts, press releases, and any other publications arising from the study. Authorship will be determined in accordance with the ICMJE guidelines and other contributors will be acknowledged. All participants will be offered a copy of the results.

## 20. DEVELOPMENT OF A NEW PRODUCT/ PROCESS OR THE GENERATION OF INTELLECTUAL PROPERTY (IP)

Ownership of IP generated by employees of the University vests in the University. The University will ensure appropriate arrangements are in place as regards any new IP arising from the trial.

## 21. ARCHIVING

Archiving will be completed after publication of the study outcome. Research data, including consent forms, will be stored securely at the University of Oxford for 10 years post publication. Hard copies of research data will be digitised for this long-term storage. Once the electronic versions have been created, the original hard copies of research data will be securely destroyed. The research data, with the exception of the consent forms, will be stored as anonymised data in the context of this long-term storage. Any personal data which is not research data (e.g., contact details form) will be kept securely for 3-6 months after the study has finished in order for the research team to keep in contact with participants about the research study and share the findings. Electronic data will be kept on a secure University of Oxford server. Any hard copies of data will be kept in a locked filing cabinet in a locked room at University of Oxford facilities.

## 22. REFERENCES

Silverman, W. K., & Albano, A. M. (1996). Anxiety disorders interview schedule for DSM-IV: Child version. Oxford University Press.Ayala, E.S., Meuret, A.E., & Ritz, T. (2009). Treatments for blood-injury-injection phobia. Journal of Psychiatric Research, 43, 1235-1242.


© Copyright: The University of Oxford and Oxford University Hospitals NHS Foundation Trust 2019

France, C. R., Ditto, B., France, J. L., & Himawan, L. K. (2008). Psychometric properties of the Blood Donation Reactions Inventory: a subjective measure of presyncopal reactions to blood donation. Transfusion, 48(9), 1820-1826.

Forkert, A., Brown, P., Freeman, D., & Waite, F. (2022). A compassionate imagery intervention for patients with persecutory delusions. Behavioural and Cognitive Psychotherapy, 50, 15-27.

Freeman, D., Bradley, J., Waite, F., Sheaves, B., DeWeever, N., Bourke, E., McInerney, J., Evans, N., Černis, E., Lister, R., Garety, P. & Dunn, G. (2016). Targeting recovery in persistent persecutory delusions: a proof of principle study of a new translational psychological treatment. Behavioural and Cognitive Psychotherapy, 44, 539-552.

Freeman, D., Freeman, J., Ahmed, M., Haynes, P., Beckwith, H., Rovira, A., Miguel, A.L., Ward, R., Bousfield, M., Riffiod, L., Kabir, T., Waite, F., & Rosebrock, L. (submitted). Automated VR therapy for improving positive self-beliefs and psychological wellbeing in young patients with psychosis: a proof of concept evaluation of Phoenix VR self-confidence therapy.

Freeman, D., Haselton, P., Freeman, J., Spanlang, B., Kishore, S., Albery, E., ... & Nickless, A. (2018). Automated psychological therapy using immersive virtual reality for treatment of fear of heights: a single-blind, parallel-group, randomised controlled trial. The Lancet Psychiatry, 5(8), 625-632.

Freeman, D., Lambe, S., Kabir, T., Petit, A., Rosebrock, L., Yu, L-M., Dudley, R., Chapman, K., Morrison, A., O'Regan, E., Aynsworth, C., Jones, J., Murphy, E., Powling, R., Galal, U., Grabey, J., Rovira, A., Martin, J., Hollis, C., Clark, D.M., Waite, F., & gameChange Trial Group (2022). Automated virtual reality therapy to treat agoraphobic avoidance and distress in patients with psychosis (gameChange): a multicentre, parallel-group, single-blind, randomised, controlled trial in England with mediation and moderation analyses. Lancet Psychiatry, 9, 375–388.

Freeman, D., Lambe, S., Yu, L. M., Freeman, J., Chadwick, A., Vaccari, C., ... & Loe, B. S. (2023). Injection fears and COVID-19 vaccine hesitancy. Psychological medicine, 53(4), 1185-1195.

Freeman, D., Rosebrock, L., Waite, F., Loe, B. S., Kabir, T., Petit, A., ... & Lambe, S. (2023). Virtual reality (VR) therapy for patients with psychosis: satisfaction and side effects. Psychological Medicine, 53(10), 4373-4384.Hiermeier, U.M. & Mofrad, L. (2020). Feasibility of one-session treatment for specific needle phobia in an adult IAPT service – a case series. The Cognitive Behaviour Therapist, 13, e51.

Jiang, M. Y., Upton, E., & Newby, J. M. (2020). A randomised wait-list controlled pilot trial of one-session virtual reality exposure therapy for blood-injection-injury phobias. Journal of Affective Disorders, 276, 636-645.

Lambe, S., Knight, I., Kabir, T., West, J., Patel, R., Lister, R., ... & Freeman, D. (2020). Developing an automated VR cognitive treatment for psychosis: gameChange VR therapy. Journal of Behavioral and Cognitive Therapy, 30(1), 33-40.

Lumley, M. A., & Melamed, B. G. (1992). Blood phobics and nonphobics: Psychological differences and affect during exposure. Behaviour research and therapy, 30(5), 425-434.

McLenon, J., & Rogers, M. A. (2019). The fear of needles: A systematic review and meta-analysis. Journal of advanced nursing, 75(1), 30-42.


© Copyright: The University of Oxford and Oxford University Hospitals NHS Foundation Trust 2019

McMurtry, C.M., Taddio, A., Noel, M., Antony, M.M., Chambers, C.T., Asmundson, G.J.G….Scott, J. (2016). Exposure-based interventions for the management of needle fear across the lifespan: a clinical practice guideline and call for further research. Cognitive Behaviour Therapy, 45, 217-235.

Muris, P., Huijding, J., Mayer, B., Langkamp, M., Reyhan, E., & Olatunji, B. (2012). Assessment of disgust sensitivity in children with an age-downward version of the Disgust Emotion Scale. Behavior therapy, 43(4), 876-886.

Oar, E. L., Farrell, L. J., Conlon, E. G., Waters, A. M., & Ollendick, T. H. (2017). Patterns of response and remission following a one-session treatment for blood-injection-injury phobia in youth. Child & Family Behavior Therapy, 39(1), 43-63.

Olatunji, B. O., Sawchuk, C. N., Moretz, M. W., David, B., Armstrong, T., & Ciesielski, B. G. (2010). Factor structure and psychometric properties of the Injection Phobia Scale–Anxiety. Psychological assessment, 22(1), 167.

Olatunji, B. O., Williams, N. L., Sawchuk, C. N., & Lohr, J. M. (2006). Disgust, anxiety and fainting symptoms associated with blood-injection-injury fears: a structural model. Journal of Anxiety Disorders, 20(1), 23-41.

Ollendick, T. H., Halldorsdottir, T., Fraire, M. G., Austin, K. E., Noguchi, R. J., Lewis, K. M., ... & Whitmore, M. J. (2015). Specific phobias in youth: A randomized controlled trial comparing one-session treatment to a parent-augmented one-session treatment. Behavior therapy, 46(2), 141-155.

Öst, L. G., Hellström, K., & Kåver, A. (1992). One versus five sessions of exposure in the treatment of injection phobia. Behavior therapy, 23(2), 263-281.

Taddio, A., Ipp, M., Thivakaran, S., Jamal, A., Parikh, C., Smart, S., Sovran, J. … Katz, J. (2012). Survey of the prevalence of immunization non-compliance due to needle fears in children and adults. Vaccine, 30, 4807-4812.


© Copyright: The University of Oxford and Oxford University Hospitals NHS Foundation Trust 2019

## 23. APPENDIX A: SCHEDULE OF PROCEDURES

| **Procedures** | **Visits** | | | | | |
|---|---|---|---|---|---|---|
| | **Screening** | **Baseline** | **Allocated to the VR therapy (treatment group)** | **end of therapy assessment** | **6-week follow-up** | **Optional VR therapy for control group** |
| **Case series** | | | | | | |
| Eligibility assessment | X | | | | | |
| Informed consent | | X | | | | |
| Demographics and Contact Information | | X | | | | |
| Specific Phobia subsection of Anxiety Disorder Interview Schedule - Child Version | | X | | | | |
| The Injection Phobia Scale- Anxiety (Child Version) | | X | | X | | |
| Behavioural Avoidance Task (BAT) | | X | | X | | |
| Visual Analogue Scale - Fear | | | X | | | |
| Blood Donations Reaction Inventory | | | X | | | |
| Oxford-VR Side Effects Scale | | | | X | | |
| Usability Questionnaire | | | | X | | |
| Child Treatment Satisfaction | | | | X | | |


© Copyright: The University of Oxford and Oxford University Hospitals NHS Foundation Trust 2019

| | | | | | | |
|---|---|---|---|---|---|---|
| Adverse events assessment | | X | X | X | | |
| VR therapy | | | X (all participants) | | | |
| Treatment as usual | X | X | X | X | | |
| **Randomised Controlled trial** | | | | | | |
| Eligibility assessment | X | | | | | |
| Informed consent | | X | | | | |
| Demographics and Contact Information | | X | | | | |
| Randomisation | | X | | | | |
| Specific Phobia subsection of Anxiety Disorder Interview Schedule - Child Version | | X | | | | |
| The Injection Phobia Scale- Anxiety (Child Version) | | X | | X | X | |
| Needle Procedure Tactile Sensations Questionnaire | | X | | | | |
| Disgust Emotion Scale for Children – Injections and Blood Draws Subscale | | X | | X | X | |
| Needle Cognitions Questionnaire | | X | | X | X | |
| Visual Analogue Scale - Fear | | | X | | | X |
| Blood Donations Reaction Inventory | | | X | | | X |
| Oxford-VR Side Effects Scale | | | X | | | X |
| Child Treatment Satisfaction | | | X | | | X |
| Adverse events assessment | | X | X | X | X | X |


© Copyright: The University of Oxford and Oxford University Hospitals NHS Foundation Trust 2019

| | | | | | | |
|---|---|---|---|---|---|---|
| VR therapy | | | X (treatment group only) | | | X (control group only) |
| Treatment as usual | X | X | X | X | X | X |


© Copyright: The University of Oxford and Oxford University Hospitals NHS Foundation Trust 2019

## 24. APPENDIX B: AMENDMENT HISTORY

| Amendment No. | Protocol Version No. | Date issued | Author(s) of changes | Details of Changes made |
|---|---|---|---|---|
| 1 | 1.2 | 14.06.2024 | Dr Eve Twivy | Addition of PICs |
| 2 | 1.3 | 10.07.2024 | Dr Eve Twivy | Update to participant procedures and total number of participants required for RCT. |
| 3 | 1.4 | 17.02.2025 | Dr Eve Twivy | Update to RCT dates and correction of typo. |

List details of all protocol amendments here whenever a new version of the protocol is produced.

Protocol amendments must be submitted to the Sponsor for approval prior to submission to the REC committee, HRA (where required) or MHRA.


© Copyright: The University of Oxford and Oxford University Hospitals NHS Foundation Trust 2019
