# Supplementary material for: Automated virtual reality therapy to treat needle fears (trypanophobia) in adolescents in England: a proof-of-concept cohort study and a Phase II randomised controlled trial
Source: eClinicalMedicine. 2026 Jul 15;97:104038. doi: 10.1016/j.eclinm.2026.104038 (PMC13420612; doi:10.1016/j.eclinm.2026.104038)

Primary Care Clinical Trials Unit

STATISTICAL ANALYSIS PLAN

VIRTUAL REALITY (VR) FOR NEEDLE FEARS: A COHORT STUDY AND A RANDOMISED CONTROLLED TRIAL OF AN AUTOMATED VR THERAPY FOR THE TREATMENT OF NEEDLE FEARS (TRYPANOPHOBIA)

Short title: VR for Needle Fears

Version number and date: 1.0, 28 November 2025

Based on protocol version 1.2, dated 17 February 2025

Written by: Martin Simera, Hugo Senra
Reviewed by: Ly-Mee Yu

Version History

Version:	Version Date:	Changes:
0.1	11 November 2025	
0.2	25 November 2025	Updated mediation analysis section
0.3	28 November 2025	Minor update on mediation analysis section and also Section 6.2 under sensitivity analysis.
1.0	28 November 2025	First version

Table of Contents

TABLE OF CONTENTS **3**

1. INTRODUCTION **5**

1.1 Preface 5
1.2 Purpose and scope of the plan 5
1.3 Trial overview 5
1.4 Objectives 6

2 TRIAL DESIGN **7**

2.1 Outcome measures 7
2.1.1 Primary outcome 7
2.1.2 Secondary outcomes 7
2.2 Target population 8
2.2.1 Inclusion Criteria 8
2.2.2 Exclusion Criteria 8
2.3 Sample size 9
2.4 Randomisation and blinding in the analysis stage 9

3 ANALYSIS – GENERAL CONSIDERATIONS **9**

3.1 Descriptive statistics 9
3.2 Characteristics of participants 9
3.3 Definition of population for analysis 10
3.4 Data Monitoring Committee And Interim Analyses 10

4 PRIMARY ANALYSIS **10**

4.1 Primary outcome(s) 10
4.2 Handling missing data 10
4.3 Handling outliers 11

4.4 MULTIPLE COMPARISONS AND MULTIPLICITY 11
4.5 MODEL ASSUMPTIONS 11

5 SECONDARY ANALYSIS 11

5.1 SECONDARY OUTCOMES 11
5.1.1 Continuous secondary outcomes 11
5.1.2 secondary outcomes collected following vr treatment session 12
5.1.3 mediation analysis 12
5.1.4 subgroup analysis 13

6 SENSITIVITY ANALYSIS 14

6.1 OUTLIERS 14
6.2 MISSINGNESS ASSUMPTIONS 14

7 SAFETY ANALYSIS 14

8 VALIDATION 14

9 CHANGES TO THE PROTOCOL OR PREVIOUS VERSIONS OF SAP 14

10 REFERENCES 15

11 APPENDICES 35

11.1 APPENDIX I. SCHEDULE OF PROCEDURES 35
APPENDIX II. FLOW DIAGRAM OF TRIAL PARTICIPANTS 38

1. INTRODUCTION

1.1 PREFACE

Chief Investigator: Professor Daniel Freeman

Trial Statisticians: Professor Ly-Mee Yu

1.2 PURPOSE AND SCOPE OF THE PLAN

This document details the analysis strategy for the main publication(s) reporting results from the Phase II of the Virtual Reality for Needle Fears study, funded by the Beryl Alexander Charity and the NIHR Oxford Health Biomedical Research Centre. Subsequent analyses of a more exploratory nature will not be bound by this analysis plan, though they are expected to follow the broad principles laid down here. The principles are not intended to curtail exploratory analysis, nor to prohibit accepted practices (such as data transformation prior to analysis), but they are intended to establish the rules that will be followed, as closely as possible, when analysing and reporting the trial.

1.3 TRIAL OVERVIEW

The hypodermic needle - used to inject substances (e.g., saline, medications, vaccinations) or extract fluids (e.g. blood) in clinical procedures – may be the most important medical device invented. Billions of needles are used worldwide each year. However, a significant minority of the population are very fearful of needles. This can make medical procedures unpleasant. It can also lead to avoidance of vaccination, blood donation and tests, and uptake of treatments. Fear of needles is especially high in children and adolescents. Needle fear can be successfully treated using psychological therapy (graded exposure and applied tension) but because of a shortage of therapists very few people are able to access such help.

The aim of this study is to assess the effectiveness for young people (ages 12-16 years) of an evidence-based psychological therapy within virtual reality for needle fear.

1.4 Objectives

	Objectives	Outcome Measures	Timepoint(s)
**Primary**	To test whether the VR therapy reduces needle fear	The Injection Phobia Scale-Anxiety (Child Version: Oar et al., 2017; Ost et al., 1992)	Baseline and 3 weeks (end of treatment)
**Secondary**	To test whether the reduction in needle fear persists	The Injection Phobia Scale-Anxiety (Child Version: Oar et al., 2017; Ost et al., 1992)	6 weeks
	To test whether the VR therapy has high satisfaction ratings	Child Treatment Satisfaction (Modified wording: Ollendick et al., 2015)	After treatment session (i.e. directly at the end of the treatment session)
	To test whether the VR therapy is associated with reduction in fearful	Needle Cognitions Questionnaire, Disgust Emotion Scale for Children – Injections and Blood Draws Subscale (Muris et al., 2012)	Baseline, 3 and 6 weeks

	cognitions and disgust reactions.		
	To test whether changes in needle-related fearful cognitions and disgust reactions mediate change in needle fear.	Needle Cognitions Questionnaire, Disgust Emotion Scale for Children – Injections and Blood Draws Subscale (Muris et al., 2012)	Baseline, 3 and 6 weeks
	To test whether there are moderators of the VR therapy.	The moderators tested will be age, gender, ethnicity, a history of fainting, and a modified specific phobia subsection of Anxiety Disorder Interview Schedule-Child Version (Silverman & Albano, 1996). Aversion to tactile sensations will also be measured using the Needle Procedure Tactile Sensations Questionnaire as an exploratory moderator.	
	To explore side effects, level of fear and vasovagal symptoms after using the VR therapy.	Modified Oxford-VR Side Effects Scale (Freeman et al., 2023), VAS scale-Fear, 4-item Blood Donation Reaction Inventory (France et al., 2008)	After treatment session (i.e., directly at the end of the treatment session)

2 TRIAL DESIGN

The study will utilise an individual, randomised, parallel group, clinical trial design and will aim to recruit 60 participants. The University of Oxford and Oxford Health NHS Foundation Trust (OHFT) are the research sites. Recruitment will be via the Oxfordshire School Aged Immunisation Service (SAIS), Berkshire School Aged Immunisation Team, Buckinghamshire School Aged Immunisation Team, School Health Nurses and general advertisements (e.g., via radio, social media and posters in schools/community venues).

Participants will be randomized using a 1:1 allocation ratio to receive the VR therapy or treatment as usual. Assessments will be conducted at Baseline, 3, and 6 weeks by a research assistant blind to group allocation. The assessments involve the participant completing questionnaires relevant to assessing their needle fear.

2.1 Outcome measures

2.1.1 Primary outcome

The primary outcome is participant self-reported needle fear using the Injection Phobia Scale- Anxiety (Child Version: Oar et al., 2017; Ost et al., 1992). Participants will complete the assessment at Baseline, 3 and 6 weeks. The primary outcome is needle fear at 3 weeks (end of treatment). The 6 week time point is a secondary outcome.

2.1.2 Secondary outcomes

2.1.2.1 self-reported needle fear

Participant self-reported needle fear at 6 weeks assessed using the Injection Phobia Scale-Anxiety (Child Version: Oar et al., 2017; Ost et al., 1992).

2.1.2.2 Treatment satisfaction

Participant satisfaction with the VR therapy will be assessed at the end of the treatment session using the modified Child Treatment Satisfaction (Ollendick et al., 2015).

2.1.2.3 Fearful cognitions and disgust reactions

Participants will complete the Needle Cognitions Questionnaire and Disgust Emotion Scale for Children– Injections and Blood Draws Subscale (Muris et al., 2012) at Baseline, 3 and 6 weeks

2.1.2.4 to examine if fearful cognition and disgust mediate change in needle fear

Mediation analyses will be performed to examine if changes in threat cognitions about needles (assessed using the Needle Cognitions Questionnaire) and disgust (assessed using the Disgust Emotion Scale for Children– Injections and Blood Draws Subscale (Muris et al., 2012) mediate change in needle fear with VR therapy.

2.1.2.5 SUBGROUP ANALYSES

Moderation analyses of the primary outcome (Injection Phobia Scale- Anxiety at 3 weeks) will be carried out for the following moderators assessed at baseline: age, gender, ethnicity, a history of
fainting, and a modified specific phobia subsection of Anxiety Disorder Interview Schedule-Child Version (Silverman & Albano, 1996). Aversion to tactile sensations will also be measured using the Needle Procedure Tactile Sensations Questionnaire as an exploratory moderator.

2.2 TARGET POPULATION

Participants are adolescents (aged 12-16 years old) with a needle fear. Recruitment will be via the Oxfordshire School Aged Immunisation Service (SAIS), Berkshire School Aged Immunisation Team, Buckinghamshire School Aged Immunisation Team, School Health Nurses and general advertisements (e.g., via radio, social media and posters in schools/community venues).

Individuals interested in the study will undergo eligibility assessment conducted by a research team member, most commonly a clinical psychologist or research assistant. This will typically be done remotely (i.e., online/telephone/video call). Participants must satisfy all the inclusion and exclusion criteria. A brief screening tool developed by the research team will be used to determine if a potential participant has a significant needle fear that they would like treated. The baseline assessment must commence within 4 weeks of the eligibility assessment. If it is after this period, a brief re-screening will be conducted to confirm eligibility.

2.2.1 Inclusion Criteria

- Aged 12-16 years old (up to 16th birthday).
- Have significant needle fears that they would like treated (as determined by a screening tool).
- Willing and able to give assent for participation in the study.
- A parent/guardian is willing and able to give informed consent for their child’s participation in the study.

2.2.2 EXCLUSION CRITERIA

- Photosensitive epilepsy or significant visual, auditory, or balance impairment that would make use of VR inappropriate.
- Current engagement in any other psychological treatment for needle fear.
- Command of English inadequate for engaging in the therapy or completing the assessments.
- A participant may also not enter the trial if there is another factor, which, in the judgement of the investigator, would preclude the provision of informed consent/assent or from safely engaging with the trial procedures. Reason for exclusion will be recorded.

2.3 Sample size

The target sample size is 60 individuals, which would enable the trial to detect a standardised treatment effect of large size (d~0.87) with 90% power at a 5% level of significance (2-sided). This is based on a mean score for a blood-injection-injury phobia university student group on the outcome scale of 45 (SD=8.9) (Olatunji et al, 2010).

2.4 Randomisation and blinding in the analysis stage

Participants in the randomised controlled trial will be randomised once they have completed the baseline assessment. Participants will be allocated to one of the trial arms using a 1:1 allocation ratio. Randomisation will be carried out by a validated online system provided by Sealed Envelope (www.sealedenvelope.com). Randomisation will use a permuted blocks algorithm, with randomly varying block size.

The research assessors will be blind to group allocation, but the participants and staff member present will not be (they cannot be blinded to whether a psychological intervention is delivered or not).

3 Analysis – General considerations

3.1 Descriptive statistics

Summary descriptions for continuous measurements will be means and standard deviations. Medians and interquartile ranges will be also presented if more appropriate, along with minimum and maximum values. Counts and percentages will be presented for categorical variables, including counts of missing data. Summary statistics will be provided by randomised group and overall.

3.2 Characteristics of participants

Baseline characteristics of the patients (demographics and baseline of the primary and all secondary outcome variables where available) will be reported by randomised group as well as the overall. There will be no tests of statistical significance nor confidence intervals for differences between randomised groups on any baseline variables.

In accordance with CONSORT guidelines, we will record and report participant flow (

Appendix II. Flow diagram of trial participants). Descriptive statistics of recruitment, drop-out, and completeness of interventions will be provided.

3.3 Definition of population for analysis

The target estimand is the treatment policy estimand and all primary and secondary analyses will be carried out following the intention to treat principle.

The primary analysis population will include all eligible participants for whom data are available. Participants will be analysed according to their randomised treatment assignment irrespective of the treatment they actually receive. Participants who withdraw from the trial will be included in the analysis until the point at which they withdraw.

The safety population will be all participants who received VR therapy or treatment as usual, analysed according to the intervention they actually received regardless of their randomised group.

3.4 Data Monitoring Committee And Interim Analyses

No interim analyses will be performed.

4 PRIMARY ANALYSIS

4.1 Primary outcome(s)

The primary comparison is the mean difference in the primary outcome (The Injection Phobia Scale-Anxiety) at 3 weeks for all randomised participants, as defined by protocol eligibility criteria, regardless of what intervention they actually received or compliance of intervention. Treatment effects on the primary outcome will be estimated using a linear mixed model fitted to Injection Phobia Scale-Anxiety score at all follow-up points. Fixed effects will be baseline assessment for Injection Phobia Scale-Anxiety score, treatment, time and time*treatment interactions. Participant will be included as a random intercept to account for repeated measures. Marginal treatment effects will be estimated for the Injection Phobia Scale-Anxiety score at the 3 week timepoint (primary outcome) and 6 week timepoint (secondary outcome), and reported separately as adjusted mean differences in scores between the groups with confidence intervals and 2-sided p-values.
By the principle of parsimony models will assume an unstructured covariance matrix for the random-effects (most flexible approach), and a diagonal structure for residuals, which assumes residuals (associated with observations on the same individual) to be uncorrelated and to have equal variances. The same model approach will be adopted for secondary outcomes analyses.

Cohen's d effect sizes will be calculated as the adjusted mean difference of the outcome divided by the sample standard deviation of the outcome at baseline.

4.2 Handling missing data

The mixed effects model will account for missing data assuming that data are missing at random (MAR). The availability of the outcome data will be summarised by randomised group.

4.3 Handling outliers

Any outliers will be checked and verified to ensure that they are true values. Outliers will be identified as those observations more than three standard deviations from the mean. Once they have been confirmed, a sensitivity analysis will be carried out to assess the impact of these values on the results by excluding these participants.

4.4 Multiple comparisons and multiplicity

The primary outcome is clearly stated in the protocol and no adjustments for multiple comparisons will be made.

4.5 Model assumptions

Standard residual diagnostics, such as inspection of the histogram of the residuals, will be used to assess the appropriateness of the model. If assumptions are violated, a transformation of the Injection Phobia Scale-Anxiety score, such as the log transformation will first be considered. If the assumptions are still violated alternative, and possibly non-parametric, approaches will be considered.

5 SECONDARY ANALYSIS

The primary analysis population will be used in all secondary analyses. For all analyses with mixed effect models, the same approach will be used as described in 4.1.

5.1 Secondary outcomes

5.1.1 Continuous secondary outcomes

Treatment effects on secondary outcomes will be estimated using linear mixed models fitted to outcome variables at all follow-up points. Fixed effects will be baseline assessment for the outcome under investigation, treatment, time and time*treatment interactions. Participant will be included as a random intercept to account for repeated measures. Marginal treatment effects will be estimated for the primary outcome at each time point, and reported separately as adjusted mean differences in scores between the groups with confidence intervals and 2-sided p-values. Cohen's d effect sizes will be calculated as the adjusted mean difference of the outcome divided by the sample standard deviation of the outcome at baseline.
These outcomes are:

- Disgust Emotion Scale for Children – Injections and Blood Draws Subscale (Muris et al., 2012)
- Needle Cognitions Questionnaire

The proposed analysis assumes that these secondary outcomes are continuous and satisfy the assumptions of the linear mixed effect model. Where these assumptions are not satisfied, the data will be transformed or if a transformation is not possible, a simpler model or a non-parametric approach to analysing the data will be adopted.

5.1.2 SECONDARY OUTCOMES COLLECTED FOLLOWING VR TREATMENT SESSION

For the following outcome measures collected directly at the end of a VR treatment session:

- Child Treatment Satisfaction (Modified wording: Ollendick et al., 2015)
- Modified Oxford-VR Side Effects Scale (Freeman et al., 2023),
- VAS scale-Fear
- 4-item Blood Donation Reaction Inventory (France et al., 2008)

Descriptive statistics for these outcome measures will be provided separately for participants randomised to receive the VR therapy intervention or control group participants offered VR therapy following completion of the 6-week follow-up assessment.

5.1.3 MEDIATION ANALYSIS

Mediation analyses will be performed to examine if changes in threat cognitions about needles (assessed using the Needle Cognitions Questionnaire) and disgust (assessed using the Disgust Emotion Scale for Children– Injections and Blood Draws Subscale (Muris et al., 2012) mediate change in needle fear with VR therapy.

In this mediation model, the association between the treatment variable (treatment randomisation: to receive the VR therapy or treatment as usual) and the outcome variable (needle fear) is explained via two mediators: changes in needles cognitions and disgust.

Mediation analysis will be performed using the counterfactual framework of causal inference, which, unlike structural equation modelling, proposes a single framework for the definition, identification, estimation, and sensitivity analysis of causal mediation effects, that is applicable beyond any specific statistical models (See Equations 1, 2 and 3) (Imai et al., 2010; Imai et al., 2011 Imai et al., 2013).Such framework is appropriate to perform mediation analysis under a mixed-model approach, which is also the same approach adopted for the trial primary analysis. Under the counterfactual framework, mediation analysis assumes sequential ignorability, which includes two key assumptions (Imai et el., 2010): (a) the treatment is independent of all potential values of the outcome and mediating variables, after controlling for relevant observed pretreatment covariates; b) the mediator is ignorable given the observed treatment and pretreatment confounders.

$$\delta_i(t) \equiv Y_i\big(t, M_i(1)\big) - Y_i\big(t, M_i(0)\big) \qquad (Equation\ 1)$$

$$\zeta_i(t) \equiv Y_i\big(1, M_i(t)\big) - Y_i\big(0, M_i(t)\big) \qquad (Equation\ 2)$$

$$\tau_i \equiv Y_i\big(1, M_i(1)\big) - Y_i\big(0, M_i(0)\big) = \frac{1}{2}\sum_{t=0}^{1}\{\delta_i(t) + \zeta_i(t)\} \qquad (Equation\ 3)$$

Equations 1 and 2 illustrate the single mediation framework, according to Imai et al. (2010). Y_i represents the observed outcome, M_i the mediator, and t the randomised treatment status. In Equation 1 $\delta_i(t)$ represents the causal mediation effect or indirect effects for each participant *i* for *t* = 0, 1. This is the indirect effect of the treatment on the outcome (*Y*) through the mediating variable (*M*). In Equation 2 $\zeta_i(t)$ represents the direct effect of the treatment (*t*) on the outcome (*Y*), for each participant *i*, holding the level of the mediator constant. The total effect of the treatment, τ_i (Equation 3) is decomposed into the causal mediation and direct effects.

Our mediation analysis will be implemented using the mediation algorithms proposed by Imai et al. (2010), through the *mediation* R package (Tingley et al., 2014). These mediation algorithms can accommodate a broad range of linear, non-linear, parametric and non-parametric statistical models, including linear and generalized mixed-models. For the main mediation analysis, confidence intervals will be calculated using the quasi-Bayesian Monte Carlo method with a minimum of 100 simulations. Sensitivity analysis will be carried out to check model quality, particularly for sequential ignorability assumptions.

5.1.4 SUBGROUP ANALYSIS

To test the moderation hypotheses, the analysis model for the primary outcome will be extended to include as fixed effects the putative moderator and its interaction with treatment; the coefficient of the interaction tests whether there is a differential treatment effect across levels of the moderator variable.

The following moderators will be considered:

- Age
- Gender
- Ethnicity
- History of fainting
- Phobia diagnosis (modified specific phobia subsection of Anxiety Disorder Interview Schedule-Child Version (Silverman & Albano, 1996))

Aversion to tactile sensations will also be measured using the Needle Procedure Tactile Sensations Questionnaire as an exploratory moderator.

Whether the effect of VR therapy on Injection Phobia Scale-Anxiety score is moderated by each of the three above factors will be tested. The interaction P value will be reported. In addition, the treatment effect and 95% CI for each subgroup (moderator level) will be reported.

6 SENSITIVITY ANALYSIS

6.1 OUTLIERS

If outliers are identified, a sensitivity analysis excluding these outliers will be carried out to determine the impact of these observations on the treatment effect of the primary outcome.

6.2 MISSINGNESS ASSUMPTIONS

Logistic regression models will explore any association between baseline characteristics and availability of the primary outcome. Missing primary outcome data will be reported overall and by randomised group.

For baseline covariates found to be predictive of missingness in the outcome ($P< 0.05$) using logistic regression models will be included in the analysis model in a sensitivity analysis of the primary outcome. Should any covariate (to be included in the model) have missing baseline data, the overall mean of the covariate at baseline will replace the missing values (as it is implausible that missingness will depend on randomised group given that baseline variables are measured prior to randomisation, and the interest is in the effect of treatment not the effect of the covariate so that all randomised participants with outcome data will be included in the analysis.

The missingness pattern of primary outcome assessments will be presented. The proportion of participants lost to follow-up and withdrawals in each group without data post-baseline will be given. A pattern mixture model will be fitted to assess the robustness of the MAR assumption required for the mixed effect regression model.

7 SAFETY ANALYSIS

Adverse events (AEs) and serious adverse events (SAEs) will be summarised descriptively according to the groups defined by the safety population, and may be split according to relatedness. The number (%) of participants with at least one SAE in the safety population will be reported. The total number of SAEs per randomised group will also be reported.

8 VALIDATION

As a minimum the primary analysis and safety data will be validated by a senior trial statistician (or delegate).

9 CHANGES TO THE PROTOCOL OR PREVIOUS VERSIONS OF SAP

10 REFERENCES

France, C. R., Ditto, B., France, J. L., & Himawan, L. K. (2008). Psychometric properties of the Blood Donation Reactions Inventory: a subjective measure of presyncopal reactions to blood donation. Transfusion, 48(9), 1820-1826.

Freeman, D., Rosebrock, L., Waite, F., Loe, B. S., Kabir, T., Petit, A., ... & Lambe, S. (2023). Virtual reality (VR) therapy for patients with psychosis: satisfaction and side effects. Psychological Medicine, 53(10), 4373-4384

Imai K, Keele L, Tingley D, Yamamoto T. Unpacking the Black Box of Causality: Learning about Causal Mechanisms from Experimental and Observational Studies. *American Political Science Review*. 2011;105(4):765-789. doi:10.1017/S0003055411000414

Imai K, Keele L, Tingley D. A general approach to causal mediation analysis. Psychol Methods. 2010 Dec;15(4):309-34. doi: 10.1037/a0020761.

Imai K, Tingley D, Yamamoto T. Experimental designs for identifying causal mechanisms. Royal Stat Soc. 2013 Jan;176:5-51. https://doi.org/10.1111/j.1467-985X.2012.01032.x

Muris, P., Huijding, J., Mayer, B., Langkamp, M., Reyhan, E., & Olatunji, B. (2012). Assessment of disgust sensitivity in children with an age-downward version of the Disgust Emotion Scale. Behavior therapy, 43(4), 876-886.

Oar, E. L., Farrell, L. J., Conlon, E. G., Waters, A. M., & Ollendick, T. H. (2017). Patterns of response and remission following a one-session treatment for blood-injection-injury phobia in youth. Child & Family Behavior Therapy, 39(1), 43-63.

Ollendick, T. H., Halldorsdottir, T., Fraire, M. G., Austin, K. E., Noguchi, R. J., Lewis, K. M., ... & Whitmore, M. J. (2015). Specific phobias in youth: A randomized controlled trial comparing one-session treatment to a parent-augmented one-session treatment. Behavior therapy, 46(2), 141-155.

Öst, L. G., Hellström, K., & Kåver, A. (1992). One versus five sessions of exposure in the treatment of injection phobia. Behavior therapy, 23(2), 263-281.

Silverman, W. K., & Albano, A. M. (1996). Anxiety disorders interview schedule for DSM-IV: Child version. Oxford University Press.Ayala, E.S., Meuret, A.E., & Ritz, T. (2009). Treatments for blood-injury-injection phobia. Journal of Psychiatric Research, 43, 1235-1242.

Tingley D, Yamamoto T, Hirose K, Keele L, Imai K. mediation: R Package for Causal Mediation Analysis. J. Stat. Soft. [Internet]. 2014 Sep. 2 [cited 2025 Nov. 20];59(5):1-38. Available from: https://www.jstatsoft.org/index.php/jss/article/view/v059i05.

11 Appendices

11.1 Appendix I. Schedule of procedures

**Procedures**	**Visits**					
	**Screening**	**Baseline**	**Allocated to the VR therapy (treatment group)**	**end of therapy assessment**	**6-week follow-up**	**Optional VR therapy for control group**
**Case series**						
Eligibility assessment	X					
Informed consent		X				
Demographics and Contact Information		X				
Specific Phobia subsection of Anxiety Disorder Interview Schedule - Child Version		X				
The Injection Phobia Scale- Anxiety (Child Version)		X		X		
Behavioural Avoidance Task (BAT)		X		X		
Visual Analogue Scale - Fear			X			
Blood Donations Reaction Inventory			X			
Oxford-VR Side Effects Scale				X		
Usability Questionnaire				X		
Child Treatment Satisfaction				X		

© Copyright: The University of Oxford and Oxford University Hospitals NHS Foundation Trust 2019

Adverse events assessment		X	X	X		
VR therapy			X (all participants)			
Treatment as usual	X	X	X	X		
**Randomised Controlled trial**						
Eligibility assessment	X					
Informed consent		X				
Demographics and Contact Information		X				
Randomisation		X				
Specific Phobia subsection of Anxiety Disorder Interview Schedule - Child Version		X				
The Injection Phobia Scale- Anxiety (Child Version)		X		X	X	
Needle Procedure Tactile Sensations Questionnaire		X				
Disgust Emotion Scale for Children – Injections and Blood Draws Subscale		X		X	X	
Needle Cognitions Questionnaire		X		X	X	
Visual Analogue Scale - Fear			X			X
Blood Donations Reaction Inventory			X			X
Oxford-VR Side Effects Scale			X			X
Child Treatment Satisfaction			X			X
Adverse events assessment		X	X	X	X	X

© Copyright: The University of Oxford and Oxford University Hospitals NHS Foundation Trust 2019

VR therapy			X (treatment group only)			X (control group only)
Treatment as usual	X	X	X	X	X	X

Appendix II. Flow diagram of trial participants

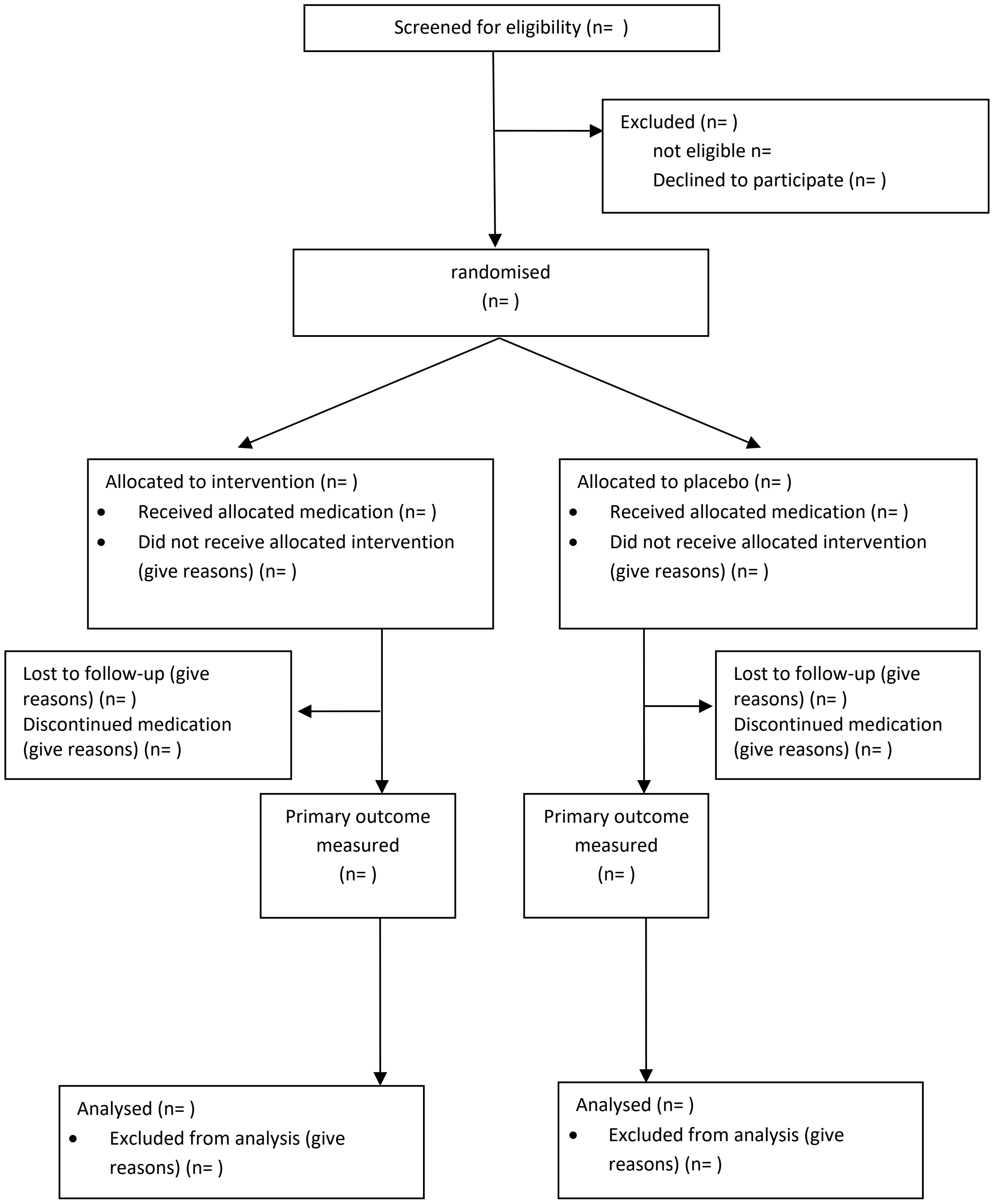

Supplement: Needles SAP_v1.0_28Nov2025 [file mmc5.pdf]
