# Supplementary material for: Automated virtual reality therapy to treat needle fears (trypanophobia) in adolescents in England: a proof-of-concept cohort study and a Phase II randomised controlled trial
Source: eClinicalMedicine. 2026 Jul 15;97:104038. doi: 10.1016/j.eclinm.2026.104038 (PMC13420612; doi:10.1016/j.eclinm.2026.104038)
Supplement: ST104-A_NEEDLES SAR_v0.4_clean [file mmc6.docx]

**ST101-A:**

**STATISTICAL ANALYSIS REPORT**

Virtual Reality (VR) for Needle Fears: A Cohort Study and a Randomised Controlled Trial of an Automated VR Therapy for the Treatment of Needle Fears (trypanophobia)

Short title: VR for Needle Fears

**CONFIDENTIAL**

**Version 0.4**

**06 May 2026**

Based on Protocol version

Based on SAP Version 1.0, 28th November 2025

Author: Hugo Senra

**Version History**

| **Version:** | **Version Date:** | **Changes:** |
| --- | --- | --- |
| 0.1 | 12 December 2025 | original |
| 0.2 | 19 December 2025 | Updated based on previous comments |
| 0.3 | 09 January 2026 | Updated interaction effect |
| 0.4 | 06 May 2026 | Updated mediation analysis with 2000 Monte Carlo draws. |
|  |  |  |

# Table of contents

[List of tables 3](#_Toc217037322)

[List of figures 4](#_Toc217037323)

[1 Introduction 5](#_Toc217037324)

[1.1 Validation 5](#_Toc217037325)

[1.2 Software employed 5](#_Toc217037326)

[2 Methods 6](#_Toc217037327)

[2.1 Background Information 6](#_Toc217037328)

[2.2 Trial/Study design 6](#_Toc217037329)

[2.3 Objectives 7](#_Toc217037330)

[2.4 Target population 7](#_Toc217037331)

[2.4.1 Inclusion Criteria 8](#_Toc217037332)

[2.4.2 Exclusion Criteria 8](#_Toc217037333)

[2.5 Interventions 8](#_Toc217037334)

[2.6 Outcomes measures 9](#_Toc217037335)

[2.7 Sample size 10](#_Toc217037336)

[2.8 Randomisation and blinding in the analysis stage 11](#_Toc217037337)

[2.9 Data cleaning 11](#_Toc217037338)

[2.10 Analysis for Data Monitoring and ethics Committee meetings 11](#_Toc217037339)

[2.11 Definition of population for analysis 11](#_Toc217037340)

[2.12 Deviation from SAP 11](#_Toc217037341)

[3 Results 12](#_Toc217037342)

[3.1 Representativeness of Study Sample and Patient Throughput 12](#_Toc217037343)

[3.2 Recruitment 13](#_Toc217037344)

[3.3 Baseline characteristics of participants 13](#_Toc217037345)

[3.4 Number analysed 14](#_Toc217037346)

[3.5 Primary Analyses 15](#_Toc217037347)

[3.5.1 Primary & Secondary Outcomes Analyses 15](#_Toc217037348)

[3.6 Mediation Analyses 17](#_Toc217037349)

[3.7 moderation analyses 19](#_Toc217037350)

[3.8 Sensitivity analyses 20](#_Toc217037351)

[4 References 20](#_Toc217037352)

[5 Appendices 23](#_Toc217037353)

[5.1 Appendix I. Full Mixed-Models and Diagnostic plots. 23](#_Toc217037354)

[27](#_Toc217037355)

[5.2. Appendix II. SENSITIVITY ANALYSIS 28](#_Toc217037356)

# List of tables

TABLE 1. Trial outcome measures ………………………………………………………………………………………………………….……….. 9

Table 2: Baseline characteristics by randomised group ……………………………………………………………………………….... 13

Table 3 Completion of follow-up assessments over the study period ………………………………………………………….… 14

Table 4: Summary statistics for the primary and secondary outcomes, and the treatment difference

and effect between the randomised groups ……………………………………….…………………………………………... 15

Table 5: Mediation models with the primary outcome at 3-weeks (end-of-treatment) ……………………………….… 18

Table 6: Mediation models with the primary outcome at 6-weeks (follow-up) ……………………………………………… 19

Table 7: Moderation analysis for the primary outcome at 3-weeks (end-of-treatment) ………………………………… 19

# List of figures

[Figure 1: Participant flow diagram 11](#_Toc217038871)

Figure 2: Marginal effects for randomised groups …………………………………………………………………………………………..15

# Introduction

This document details the analysis for the main paper(s) reporting results from the Phase II of the Virtual Reality for Needle Fears study, funded by the Beryl Alexander Charity and the NIHR Oxford Health Biomedical Research Centre. The results reported in these papers follow the strategy set out in the statistical analysis plan. Subsequent analyses of a more exploratory nature will not be bound by this strategy, though they are expected to follow the broad principles laid down here.

The analysis strategy will be available on request when the principal papers are submitted for publication in a journal. Suggestions for subsequent analyses by journal editors or referees, will be considered carefully, and carried out as far as possible in line with the principles of this analysis strategy; if reported, the source of the suggestion will be acknowledged.

This report is based on the statistical analysis plan *Needles SAP_v1.0_28Nov2025 stored on “K:\Stats\4. MISC\Needles\2. Stats Plan and Sample Size\SAP\Current”*. Any deviations from the statistical analysis plan will be described and justified in this report of the trial.

Trial/Study statistician(s):

Hugo Senra: [hugo.senra@phc.ox.ac.uk](mailto:hugo.senra@phc.ox.ac.uk)

Validation statistician(s):

Professor Ly-Mee Yu: [ly-mee.yu@phc.ox.ac.uk](mailto:ly-mee.yu@phc.ox.ac.uk)

Chief Investigator:

Professor Daniel Freeman: [daniel.freeman@psych.ox.ac.uk](mailto:daniel.freeman@psych.ox.ac.uk)

Trial/Study Manager:

Data Manager:

## Validation

Validation of results presented in this report was conducted by Professor Ly-Me Yu. The primary and safety endpoints were validated by independent programming using R-Studio-2025.09.2. Results from R-Studio outputs were checked for transcription errors. Further details of validation including validation programs are saved on the PC-CTU restricted drive in the project folder in the subfolder “STATS\4. Analysis\6.Validation - LMY”.

## Software employed

R-Studio-2025.09.2 was used for all analyses. Validation of analysis was carried out using Stata version 18.5

# Methods

## Background Information

For people with needle fears, needles have become associated with threat. People fear, for example, that the procedure will go wrong (e.g. the needle may snap, given wrong injection), or it will be very painful, or that they will have a panic attack or be unable to cope. Uniquely among anxiety disorders, part of a common response pattern for needle phobia is a drop in heart rate and blood pressure leading to fainting. People therefore also worry about fainting and its consequences. There is also evidence that disgust reactions may be a further contributory factor to needle, blood, and injury fears (Lumley & Melamed, 1992; Olatunji et al, 2006). Needle fear is treatable with psychological therapy. Exposure therapy (graded presentation of needle-related stimuli) and applied tension (learning to raise blood pressure when early signs of a drop in blood pressure are noticed) produce large reductions in blood-injection-injury fears (Ayala et al, 2009; McMurty et al, 2016; Hiermeier & Mofrad, 2020). However there is highly limited availability of such treatment.

Virtual reality (VR) may provide an approach to increasing the availability of effective treatment for needle fears. There are three key reasons. First, delivery of therapy can be automated within VR meaning that a therapist is not required. Second, VR can be a therapeutic medium since people find it easier to approach feared stimuli in VR since they know that it is not real but the learning made still transfers to the real- world. Finally, there is a potential in VR to present stimuli in ways that are therapeutic but impossible in the real world meaning that it could increase treatment efficacy. VR has already shown promise for treating needle fear. A randomised controlled trial with 43 adults with blood-injection-injury phobia showed that a 90-minute therapist-guided VR intervention reduced fear of injections (Jiang et al, 2020). We have previously developed several successful automated VR therapies (e.g. Freeman et al, 2018; Freeman et al, 2022). With young people with needle fears, we have now developed an automated VR therapy for needle fears. We have focussed on younger ages since this is when needle fears are most prevalent, there are several vaccinations to be given (e.g., HPV, Td/IPV, and MenACWY are typically given in year 8 and year 9), and there is likely value in earlier experiences of positive healthcare provision. The VR therapy if successful can also be easily adapted for older age groups.

## Trial/Study design

The study will utilise an individual, randomised, parallel group, clinical trial design and will aim to recruit 60 participants. The University of Oxford and Oxford Health NHS Foundation Trust (OHFT) are the research sites. Recruitment will be via the Oxfordshire School Aged Immunisation Service (SAIS), Berkshire School Aged Immunisation Team, Buckinghamshire School Aged Immunisation Team, School Health Nurses and general advertisements (e.g., via radio, social media and posters in schools/community venues).

Participants will be randomized using a 1:1 allocation ratio to receive the VR therapy or treatment as usual. Assessments will be conducted at Baseline, 3, and 6 weeks by a research assistant blind to group allocation. The assessments involve the participant completing questionnaires relevant to assessing their needle fear.

## Objectives

The aim of this study is to assess the effectiveness for young people (ages 12-16 years) of an evidence-based psychological therapy within virtual reality (VRT) for needle fear.

**The primary hypothesis** is that VRT will lead to a reduction in needle fear, at 3-weeks (end-of-treatment), in comparison with treatment as usual (TAU).

**The secondary hypotheses are:**

1. At 6 weeks, VRT, compared to TAU VRMR, the reduction in needle fear (treatment benefits) persists.

2. At 3 and 6-weeks, VRT, compared to TAU, will lead to a reduction in in fearful cognitions and disgust reactions.

3. At 3 and 6-weeks, improvements in needle-related fearful cognitions and disgust reactions mediate the relationship between treatment allocation (receiving VRT) and a reduction in needle fear.

4. Age, gender, ethnicity, history of fainting, and phobia are moderators of the VRT treatment.

## Target population

Participants are adolescents (aged 12-16 years old) with a needle fear. Recruitment will be via the Oxfordshire School Aged Immunisation Service (SAIS), Berkshire School Aged Immunisation Team, Buckinghamshire School Aged Immunisation Team, School Health Nurses and general advertisements (e.g., via radio, social media and posters in schools/community venues).

Individuals interested in the study will undergo eligibility assessment conducted by a research team member, most commonly a clinical psychologist or research assistant. This will typically be done remotely (i.e., online/telephone/video call). Participants must satisfy all the inclusion and exclusion criteria. A brief screening tool developed by the research team will be used to determine if a potential participant has a significant needle fear that they would like treated. The baseline assessment must commence within 4 weeks of the eligibility assessment. If it is after this period, a brief re-screening will be conducted to confirm eligibility.

### Inclusion Criteria

- Aged 12-16 years old (up to 16th birthday).
- Have significant needle fears that they would like treated (as determined by a screening tool).
- Willing and able to give assent for participation in the study.
- A parent/guardian is willing and able to give informed consent for their child’s participation in the study.

### Exclusion Criteria

- Photosensitive epilepsy or significant visual, auditory, or balance impairment that would make use of VR inappropriate.
- Current engagement in any other psychological treatment for needle fear.
- Command of English inadequate for engaging in the therapy or completing the assessments.
- A participant may also not enter the trial if there is another factor, which, in the judgement of the investigator, would preclude the provision of informed consent/assent or from safely engaging with the trial procedures. Reason for exclusion will be recorded.

## Interventions

The treatment being tested is automated VR therapy for needle fears. This software is intended to reduce needle fears. It is a cognitive-behavioural exposure and applied tension intervention. The treatment content was designed by the Oxford Cognitive Approaches to Psychosis (O-CAP) research group at the University of Oxford, with young people with lived experience taking part in the design process. The treatment was programmed by the University of Oxford. The treatment will achieve UKCA marking and registration as an individual Class I medical device (standalone software as a medical device) before the start of the testing.

The VR for needle fears software application is composed of a set of virtual environments, including different scenes created using 3D models, ambient audio, and 3D computer characters, with animations and speech. The environments are driven by source code which handles the logic of the program, the behaviour of the computer characters, as well as the user interaction and data storage. The software is built using Unity (Unity Technologies©). Unity acts as a render engine, displaying the virtual environments to the user through the headset.

The application will run through the Unity software application on a Meta Quest VR Headset. All technical requirements will be as per headset requirements. Accessory hardware and software are already commercially available and have not been modified for the VR therapy.

The programme takes approximately three hours to complete. It can be done in a half day or several shorter meetings on different days. The tasks are completed while sitting down. A research staff member will be present while the programme is used. The VR treatment sessions will typically take place at the University of Oxford (or a participant’s home/school/community venue where appropriate).

Key treatment components include: psychoeducation; applied tension technique to prevent fainting; graded exposure to feared situations; modelling by computer characters; encouragement and positive reinforcement.

## Outcomes measures

The table below outlines the objectives, outcome measures and time points of assessment for each outcome.

**TABLE 1. TRIAL OUTCOME MEASURES**

| **Objectives**  To test the following hypothesis: | **Outcome Measures** | **Timepoint(s) of evaluation of this outcome measure (if applicable)** |
| --- | --- | --- |
| **Primary hypothesis:** VRT will lead to a reduction in needle fear, at 3-weeks (end-of-treatment), in comparison with treatment as usual (TAU). | The Injection Phobia Scale-Anxiety (Child Version: Oar et al., 2017; Ost et al., 1992), giving a total score ranging from 0 to 72, where higher scores suggest greater levels of injection phobia. | Baseline and 3 weeks (end of treatment) |
| **Secondary Hypotheses:**  At 6 weeks, VRT, compared to TAU VRMR, the reduction in needle fear (treatment benefits) persists. | The Injection Phobia Scale-Anxiety (Child Version: Oar et al., 2017; Ost et al., 1992) | 6-weeks |
| To test whether the VR therapy has high satisfaction ratings. | Child Treatment Satisfaction (Modified wording: Ollendick et al., 2015) | After treatment session (i.e. directly at the end of the treatment session) |
| VR therapy is associated with reduction in fearful cognitions and disgust reactions. | Needle Cognitions Questionnaire, Disgust Emotion Scale for Children  – Injections and Blood Draws Subscale (Muris et al., 2012) | Baseline, 3 and 6 weeks |
| Changes in needle-related fearful cognitions and disgust reactions mediate change in needle fear. | Needle Cognitions Questionnaire, Disgust Emotion Scale for Children  – Injections and Blood Draws Subscale (Muris et al., 2012) | Baseline, 3 and 6 weeks |
| To test whether there are moderators of the VR therapy. | The moderators tested will be age, gender, ethnicity, a history of  fainting, and a modified specific phobia subsection of Anxiety Disorder Interview Schedule-Child  Version (Silverman & Albano, 1996). Aversion to tactile sensations will also be measured using the Needle Procedure Tactile Sensations Questionnaire as an exploratory moderator. | Baseline, 3-weeks |
| To explore side effects, level of fear and vasovagal symptoms after using the VR therapy. | Modified Oxford-VR Side Effects Scale (Freeman et al., 2023), VAS scale-Fear, 4-item Blood Donation Reaction Inventory (France et al., 2008) | After treatment session (i.e., directly at the end of the treatment session) |

## Sample size

The target sample size is 60 individuals, which would enable the trial to detect a standardised treatment effect of large size (d~0.87) with 90% power at a 5% level of significance (2-sided). This is based on a mean score for a blood-injection-injury phobia university student group on the outcome scale of 45 (SD=8.9) (Olatunji et al, 2010).

## Randomisation and blinding in the analysis stage

Participants in the randomised controlled trial will be randomised once they have completed the baseline assessment. Participants will be allocated to one of the trial arms using a 1:1 allocation ratio. Randomisation will be carried out by a validated online system provided by Sealed Envelope

(www.sealedenvelope.com). Randomisation will use a permuted blocks algorithm, with randomly varying block size.

The research assessors will be blind to group allocation, but the participants and staff member present will not be (they cannot be blinded to whether a psychological intervention is delivered or not).

## Data cleaning

Day to day data management was conducted by Psychiatry team. Additional data checking was also carried out by the statistics team and these files were saved on a restricted folder, “K:\Stats\3. OCHNCTU\NeedlesE\10. Data Cleaning”.

## Analysis for Data Monitoring and ethics Committee meetings

A Data Monitoring and Ethics Committee (DMEC) was formed with an independent clinician chair, independent statistician, and further independent clinician, to monitor the safety and progress of the trial. All details of analysis for DMC meetings are stored on a restricted folder, “K:\Stats\3. OCHNCTU\THRIVE\4. TSC and DMC\DMC”.

## Definition of population for analysis

The primary statistical analysis of efficacy outcomes will be carried out on the full analysis dataset. That is, after randomisation, participants will be analysed according to their allocated intervention group irrespective of what intervention they actually receive. Every effort will be made for full follow-up data on every participant to allow for as complete as possible analysis. If follow up data is missing for some participants, then any available data for those participants will be included in the primary analysis, under the missing at random (MAR) assumption.

## Deviation from SAP

The following outcomes were descriptive and not reported in this SAR.

- Child Treatment Satisfaction (Modified wording: Ollendick et al., 2015)
- Modified Oxford-VR Side Effects Scale (Freeman et al., 2023)
- VAS scale-Fear, 4-item Blood Donation Reaction Inventory (France et al., 2008)

# Results

## Representativeness of Study Sample and Patient Throughput

The diagram below shows the flow of participants through the trial. For each time point, ‘Reached’ is based on time from randomisation while ‘Completed’ implies that the primary outcome assessment was carried out and the primary outcome score is available for analysis.

Figure 1: Participant flow diagram

Reached 6-weeks (n = 30) Completed 6-weeks (n = 30) (follow-up)

Reached 6-weeks (n = 30) Completed 6-weeks (n = 30) (follow-up)

Reached 3-weeks (n = 30) Completed 3-weeks (n = 30) (end-of-treatment)

Reached 3-weeks (n = 30) Completed 3-weeks (n = 30) (end-of-treatment)

Randomised to TAU (n = 30 )

Randomised to VRT (n = 30 )

Participants Randomised (n = 60)

## Recruitment

The first participant was randomised on 28^th^ October 2024. The trial closed to recruitment on 15^th^ July 2025 with 60 participants randomized.

The trial was not stopped early. Final follow-up data were collected on 26^th^ August 2025.

## Baseline characteristics of participants

The table below (Table 2) summarises the baseline characteristic for the participants overall as well as separately for the two treatment groups. In general, the groups appear to be balanced.

Table 2: Baseline characteristics by randomised group

|  | **Overall**  N = 60*^1^* | **TAU**  N = 30*^1^* | **VRT**  N = 30*^1^* |
| --- | --- | --- | --- |
| **Age** |  |  |  |
| Mean (SD) | 13.4 (0.9) | 13.5 (0.9) | 13.2 (0.9) |
| (Min, Max) | (12.0, 15.0) | (12.0, 15.0) | (12.0, 15.0) |
| Median (IQR) | 13.0 (13.0,14.0) | 13.0 (13.0,14.0) | 13.0 (13.0,14.0) |
| **Currently at school year** |  |  |  |
| Year 7 | 2 (3%) | 0 (0%) | 2 (7%) |
| Year 8 | 15 (25%) | 6 (20%) | 9 (30%) |
| Year 9 | 31 (52%) | 17 (56%) | 14 (47%) |
| Year 10 | 9 (15%) | 5 (17%) | 4 (13%) |
| Year 11 | 3 (5)% | 2 (7%) | 1 (3%) |
| **Gender** |  |  |  |
| Male | 26 (43%) | 12 (40%) | 14 (47%) |
| Female | 32 (53%) | 17 (57%) | 15 (50%) |
| Other | 2 (3.3%) | 1 (3.3%) | 1 (3.3%) |
| **Ethnicity** |  |  |  |
| White | 57 (95%) | 28 (93%) | 29 (97%) |
| Other | 3 (5%) | 2 (7%) | 1 (3%) |
| **ADIS diagnosis** |  |  |  |
| No | 3 (5%) | 1 (3%) | 2 (7%) |
| Yes | 56 (95%) | 28 (96%) | 28 (93%) |
| *Misisng* | *1* | *1* | *0* |
| **ADIS CSR, n** | 56 | 28 | 28 |
| Mean (SD) | 5.4 (0.7) | 5.4 (0.7) | 5.4 (0.7) |
| (Min, Max) | (4.0, 7.0) | (4.0, 7.0) | (4.0, 7.0) |
| Median (IQR) | 5.0 (5.0,6.0) | 5.0 (5.0,6.0) | 5.0 (5.0,6.0) |
| **TSQ** |  |  |  |
| Mean (SD) | 9.1 (4.2) | 9.4 (5.0) | 8.9 (3.5) |
| (Min, Max) | (1.0, 20.0) | (1.0, 20.0) | (2.0, 16.0) |
| Median (IQR) | 9.0 (6.0,12.0) | 10.0 (6.0,13.0) | 9.0 (7.0,11.0) |
| *Missing* | *1* | *1* | *0* |
| **IPS** |  |  |  |
| Mean (SD) | 40.0 (10.0) | 40.1 (10.2) | 40.0 (9.6) |
| (Min, Max) | (15, 59) | (15, 55) | (17, 59) |
| Median (IQR) | 41 (34,47) | 42 (32,48) | 40 (35,47) |
| **NCQ** |  |  |  |
| Mean (SD) | 32.2 (12.0) | 31.2 (12.2) | 33.2 (12.5) |
| (Min, Max) | (8, 59) | (10, 59) | (8, 56) |
| Median (IQR) | 31 (24,41) | 31 (23,38) | 33 (24,43) |
| **DES** |  |  |  |
| Mean (SD) | 12.9 (5.5) | 12.2 (6.1) | 13.7 (4.8) |
| (Min, Max) | (3.0, 24.0) | (3.0, 24.0) | (5.0, 22.0) |
| Median (IQR) | 13.0 (8.0,17.5) | 11.5 (8.0,17.0) | 14.0 (10.0,18.0) |

*^1^*n (%);M (SD): Mean (Standard deviation); IQR: Interquartile range; ADIS CSR: Clinician's Severity Rating from the Anxiety Disorders Interview Schedule Anxiety; TSQ: Needles Procedure Tactile Sensation Questionnaire; IPS: Injection Phobia Scale-Anxiety; NCQ: Needles Cognition Questionnaire; DES: Disgust Emotion Scale for Children – Injections and Blood Draws subscale.

## Number analysed

The table below (Table 3) summarises the number of participants analysed at the 3 and 6-week time points, showing no dropout throughout the trial.

Table 3 Completion of follow-up assessments over the study period

|  | **VRT** | **TAU** | **Overall** |
| --- | --- | --- | --- |
| **Randomised, n** | 30 | 30 | 60 |
| 3-week primary outcome score available, n (%) | 30 (100) | 30 (100) | 60 (100) |
| 6-week primary outcome score available, n (%) | 30 (100) | 30 (100) | 60 (100) |

## Primary Analyses

### Primary & Secondary Outcomes Analyses

The primary objective was to compare the effect of treatment on reducing needle fear, at 3-weeks (end-of-treatment), in comparison with treatment as usual (TAU). In addition, we wanted to investigate the longer-term (6-weeks) intervention effect on primary and secondary outcomes.

Table 4 presents the results from the primary and secondary outcomes analyses, including the corresponding effect sizes at 3 and 6-weeks and treatment effect based on random-intercept mixed-models. Treatment effects (marginal effects) estimated from mixed-models are illustrated in Figure 2. Results suggest that the intervention (VRT), compared to TAU (controls), is effective in reducing needle fear at 3 and 6-weeks, with large effect sizes (Cohen’s d) at end-of-treatment (3-weeks) and at follow-up (6-weeks). Results also suggested that the intervention (VRT), compared to TAU (controls), is effective to reduce fearful cognitions and disgust reactions, at 3 and 6-weeks. The magnitude of treatment effect (Cohen’s d effect sizes) was large at 3 and 6-weeks for reducing fearful cognitions, and medium at 3 and 6-weeks for reducing disgust reactions. Full mixed models results and corresponding model diagnostics are presented in ***Appendix I.*** *Full Mixed-Models and Diagnostic plots.*

Table 4: Summary statistics for the primary and secondary outcomes, and the treatment difference and effect between the randomised groups

|  | **TAU** | | **VRT** | | **Cohen’s d**  **[95%CI]** | **Estimated [95% CI]**  **Treatment Effect^1^** | **SE^1^** | **T-Statistic^1^** | **ρ-value^1^** |
| --- | --- | --- | --- | --- | --- | --- | --- | --- | --- |
|  | N | Mean(SD) | N | Mean(SD) |  |  |  |  |  |
| **IPS** |  |  |  |  |  |  |  |  |  |
| Baseline | 30 | 40.06 (10.15) | 30 | 40.03 (9.59) |  |  |  |  |  |
| 3-Weeks (end-of-treatment) | 30 | 39.63 (11.77) | 30 | 25.53 (9.08) | 1.34 [0.77, 1.90] | -14.07 [-17.40, -10.73] | 1.72 | -8.19 | <0.001 |
| 6-Weeks (follow-up) | 30 | 39.40 (11.91) | 30 | 24.70 (9.11) | 1.39 [0.82, 1.95] | -14.67 [-18.00, -11.33] | 1.72 | -8.54 | <0.001 |
| **NCQ** |  |  |  |  |  |  |  |  |  |
| Baseline | 30 | 31.23 (12.22) | 30 | 33.17 (12.48) |  |  |  |  |  |
| 3-Weeks (end-of-treatment) | 30 | 31.37 (13.31) | 30 | 21.40 (10.92) | 0.82 [0.29,1.34] | -11.90 [-16.07, -7.73] | 2.15 | -5.23 | <0.001 |
| 6-Weeks (follow-up) | 30 | 32.30 (14.66) | 30 | 19.27 (10.55) | 1.02 [0.48, 1.56] | -14.97 [-19.14, -10.79] | 2.15 | -5.90 | <0.001 |
| **DES** |  |  |  |  |  |  |  |  |  |
| Baseline | 30 | 12.17 (6.11) | 30 | 13.67 (4.81) |  |  |  |  |  |
| 3-Weeks (end-of-treatment) | 30 | 12.77 (7.56) | 30 | 9.03 (4.95) | 0.58 [0.06, 1.10] | -5.23 [-7.14, -3.32] | 0.98 | -5.32 | <0.001 |
| 6-Weeks (follow-up) | 30 | 12.53 (7.80) | 30 | 8.13 (4.83) | 0.68 [0.15, 1.20] | -5.90 [-7.81, -3.99] | 0.98 | -5.99 | <0.001 |

IPS: The Injection Phobia Scale-Anxiety Scale; NCQ: Needle Cognitions Questionnaire; DES: Disgust Emotion Scale for Children; TAU: Treatment as usual; VRT: Virtual Reality Therapy; SE: Standard Error; M: Mean; SD: Standard Deviation; ^1^ Results from random-intercept mixed-models, for the interaction Randomised group x Assessment.


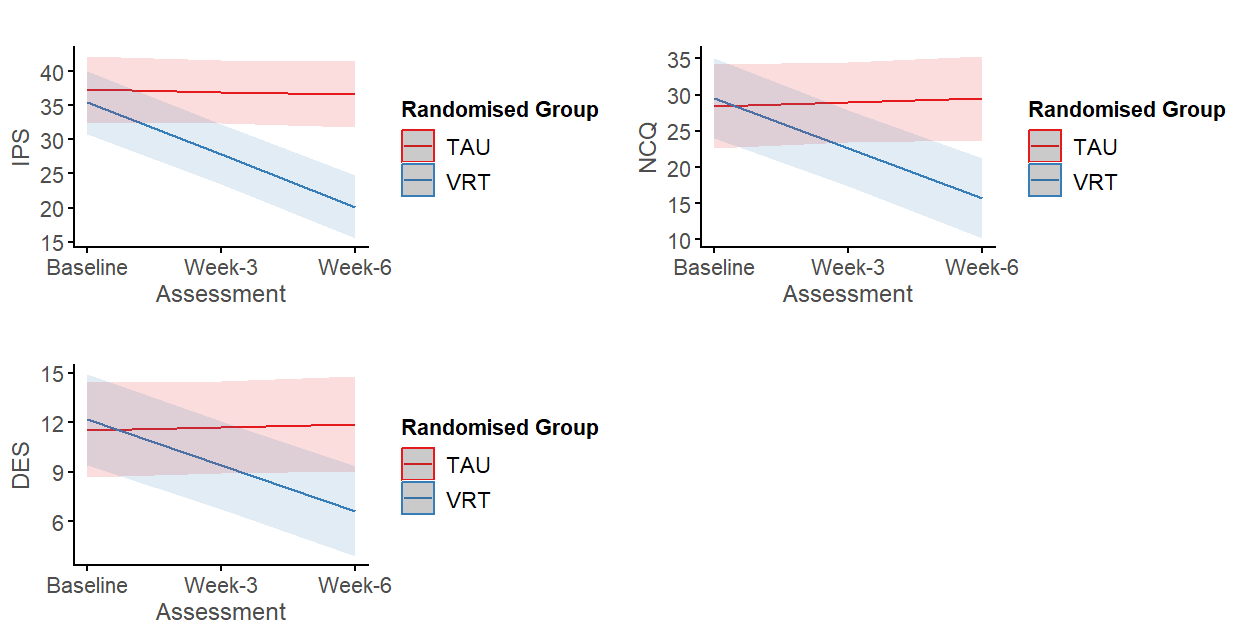


**Figure 2. Marginal effects for randomized groups (VRT vs tau) at end-of-treatment (3-weeks) and follow-up (6-weeks)**

IPS: The Injection Phobia Scale-Anxiety Scale; NCQ: Needle Cognitions Questionnaire; DES: Disgust Emotion Scale for Children; TAU: Treatment as usual; VRT: Virtual Reality Therapy

##

## Mediation Analyses

The mediation analyses were carried out using the counterfactual framework of causal inference which proposes a single framework for the definition, identification, estimation, and sensitivity analysis of causal mediation effects, that is applicable beyond any specific statistical models. For our mediation analysis, random-intercept mixed-models were adopted to estimate mediation paths, accounting for time effect (assessments), treatment randomisation, and its interaction with the mediator.

A representation of the mediation analysis following the Baron and Kenny (1986) approach of the tested pathways is shown below.

***The amount of mediation is called the indirect effect.***

***Total effect (c) = Direct effect (c’) + Indirect effect (ab)***

Randomisation VRT+TAU

Treatment outcome:

Needle fear

Total effect c

Randomisation to VRCT+TAU (control)

1. Fearful needles cognitions
2. Emotion disgust

Treatment outcome:

Needle fear

Direct effect c’

a

b

Tables 5 and 6 shows the corresponding mediation models results for the two mediators at 3-weeks (end-of-treatment) and at 6-weeks (follow-up).

There was no evidence that needle cognitions and emotion disgust mediate the association between intervention groups and needle fear at 3-week (Table 5). However, results do suggest evidence that needles cognitions mediate the association between group randomisation (VRT vs TAU) and needle fear (primary outcome), at 6-weeks (follow-up) (Table 6). According to the mediation model, individuals who received VRT intervention, compared to controls, showed a reduction in fearful needle cognitions, which in turn is associated with a reduction in need fear (primary outcome), with an average proportion mediated effect of 0.43 (95%CI: 0.11, 0.69). Finally, mediation model results do not suggest evidence that emotion disgust was not statistically significant mediator for the association between group randomisation (VRT vs TAU) and needle fear (primary outcome), at 6-weeks (follow-up) (Table 6).

**TABLE 5: MEDIATION MODELS WITH THE PRIMARY OUTCOME AT 3-WEEKS (END-OF-TREATMENT)**

|  | Estimate [95%CI] | | *p-value* | |
| --- | --- | --- | --- | --- |
|  | Mediator 1 | Mediator 2 | Mediator 1 | Mediator 2 |
| ACME (control) | -2.77 [-6.97, 1.19] | -1.23 [-4.63, 2.08] | 0.18 | 0.45 |
| ACME (treated) | -2.51 [-5.95, 0.57] | -1.72 [-5.02, 1.19] | 0.12 | 0.24 |
| ADE (control) | -4.51 [-8.17, -1.10] | -5.40 [-8.95, -1.94] | 0.005 | 0.002 |
| ADE (treated) | -4.25 [-7.80, -0.80] | -5.88 [-9.28, -2.42] | 0.02 | <0.001 |
| Total Effect | -7.02 [-11.95, -2.24] | -7.11 [-11.55, -2.61] | 0.003 | <0.001 |
| Prop. Mediated (control) | 0.40 [-0.35, 0.84] | 0.17 [-0.58, 0.53] | 0.17 | 0.45 |
| Prop. Mediated (treated) | 0.36 [-0.18, 0.73] | 0.23 [-0.31, 0.59] | 0.12 | 0.25 |
| ACME (average) | -2.64 [-6.22, 0.78] | -1.48 [-4.70, 1.59] | 0.15 | 0.35 |
| ADE (average) | -4.38 [-7.91, -1.13] | -5.64 [-9.00, -2.29] | 0.009 | <0.001 |
| Prop. Mediated (average) | 0.38 [-0.24, 0.76] | 0.20 [-0.43, 0.53] | 0.14 | 0.35 |

**Mediation Models** to test whether changes in needle-related fearful cognitions and emotion disgust mediate the relationship between VR treatment and change in needle fear at 3-weeks post-treatment. ACME: Average Causal Mediation Effects; ACE: Average Direct Effects; Prop. Mediated: Proportion Mediated (Indirect effect / Total effect). 95% confidence intervals calculated using the quasi-Bayesian Monte Carlo method with 2000 simulations.

**TABLE 6: MEDIATION MODELS WITH THE PRIMARY OUTCOME AT 6-WEEKS (FOLLOW-UP)**

|  | Estimate [95%CI] | | *p-value* | |
| --- | --- | --- | --- | --- |
|  | Mediator 1 | Mediator 2 | Mediator 1 | Mediator 2 |
| ACME (control) | -4.17 [-8.05, -0.65] | -2.15 [-5.25, 0.70] | 0.02 | 0.14 |
| ACME (treated) | -3.99 [-7.73, -0.65] | -2.94 [-6.97, 0.71] | 0.03 | 0.12 |
| ADE (control) | -5.58 [-9.15, -2.18] | -6.62 [-10.21, -3.18] | 0.001 | <0.001 |
| ADE (treated) | -5.39 [-8.60, -2.02] | -7.40 [-10.85, -4.00] | 0.001 | <0.001 |
| Total Effect | -9.57 [-14.34, -4.90] | -9.56 [-14.17, -4.77] | <0.001 | <0.001 |
| Prop. Mediated (control) | 0.44 [0.10, 0.74] | 0.22 [-0.11, 0.47] | 0.02 | 0.14 |
| Prop. Mediated (treated) | 0.42 [ 0.09, 0.69] | 0.30 [-0.11, 0.47] | 0.03 | 0.12 |
| ACME (average) | -4.08 [-7.59, -0.70] | -2.55 [-5.85, 0.66] | 0.02 | 0.13 |
| ADE (average) | -5.48 [-8.92, -2.22] | -7.01 [-10.47, -3.71] | <0.001 | <0.001 |
| Prop. Mediated (average) | 0.43 [0.11, 0.69] | 0.26 [-0.11, 0.52] | 0.02 | 0.13 |

**Mediation Models** to test whether changes in needle-related fearful cognitions and emotion disgust mediate the relationship between VR treatment and change in needle fear at 6-weeks post-treatment. ACME: Average Causal Mediation Effects; ACE: Average Direct Effects; Prop. Mediated: Proportion Mediated (Indirect effect / Total effect). 95% confidence intervals calculated using the quasi-Bayesian Monte Carlo method with 2000 simulations.

## moderation analyses

Moderation analyses were conducted to investigate whether the effect of VR therapy on Injection Phobia Scale-Anxiety score is moderated by the following baseline covariates: Age; Gender; Ethnicity; History of fainting; Phobia diagnosis (modified specific phobia subsection of Anxiety Disorder Interview Schedule-Child Version (Silverman & Albano, 1996)). Results are presented in Table 7, suggesting that none of the included baseline covariates had a significant moderation effect on the primary outcome.

**TABLE 7: MODERATION ANALYSIS FOR THE PRIMARY OUTCOME AT 3-WEEKS (END-OF-TREATMENT)**

| **Moderator*** | **VRT**  **(N = 30)** | **TAU**  **(N = 30)** | **Interaction effect [95% CI]**** | **Test of Interaction**  **p-value**** |
| --- | --- | --- | --- | --- |
| Age, n | 30 | 30 | 0.04 [-4.54, 4.61] | 0.988 |
| Gender [female]^1^, n | 30 | 30 | -5.91 [-13.34, 1.52] | 0.138 |
| Gender [other]^1^, n | 30 | 30 | -10.07 [-30.66, 10.52] | 0.360 |
| Ethnicity [other]^2^, n | 30 | 30 | 15.15 [-3.73, 34.02] | 0.128 |
| ADIS Diagnosis [yes]^3^, n | 30 | 30 | 6.71 [-12.71, 26.14] | 0.509 |
| ADIS Faint [yes]^4^, n | 30 | 30 | -0.65 [-11.19, 5.47] | 0.520 |
| ADIS Faint [other]^4^, n | 30 | 30 | -1.53 [-35.14, 3.70] | 0.132 |
| TSQ, n | 30 | 30 | -0.57 [-1.58, 0.43] | 0.278 |

*All moderators have been assessed at baseline; VRT: Virtual Reality Therapy; TAU: Treatment as Usual; **Interaction effect and test of interaction results from separate mixed-models testing a three-way interaction (randomisation group (VRT vs TAU)*assessment (baseline vs 3-weeks)*moderator; ^1^Gender groups include female, male and other; ^2^Ethinicity groups include white and other; ADIS: Anxiety Disorder Interview Schedule-Child Version; ^3^ADIS Diagnosis: no vs yes; ^4^ADIS Faint: no vs yes vs other. TSQ: Needles Procedure Tactile Sensations Questionnaire.

## Sensitivity analyses

Sensitivity analyses have been planned to investigate the potential influence of any identified outliers affecting our regression models. After checking distributions of residuals for all regression models, we identified one regression affected by extreme values causing residual distribution to not be normal (Shapiro Wilk test with p = 0.025). A new random-intercept model has been run without extreme values (z < 3), with results not different from the model with outliers (see ***Appendix II.*** *Sensitivity Analysis.* for mixed-model with Disgust Emotion Scale as dependent variable).

# References

Ayala, E.S., Meuret, A.E., & Ritz, T. (2009). Treatments for blood-injury-injection phobia. Journal of Psychiatric Research, 43, 1235-1242.

France, C. R., Ditto, B., France, J. L., & Himawan, L. K. (2008). Psychometric properties of the Blood Donation Reactions Inventory: a subjective measure of presyncopal reactions to blood donation. Transfusion, 48(9), 1820-1826.

Freeman, D., Haselton, P., Freeman, J., Spanlang, B., Kishore, S., Albery, E., ... & Nickless, A. (2018). Automated psychological therapy using immersive virtual reality for treatment of fear of heights: a single- blind, parallel-group, randomised controlled trial. The Lancet Psychiatry, 5(8), 625-632.

Freeman, D., Lambe, S., Kabir, T., Petit, A., Rosebrock, L., Yu, L-M., Dudley, R., Chapman, K., Morrison, A.,

Freeman, D., Rosebrock, L., Waite, F., Loe, B. S., Kabir, T., Petit, A., ... & Lambe, S. (2023). Virtual reality (VR) therapy for patients with psychosis: satisfaction and side effects. Psychological Medicine, 53(10), 4373-4384.

Hiermeier, U.M. & Mofrad, L. (2020). Feasibility of one-session treatment for specific needle phobia in an adult IAPT service – a case series. The Cognitive Behaviour Therapist, 13, e51.

Jiang, M. Y., Upton, E., & Newby, J. M. (2020). A randomised wait-list controlled pilot trial of one-session virtual reality exposure therapy for blood-injection-injury phobias. Journal of Affective Disorders, 276, 636- 645.

Lumley, M. A., & Melamed, B. G. (1992). Blood phobics and nonphobics: Psychological differences and affect during exposure. Behaviour research and therapy, 30(5), 425-434.

M. J. (2015). Specific phobias in youth: A randomized controlled trial comparing one-session treatment to a parent-augmented one-session treatment. Behavior therapy, 46(2), 141-155.

Muris, P., Huijding, J., Mayer, B., Langkamp, M., Reyhan, E., & Olatunji, B. (2012). Assessment of disgust sensitivity in children with an age-downward version of the Disgust Emotion Scale. Behavior therapy, 43(4), 876-886.

O’Regan, E., Aynsworth, C., Jones, J., Murphy, E., Powling, R., Galal, U., Grabey, J., Rovira, A., Martin, J., Hollis, C., Clark, D.M., Waite, F., & gameChange Trial Group (2022). Automated virtual reality therapy to treat agoraphobic avoidance and distress in patients with psychosis (gameChange): a multicentre, parallel- group, single-blind, randomised, controlled trial in England with mediation and moderation analyses. Lancet Psychiatry, 9, 375–388.

Oar, E. L., Farrell, L. J., Conlon, E. G., Waters, A. M., & Ollendick, T. H. (2017). Patterns of response and remission following a one-session treatment for blood-injection-injury phobia in youth. Child & Family Behavior Therapy, 39(1), 43-63.

Ollendick, T. H., Halldorsdottir, T., Fraire, M. G., Austin, K. E., Noguchi, R. J., Lewis, K. M., ... & Whitmore,

Öst, L. G., Hellström, K., & Kåver, A. (1992). One versus five sessions of exposure in the treatment of injection phobia. Behavior therapy, 23(2), 263-281.

Silverman, W. K., & Albano, A. M. (1996). Anxiety disorders interview schedule for DSM-IV: Child version. Oxford University Press.

McMurtry, C.M., Taddio, A., Noel, M., Antony, M.M., Chambers, C.T., Asmundson, G.J.G….Scott, J. (2016). Exposure-based interventions for the management of needle fear across the lifespan: a clinical practice guideline and call for further research. Cognitive Behaviour Therapy, 45, 217-235.

Olatunji, B. O., Williams, N. L., Sawchuk, C. N., & Lohr, J. M. (2006). Disgust, anxiety and fainting symptoms associated with blood-injection-injury fears: a structural model. Journal of Anxiety Disorders, 20(1), 23-41.

Lumley, M. A., & Melamed, B. G. (1992). Blood phobics and nonphobics: Psychological differences and affect during exposure. Behaviour research and therapy, 30(5), 425-434.

# Appendices

## Appendix I. Full Mixed-Models and Diagnostic plots.

|  | **IPS** | | | | | **NCQ** | | | | | **DES** | | | | |
| --- | --- | --- | --- | --- | --- | --- | --- | --- | --- | --- | --- | --- | --- | --- | --- |
| **Fixed Effects** | *β* | *SE* | *95%CI* | *T-Statistic* | *p* | *β* | *SE* | *95%CI* | *T-Statistic* | *p* | *β* | *SE* | *95%CI* | *T-Statistic* | *p* |
| Assessment [3W] | -0.43 | 1.21 | -2.79 – 1.92 | -0.36 | 0.722 | 0.13 | 1.52 | -2.82 – 3.08 | 0.09 | 0.930 | 0.60 | 0.70 | -0.75 – 1.95 | 0.86 | 0.389 |
| Assessment [6W] | -0.67 | 1.21 | -3.02 – 1.69 | -0.55 | 0.584 | 1.07 | 1.52 | -1.88 – 4.02 | 0.70 | 0.483 | 0.37 | 0.70 | -0.98 – 1.72 | 0.53 | 0.599 |
| Group [VRT] | -0.03 | 2.67 | -5.24 – 5.17 | -0.01 | 0.990 | 1.93 | 3.21 | -4.33 – 8.19 | 0.60 | 0.548 | 1.50 | 1.59 | -1.59 – 4.59 | 0.95 | 0.346 |
| Assessment [3W] x Group [VRT] | -14.07 | 1.72 | -17.40 – -10.73 | -8.19 | **<0.001** | -11.90 | 2.15 | -16.07 – -7.73 | -5.54 | **<0.001** | -5.23 | 0.98 | -7.14 – -3.32 | -5.32 | **<0.001** |
| Assessment [6W] x Group [VRT] | -14.67 | 1.72 | -18.00 – -11.33 | -8.54 | **<0.001** | -14.97 | 2.15 | -19.14 – -10.79 | -6.97 | **<0.001** | -5.90 | 0.98 | -7.81 – -3.99 | -6.00 | **<0.001** |
| **Random Effects** | | | | | | | | | | | | | | | |
| σ^2^ | 22.10 | | | | | 34.59 | | | | | 7.25 | | | | |
| τ_00_ | 84.74 _ID_ | | | | | 119.97 _ID_ | | | | | 30.47 _ID_ | | | | |
| ICC | 0.79 | | | | | 0.78 | | | | | 0.81 | | | | |
| N | 60 _ID_ | | | | | 60 _ID_ | | | | | 60 _ID_ | | | | |
| Observations | 180 | | | | | 180 | | | | | 180 | | | | |
| Marginal R^2^ / Conditional R^2^ | 0.331 / 0.863 | | | | | 0.192 / 0.823 | | | | | 0.127 / 0.836 | | | | |

Full random-intercept mixed-models for all outcomes are presented in the table below.

Post estimate plots of the model residuals from the linear mixed effects models for the primary and secondary analyses are shown below together with histograms depicting the distribution of the model residuals. Except for the model for the Disgust Emotion Scale, all the other model diagnostics indicate the model assumptions are satisfied.


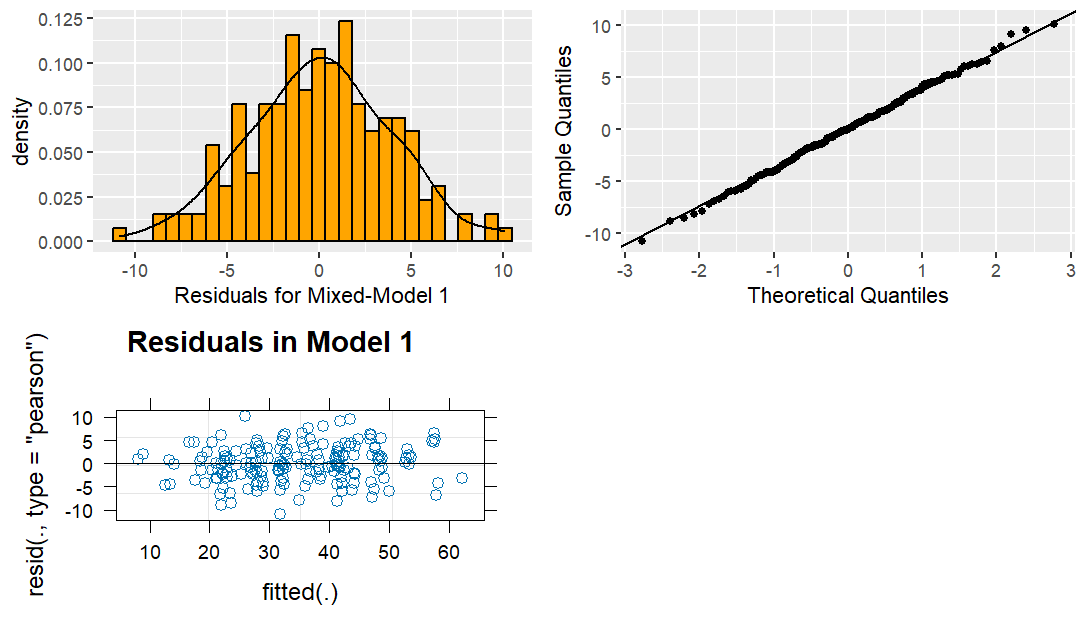


**Mixed-Model diagnostic plots (histogram, normal plot and scatter plot for model residuals) for Model 1: Injection Phobia Scale as the Outcome**


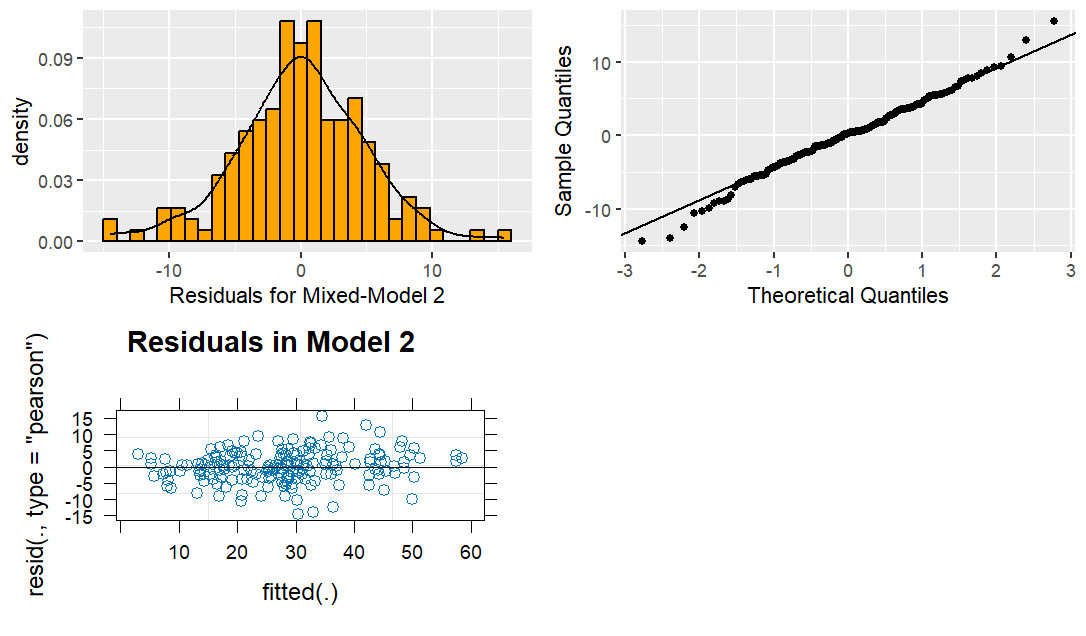


**Mixed-Model diagnostic plots (histogram, normal plot and scatter plot for model residuals) for Model 3: Fearful Needle Cognitions as the Outcome**

##
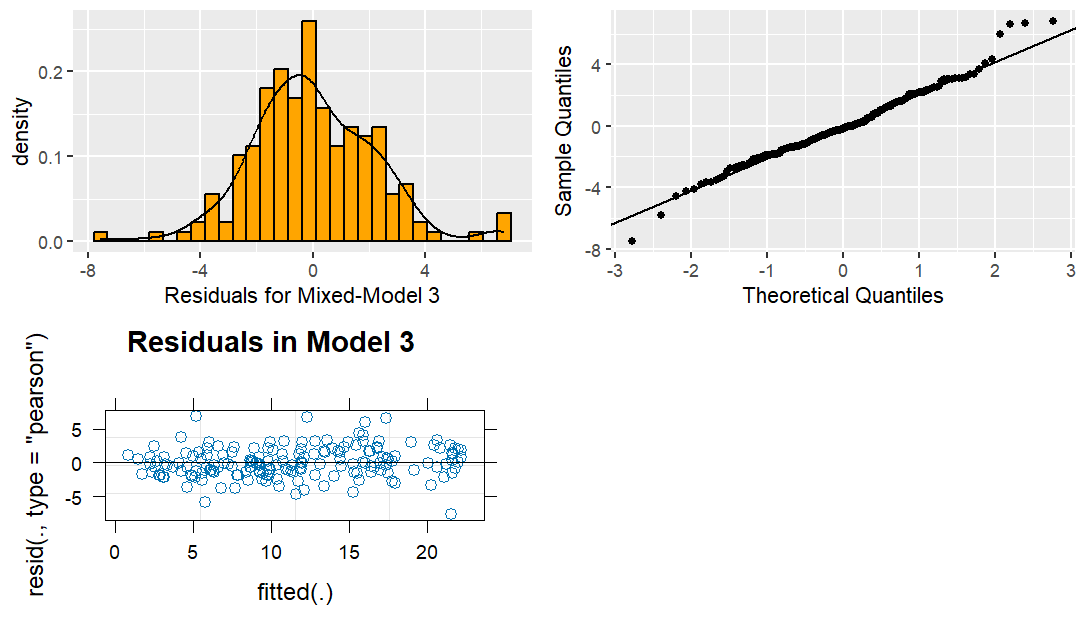


**Mixed-Model diagnostic plots (histogram, normal plot and scatter plot for model residuals) for Model 3: Disgust Emotion Scale as the Outcome**

## Appendix II. SENSITIVITY ANALYSIS

SENSITIVITY ANALYSIS FOR MIXED-MODEL WITH THE SECONDARY OUTCOME EMOTION DISGUST SCALE

|  | **DES** | | | | |
| --- | --- | --- | --- | --- | --- |
| **Fixed-Effects** | *β* | *SE* | *95%CI* | *T-Statistic* | *p* |
| Assessment [3W] | 1.04 | 0.48 | 0.12 – 1.97 | 2.19 | **0.031** |
| Assessment [6W] | 1.00 | 0.48 | 0.08 – 1.92 | 2.10 | **0.038** |
| Group [VRT] | 1.26 | 1.90 | -2.44 – 4.97 | 0.66 | 0.507 |
| Assessment [3W] x Group [VRT] | -6.57 | 0.71 | -7.94 – -5.20 | -9.27 | **<0.001** |
| Assessment [6W] x Group [VRT] | -7.00 | 0.71 | -8.37 – -5.63 | -9.87 | **<0.001** |
| **Random Effects** | | | | | |
| σ^2^ | 2.61 | | | | |
| τ_00_ _ID_ | 34.82 | | | | |
| ICC | 0.93 | | | | |
| N _ID_ | 42 | | | | |
| Observations | 126 | | | | |
| Marginal R^2^ / Conditional R^2^ | 0.141 / 0.940 | | | | |
